# Observation of Dislocation Bound States and Skin Effects in Non‐Hermitian Chern Insulators

**DOI:** 10.1002/adma.202515496

**Published:** 2026-01-27

**Authors:** Jia‐Xin Zhong, Bitan Roy, Yun Jing

**Affiliations:** ^1^ Graduate Program in Acoustics The Pennsylvania State University University Park Pennsylvania USA; ^2^ Department of Physics Lehigh University Bethlehem Pennsylvania USA

**Keywords:** defect, dislocation, Non‐Hermitian physics, topological acoustics

## Abstract

The confluence of non‐Hermitian (NH) topology and crystal defects has culminated in significant interest, yet its experimental exploration has been limited due to the challenges involved in design and measurements. Here, we showcase experimental observation of NH dislocation bound states (NHDS) and the dislocation‐induced NH skin effect in 2D acoustic NH Chern lattices. By embedding an edge dislocations‐antidislocation pair in such acoustic lattices and implementing precision‐controlled hopping and onsite gain/loss via active meta‐atoms, we reveal robust defect‐bound states localized at dislocation cores within the line gap of the complex energy spectrum. We experimentally identify the emergence of bulk exceptional points (EPs) via spectral coalescence and phase rigidity analysis. We demonstrate that the NHDS survive against moderate NH perturbations but gradually delocalize and merge with the bulk (skin) states driven by these EPs under periodic (open) boundary conditions. Furthermore, our experiments demonstrate that the dislocation core can feature weak NH skin effects when its direction is perpendicular to the Burgers vector in periodic systems. Our findings, therefore, pave an experimental pathway for probing NH topology via lattice defects and open new avenues for defect‐engineered topological devices.

## Graphical abstract

Experimental observation of the dislocation‐induced bound states and skin effects is demonstrated in a line‐gap non‐Hermitian acoustic lattice. By utilizing active meta‐atoms to measure the full complex spectrum and biorthogonal eigenstates, the study reveals the exceptional‐point‐driven melting of defect modes. This work unveils the complex interplay between crystal defects and non‐Hermitian topology, offering new pathways for defect‐engineered acoustic devices.

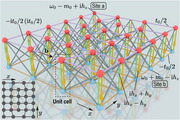

## 1 Introduction

Crystalline defects, such as dislocations [1, 2, 3, 4, 5, 6, 7, 8, 9, 10, 11, 12, 13, 14] and disclinations [15, 16, 17, 18], play pivotal roles in the identification of topological crystals in nature and topological metamaterials in laboratory [19, 20, 21, 22]. In Hermitian topological systems, these defects can host robust bound states, protected by a combination of crystalline symmetry and topological invariant as dictated by the bulk‐defect correspondence, analogous to the celebrated bulk‐boundary correspondence. The emergence of non‐Hermitian (NH) physics, where gain/loss and nonreciprocity play significant roles, introduces radical departures from conventional Hermitian behavior [23, 24, 25, 26, 27, 28, 29, 30, 31, 32, 33]. For instance, NH systems often exhibit phenomena such as the non‐Hermitian skin effect (NHSE), in which a macroscopic number of eigenstates accumulate at the boundaries, obscuring the traditional bulk‐boundary signature [34, 35, 36, 37, 38, 39, 40, 41, 42]. These effects necessitate alternative strategies for detecting and characterizing NH topological phases.

Recent theoretical advances have identified crystalline defects as robust local topological probes in NH systems, potentially overcoming the limitations imposed by the NHSE [38, 43]. Fundamentally, such defect topology can be classified into point‐gap [36, 37] and line‐gap [38] phases. While point‐gap topology is typically characterized solely by the NHSE, line‐gap topology is particularly intriguing as it can host both the dislocation‐induced NHSE (D‐NHSE) and topological bound states, besides displaying conventional NHSE. In this context, Panigrahi et al. theoretically showed that dislocations in a line‐gap NH Chern insulator can host robust in‐gap bound states, protected by pseudo‐particle‐hole symmetry, whose spatial localization and stability are governed by both NH parameters and the defect geometry [38]. Moreover, an interplay between the dislocation and NHSE can give rise to a D‐NHSE, manifesting a typical skin accumulation around the defect core under periodic boundary conditions (PBCs) [36, 37, 38]. However, experimental observation of these fascinating phenomena has remained far from reality due to the requirement for delicate defect engineering combined with precision‐controlled NH perturbations, along with the inherent difficulty in probing complex energy spectrum [44, 45, 46, 47, 48, 49].

In this work, we circumvent these challenges by embedding a pair of edge dislocation and antidislocation into a 2D acoustic lattice implemented using a coupled acoustic cavity system (CACS) and by introducing controlled NH perturbations via active meta‐atoms [35, 49, 50, 51, 52, 53, 54]. Furthermore, the active meta‐atoms are sophistically tuned to induce imaginary hopping terms, thereby breaking the time‐reversal symmetry, an essential requirement for realizaing a Chern insulator. This approach offers a significantly greater flexibility compared to conventional flow‐based methods [55, 56]. By employing a recently proposed Green's function approach [49], we directly measure the complex‐valued energy spectrum along with both left and right eigenstates of the system. Our experimental platform thereby enables the direct observation of NH dislocation states (NHDS) emerging within the line gap of the complex energy spectrum, as well as the D‐NHSE. We further demonstrate that while the NHDS persist under moderate NH perturbations, they lose support in the system with the appearance of the exceptional points (EPs) therein. To validate this mechanism, we explicitly observe the bulk EPs in a pristine lattice, characterized by spectral coalescence and phase rigidity collapse. As this threshold is approached from the weak NH perturbation side, NHDS gradually delocalize and merge into the bulk (skin) states under periodic (open) boundary conditions; a process coinciding with the closure of the NH bandgap, in quantitatively agreement with theoretical predictions. Overall, our work bridges the gap between NH band topology and defect physics, establishing crystal defects as universal tools for probing NH topological phases of matter. We emphasize that our realization of a line‐gap NH Chern insulator enables the observation of the coexistence of D‐NHSE and topological defect‐bound states and their interplay with EPs, phenomena that are fundamentally distinct from the D‐NHSE characteristic of point‐gap systems [57].

## 2 Results and Discussion

### 2.1 Model and Design

**FIGURE 1:**
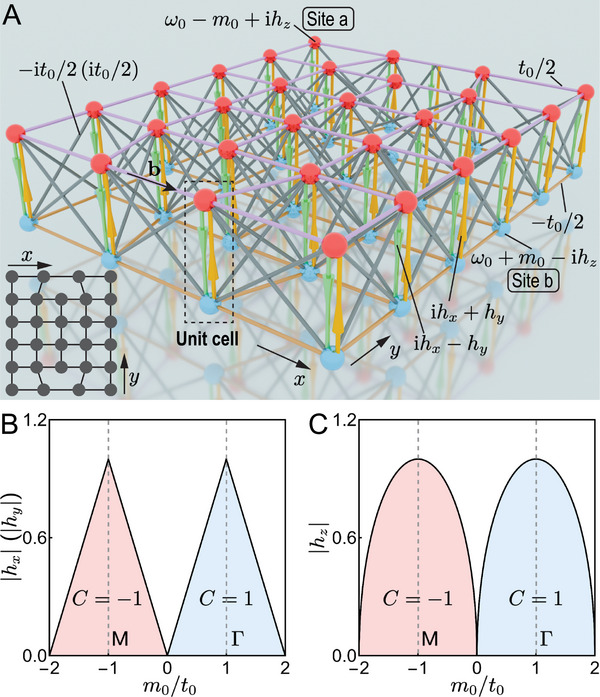
(A) Schematic implementation of a NH Chern insulator [Equation (1)] in the presence of an edge dislocation‐antidislocation pair with Burgers vectors $b=\pm ae_{x}$, where $e_{x}$ is the unit vector in the x direction and a is the lattice spacing, set to be unity. Each unit cell consists of two sites labeled by a (red ball) and b (blue ball). Solid bars of the same color represent the reciprocal hopping amplitudes of equal strength, and those with arrows indicate nonreciprocal hopping amplitudes. Bottom left inset shows the top view, where circles represent unit cells. (B,C) Phase diagram of NH Chern insulators with NH perturbations for (B) $h_{x}$ (or $h_{y}$) and (C) $h_{z}$. The eigenenergies are line‐gapped only in the red and blue shaded regions, where the NH Chern number is C=−1 and C=1, respectively. White regions support EPs. Experimental measurements are performed along the dashed gray lines.

We consider a 2D NH Chern insulator whose Bloch Hamiltonian is expressed as [27, 38, 58]

(1) $$H_{\mathrm{NH}}\left(k\right)=H_{H}\left(k\right)+i\sigma \cdot h=\sigma \cdot \left[d(k)+ih\right]$$

where $d\left(k\right)=(t_{0}\sin\left(k_{x}a\right),t_{0}\sin\left(k_{y}a\right),t_{0}\left[\cos\left(k_{x}a\right)+\cos\left(k_{y}a\right)\right]-m_{0})$, the vector Pauli matrix $\sigma =(\sigma_{x},\sigma_{y},\sigma_{z})$, $i=\sqrt{-1}$ acts on two sublattices a and b, and we set the lattice spacing a=1. The Hermitian part is denoted as $H_{H}\left(k\right)=\sigma \cdot d\left(k\right)$ and the anti‐Hermitian operator is iσ·h with $h=\left(h_{x},h_{y},h_{z}\right)\in R^{3}$. In this model, an edge dislocation‐antidislocation pair is introduced by removing two unit cells located at opposite ends along the y‐direction and subsequently reconnecting the sites along the x‐direction, see Figure 1A. As a result, any closed path encircling the dislocation core lacks a translation by the Burgers vector $b=\pm e_{x}$. The phase diagram of this model is shown in Figure 1B,C for nonzero $h_{x}$ or $h_{y}$ and $h_{z}$, respectively. The NH Chern number is C=−1 in the red‐shaded region and C=1 in the blue‐shaded region, while it is trivial outside these areas. See Appendix Section S1 for details. The system supports topologically protected dislocation states only in the red‐shaded regions [38]. In the Hermitian limit (h=0) the above model features two distinct topological phases for $0<m_{0}/t_{0}<2$ and $-2<m_{0}/t_{0}<0$, respectively, featuring band inversion at the Γ and M points of the Brillouin zone, therefore, named Γ phase and M phase.

**FIGURE 2:**
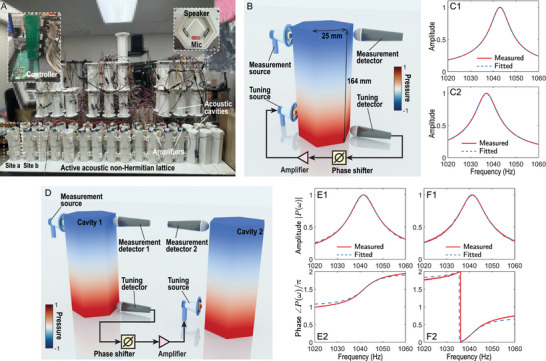
(A) A photograph of the experimental setup. The acoustic non‐Hermitian lattice consists of an array of acoustic cavities, where 56 are used in the final configuration. Onsite potential and hoppings are implemented using microphone‐loudspeaker pair. The hoppings are tuned using phase shifters integrated in a controller. (B) Sketch of tuning onsite potential of acoustic cavities. (C) Measured and fitted magnitude responses for two typical configurations that emulate the sublattices: (C1) sublattice ‘a’ and (C2) sublattice ‘b’ within a unit cell as annotated in Figure 1A. (D) Schematic illustration of the unidirectional hopping implmeneted using a detector and a source. The bottom microphone (tuning detector) in cavity 1 captures the acoustic pressure signal, which is processed by the controller to adjust its phase and amplitude before being emitted by the bottom loudspeaker (tuning source) in cavity 2. Experimentally measured and numerically fitted amplitude (E1 and F1) and phase (E2 and F2) responses of the cross‐power spectral density between the acoustic signals measured in cavities 1 and 2.

We implement this NH Chern insulator using a CACS, as illustrated in Figure 2, which details the experimental setup and parameter tuning methodology. See Experimental Section along with Appendix Section S2 for experimental details, including overall structure and tuning of onsite potentials and hoppings. In our design, each acoustic cavity functions as a lattice site, configured to support a dipole resonant mode at the frequency $\omega_{0}=1040\;\mathrm{Hz}-4.5i\;\mathrm{Hz}$, where the imaginary component represents the intrinsic background loss. It is important to note that $\omega_{0}$ denotes the reference onsite potential engineered for the NH Chern lattice, rather than the intrinsic resonant frequencies of the as‐fabricated cavities. In practice, the natural frequencies of the original cavities deviate significantly due to inevitable fabrication errors and installation imperfections (see Appendix Figure S5). This inherent disorder poses a major challenge to the observation of precise topological phenomena. To overcome this, we employ an active self‐feedback mechanism (Figure 2B) where the signal picked up by a microphone is fed back to a speaker within the same cavity to precisely calibrate and lock the resonance of all 56 cavities to the uniform reference $\omega_{0}$ (see Appendix Section S2.2 for details). In Figure 2C1, the target onsite potential is $\omega_{0}-m_{0}+i\;h_{z}=1043\;\mathrm{Hz}-4.2\;i\;\mathrm{Hz}$, whereas the value extracted by fitting the experimental data is 1042.98Hz−4.21iHz. In Figure 2C2, the target onsite potential is $\omega_{0}+m_{0}-i\;h_{z}=1037\;\mathrm{Hz}-4.8\;i\;\mathrm{Hz}$, with the fitted value of 1037.00Hz−4.81iHz. Here, $m_{0}=-3\;\mathrm{Hz}$ and $h_{z}=0.3\;\mathrm{Hz}$. These two representative examples demonstrate the high precision of our onsite‐potential tuning. Further statistical data regarding the distribution precision and long‐term stability of the onsite potentials across all 56 cavities are presented in Appendix Section S2.5.

As illustrated in Figure 1A, each unit cell comprises two coupled cavities labeled by ‘a’ and ‘b’. The intra‐unit‐cell hopping between these two cavities is rendered by the nonreciprocal NH perturbations, yielding asymmetric hopping amplitudes $ih_{x}-h_{y}$ and $ih_{x}+h_{y}$, while the inter‐unit‐cell hopping amplitudes are reciprocal. Both the nonreciprocal and reciprocal hoppings are realized via detector‐source pairs (i.e., microphone‐loudspeaker systems) [49, 54]. For instance, as shown in Figure 2D, the hopping from site 1 to site 2 is achieved by detecting the acoustic pressure at site 1 and then driving the loudspeaker at site 2 accordingly. Pure imaginary hopping terms ($\pm it_{0}/2$), required to break time‐reversal symmetry for realizing a Chern insulator, are similarly implemented via active components. The magnitude and phase of each microphone‐loudspeaker pair are meticulously adjusted to realize the desired hopping parameters and onsite potentials. For configuration in Figure 2E, the target hopping strength is $\kappa_{0}=t_{0}/2=1.5\;\mathrm{Hz}$, and the retrieved value through fitting experimental results is $\kappa_{0}=1.51\;\mathrm{Hz}+0.01i\;\mathrm{Hz}$. For configuration Figure 2F, the target hopping strength is $\kappa_{0}=-h_{y}+ih_{x}=-0.3\;\mathrm{Hz}+0.3i\;\mathrm{Hz}$, and the retrieved value through fitting experimental results is $\kappa_{0}=-0.30\;\mathrm{Hz}+0.30i\;\mathrm{Hz}$. These two representative examples demonstrate the high precision of our hopping strength tuning. Further statistical data regarding the distribution precision and long‐term stability of the hopping strengths across all 56 cavities are presented in Appendix Section S2.5.

We highlight that this fully programmable active coupling scheme offers distinct capabilities compared to passive acoustic interconnects or topolectrical circuit. Unlike passive tubes typically restricted to reciprocal Hermitian couplings, our active meta‐atoms enable the independent tuning of nonreciprocal terms as well as complex‐valued hoppings that explicitly break time‐reversal symmetry, which are essential for accessing the line‐gap Chern phase. Furthermore, regarding electric circuits, we note a fundamental distinction in physical realization. While circuits are powerful tools for emulating the Hamiltonian matrix structure [59], our platform realizes the Hamiltonian governing the propagation of an actual physical wave field. Consequently, our observation of defect‐core localization and NHSE serves as a direct demonstration of these phenomena in a wave medium, offering valuable insights transferable to other wave‐based platforms such as photonics and mechanics.

### 2.2 Experimental Measurements

Probing the wave dynamics in NH lattices poses unique challenges due to the presence of complex‐valued eigenenergies, which manifest as complex poles in the Green's functions of the system. Therefore, conventional single‐site excitation techniques, relying on the real or simple complex frequency excitations, cannot selectively target these poles [44, 45, 46, 47, 48, 49, 54]. To overcome this limitation, we employ a Green's function‐based measurement method [49]. In our experiments, a source (loudspeaker) is sequentially activated at every site, depicted in Figure 1A, and the resulting frequency responses are simultaneously captured by an array of microphones. This procedure yields the full Green's function G(ω) as a function of the excitation frequency ω. Representative raw spectral responses measured at distinct lattice sites are provided in Appendix Section S3. We note that directly identifying the topological features (NHDS or D‐NHSE) from these raw point‐to‐point spectra is challenging due to the intrinsic background loss, which obscures the specific complex poles. However, by analyzing the full measured Green's function matrix, the complex‐valued eigenenergies and corresponding right and left eigenstates are accurately extracted. Importantly, this Green's‐function‐based approach is universally applicable to general linear wave systems, including those utilizing passive coupling mechanisms (e.g., acoustic tubes). To demonstrate this generality, we successfully applied this method to extract the complex spectrum and eigenstates of a minimal two‐cavity system connected by passive tubes (see detailed validation in Appendix Section S2.8).

### 2.3 Parameter Space

To ensure a comprehensive observation, we choose four distinct values of each NH perturbation in experiments; $h_{y}=h_{z}=0$ and $h_{x}/t_{0}=0.3,0.6,0.9,1.1$, with analogous values for $h_{y}$ and $h_{z}$. We also include the Hermitian case (h=0), resulting in a total of 13 configurations. For each configuration, experiments were performed under open boundary conditions (OBCs) and PBCs in two distinct phases; the M phase with $t_{0}=-m_{0}=3\;\mathrm{Hz}$ and K=(π,π), and the Γ phase with $t_{0}=m_{0}=3\;\mathrm{Hz}$ and K=(0,0). Here, K is the band inversion momentum. In total, 52 cases were explored, with all experimentally measured complex‐valued energy spectra, left and right eigenstates, and corresponding theoretical predictions detailed in the Appendix Section S6 and Figures S20– S71.

### 2.4 Observations of Bulk EPs

**FIGURE 3:**
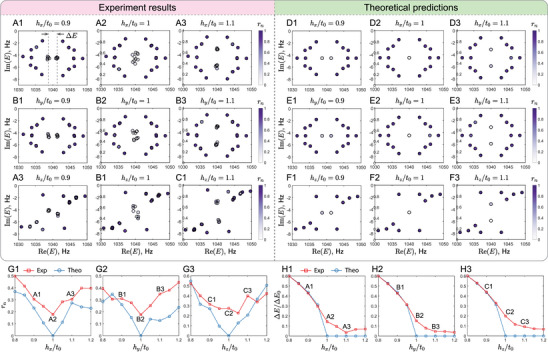
Experimental observation of bulk EPs in a pristine 4×4 acoustic Chern lattice without dislocations under PBCs with NH perturbations for $t_{0}=-m_{0}=3\;\mathrm{Hz}$. (A,B,C) Experimental observations and (D,E,F) corresponding theoretical predictions of the complex energy spectra. The spectra are shown for varying NH perturbation strengths $h_{x}$ (A,D), $h_{y}$ (B,E), and $h_{z}$ (C,F) at values of $0.9t_{0}$ (left), $1.0t_{0}$ (middle), and $1.1t_{0}$ (right). The phase rigidity, $r_{n}$, of the nth state is indicated by the color bar. (G1–G3) Measured (red) and predicted (blue) averaged phase rigidity of all near‐zero‐energy states as a function NH perturbation strength $h_{j}$ for j=x,y,z. (H1–H3) The energy gap, ΔE, defined in (A1) as a function of NH perturbation strength $h_{j}$ for j=x,y,z. Here, $\Delta E_{0}=6\;\mathrm{Hz}$ denotes the line gap in the Hermitian limit ($h_{x}=h_{y}=h_{z}=0$).

Before investigating the interplay between NH topology and dislocations, it is crucial to experimentally validate the emergence of EPs in the bulk lattice. We characterize the spectral properties of a pristine 4×4 lattice without dislocations under PBCs. Figure 3 presents the experimental observation of EP signatures as the NH perturbations ($h_{x},h_{y},h_{z}$) are tuned across the theoretical critical value ($h_{j}/t_{0}=1,j=x,y,z$). Figure 3A–F display the evolution of the complex energy spectra. The discrepancies between the experimental data and the theoretical predictions may arise from disorder in the onsite potentials and hopping amplitudes induced by experimental imperfections (see Appendix Section S4 for details). As the system approaches $h_{c}$, we observe a distinct coalescence of eigenenergies in the complex plane, signaling the proximity to EPs.

To quantitatively verify the EP characteristics, we introduce the phase rigidity $r_{n}$ of the nth eigenstate, defined as

(2) $$r_{n}=\frac{\left|\langle \psi_{n}^{L}|\psi_{n}^{R}\rangle \right|}{\sqrt{\left\langle \psi_{n}^{L}|\psi_{n}^{L}\right\rangle \left\langle \psi_{n}^{R}|\psi_{n}^{R}\right\rangle }}$$

where $|\psi_{n}^{R}\rangle$ and $\left\langle \psi_{n}^{L}\right|$ are the right and left eigenstates corresponding to the nth eigenenergy, respectively. The phase rigidity quantifies the biorthogonal overlap between left and right eigenstates. In a Hermitian system, $r_{n}=1$; however, near an EP, the eigenstates tend to coalesce, leading to a vanishing $r_{n}$. As shown in Figures 3G1–G3, the averaged phase rigidity of all near‐zero‐energy states (crossed by dashed lines in Figure 3A1) exhibits a distinct minimum (dip) around $h/t_{0}=1$. This experimentally confirms that the system undergoes a bulk spectral phase transition driven by EPs.

Furthermore, Figure 3H1–H3 reveal that the real‐energy line gap (ΔE, defined by the separation of two dashed lines in Figure 3A1) starts to close at this critical point. This bulk gap closure provides the physical mechanism for the “melting” of the NHDS observed in the subsequent sections. Since the topological protection of NHDS relies on the existence of an open line gap, its closure at the EP naturally leads to the delocalization of defect modes and their absorption into the bulk continuum.

### 2.5 Observations of NHDS and D‐NHSE

**FIGURE 4:**
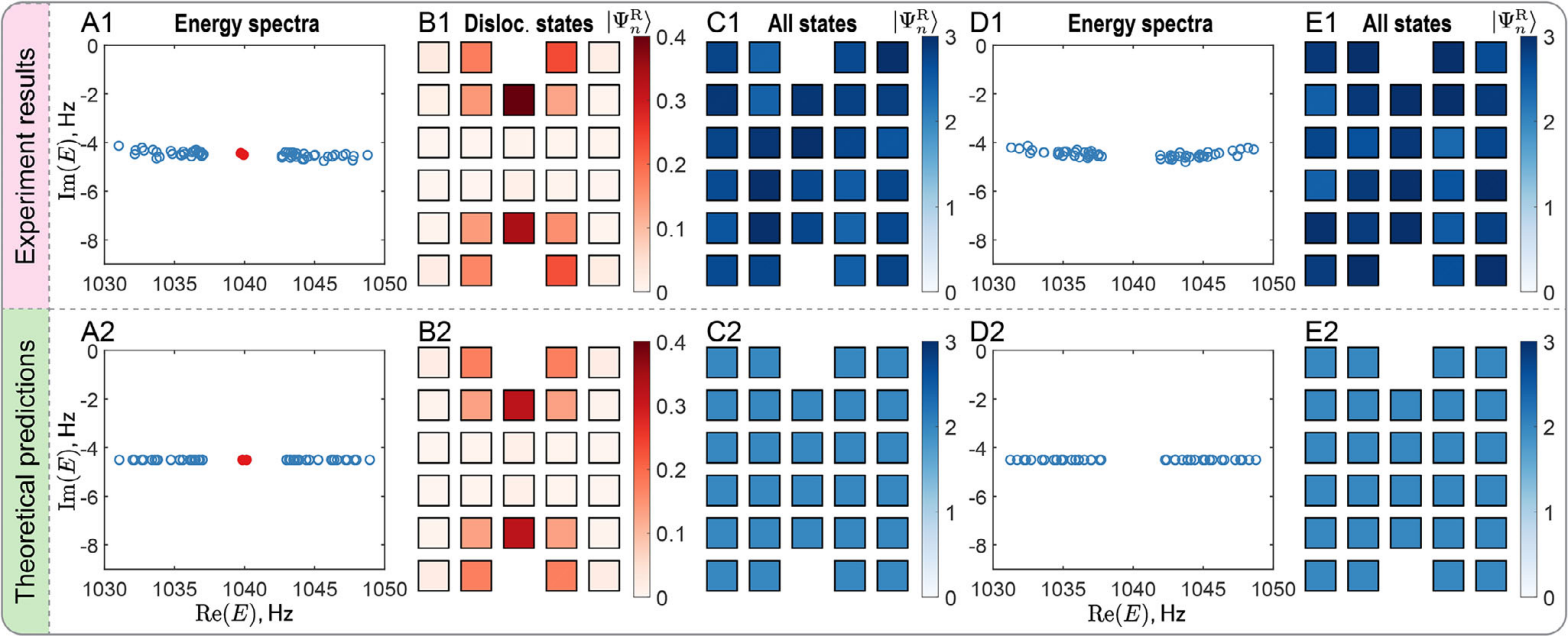
Hermitian (h=0) acoustic Chern insulators in the presence of an edge dislocation‐antidislocation pair under PBCs, showing (A1)‐(E1) experimental observations and (A2)‐(E2) theoretical computations. Energy spectrum [(A1) and (A2)], amplitude distributions of right eigenstates of the dislocation modes, shown by red dots in (A1) and (A2) within unit cells [(B1) and (B2)] and all the states [(C1) and (C2)] in the M phase with $t_{0}=-m_{0}=3\;\mathrm{Hz}$. Energy spectra [(D1) and (D2)] and amplitude distributions of all the right eigenstates [(E1) and (E2)] in the Γ phase with $t_{0}=m_{0}=3\;\mathrm{Hz}$.

We begin our investigation by examining the dislocation bound states in the Hermitian limit (h=0). Figure 4 displays the experimental results for both the M and Γ phases. The measured complex energy spectra and corresponding right eigenstates exhibit excellent agreement with theoretical predictions. Namely, only the M phase (Figure 4A) hosts mid‐gap dislocation states as K·b=±π (nontrivial) around the defect core therein, whereas the Γ phase (Figure 4D) is devoid of such defect modes for which K·b=0 (trivial) [1, 4, 5]. The right eigenstate weight at the n‐th unit cell is defined as 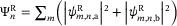, where the index m sums over either the two states near zero energy corresponding to the dislocation states (Figure 4B) or all states (Figure 4C,E). In this notation, $\psi_{m,n,\xi }^{R}$ denotes the right eigenstate for the mth state at site ξ within the nth unit cell, with ξ=a,b. Notably, PBCs are imposed in Figure 4 to suppress midgap edge states, thereby enhancing the visibility of dislocation states. Appendix Figures S21 and S23 show corresponding results under OBCs.

As illustrated in Figure 4A, two distinct midgap states are clearly observed only in the M phase, with their spatial profiles localized at the dislocation cores, see Figure 4B. The dislocation modes are separated from the bulk states, see Figure 4C. By contrast, the Γ phase reveals only bulk eigenstates, with no midgap energies detected, see Figure 4D1, E1. These findings are consistent with the theoretical prediction that a Hermitian Chern insulator hosting a dislocation supports zero‐energy states only in the M phase, where the first Chern number C=−1 [4], see also Figures 4 D2,E2.

**FIGURE 5:**
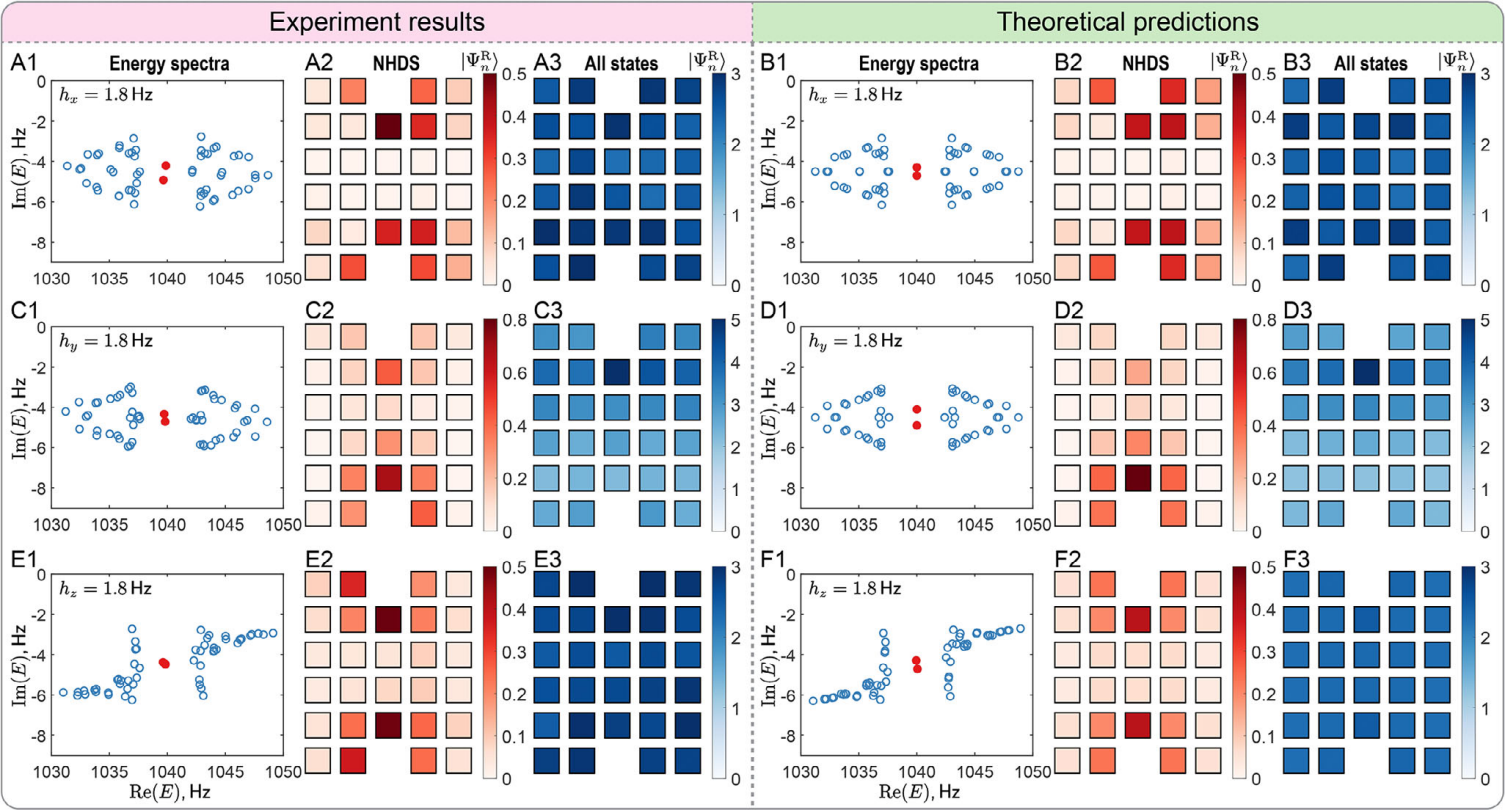
(A,C,E) Experimental observations and (B,D,F) corresponding theoretical predictions on an acoustic Chern lattice with an edge dislocation‐antidislocation pair under PBCs and moderate NH perturbations for $t_{0}=-m_{0}=3\;\mathrm{Hz}$. (A1, B1) Complex energy spectrum for h=(1.8Hz,0,0). (A2,B2) Total amplitude of two right eigenvectors of NHDS marked by red dots in (A1,B1). (A3,B3) Total amplitude of all the right eigenstates from (A1,B1). Panels (C)‐(D) [(E)‐(F)] are analogous to (A)‐(B), respectively, but with h=(0,1.8Hz,0) [h=(0,0,1.8Hz)].

Upon introducing moderate NH perturbations, the energy spectra become considerably more intricate, as shown in Figure 5. Nevertheless, two NHDS remain pinned near zero‐energy within the line gap between 1038 and 1042 Hz (approximately), distinguishing them from the point‐gap associated with typical NHSE in other NH lattices [34]. The spatial distribution of the corresponding eigenstates reveals their pronounced dependence on the NH perturbations in the system. Specifically, Figure 5 A2,B2 show that when only $h_{x}$ is nonzero, the weight of the right eigenstates is biased along the x‐direction, with a greater concentration on the right side (+x) of the dislocation cores. This observation is consistent with the fact that this NH perturbation breaks (preserves) the reflection symmetry about y‐axis (x‐axis). Similarly, Figure 5 C2,D2 depict that for only a nonzero $h_{y}$, the eigenstate weight shifts along the y‐direction, with an enhanced intensity near the bottom dislocation core, as this NH perturbation breaks (preserves) the reflection symmetry about x‐axis (y‐axis). By contrast, when only $h_{z}$ is nonzero, the intrinsic $C_{4}$ symmetry of $H_{H}\left(k\right)$ is preserved, resulting in an approximately symmetric eigenstate distribution along both x and y directions, see Figure 5E2,F2.

Furthermore, the geometry of dislocations (encoded in the Burgers vector) plays a crucial role in determining the fate of the D‐NHSE under PBCs. As shown in Figure 1A, the dislocation core possesses one (two) nearest‐neighbor sites in the y (x) direction when the Burgers vector is $b=\pm e_{x}$, thus mimicking the coordination number of a site living on an y directional boundary. Consequently, under PBCs, no NHSE is observed for nonzero $h_{x}$ near the defect cores, see Figure 5A3,B3 and Appendix Figure S24–S39, while nonzero $h_{y}$ yields a D‐NHSE as shown in Figure 5 C3,D3. However, D‐NHSE is relatively weak compared to the conventional NHSE observed under OBCs at the exterior boundaries of the system. See Appendix Figures S40–S55 for a detailed comparison. Notably, when only $h_{z}$ is nonzero, no NHSE is observed anywhere in the system, irrespective of the boundary conditions, see Figure 5E3,F3 and Appendix Figures S56–S71.

### 2.6 Observations of the NHDS melting

As the strength of NH perturbations increases, initially well‐localized NHDS gradually spread away from the defect cores. Under PBCs, where the conventional NHSE at the exterior boundaries of the system is not supported, the weight of these states is progressively absorbed into the bulk. This trend is observed with different perturbation levels $h_{j}/t_{0}=0.3,0.6,0.9$ for j=x,y,z, as detailed in SI Appendix Figures S24–S35, S40–S51, and S56–S67. A similar behavior is found for the left eigenstates. Nonetheless, in the presence of dislocations, the NH Chern insulators with C=−1 continues to support topologically robust localized states around the defect cores, which happens for $|h_{j}|<t_{0}$ when $|m_{0}|=t_{0}$.

For the parameter setting $t_{0}=-m_{0}=3\;\mathrm{Hz}$, the EPs first appear in the Brillouin zone when $h_{j}=3\;\mathrm{Hz}$ for j=x,y,z, as validated in Figure 3. As the system approaches such a NH bandgap closing, NHDS begin to lose their localization near the defect cores and gradually merge into the bulk states. This melting process is demonstrated in Figure 6, where the system is placed slightly above the critical NH perturbation $h_{j}=3.3\;\mathrm{Hz}$ for j=x,y,z, further detailed in Appendix Figures S39–S39, S52–S55, and S68–S71. In the strong NH perturbation regime, the NHDS near zero‐energy disappear, and only the summed weight of all right eigenstates is presented in Figure 6A2–F2, showing that the localized defect states have fully merged into the bulk under PBC. As further corroborated by experiments under OBCs (see Appendix Figures S37, S53, and S69), the NHDS are found to completely merge into the skin states living at the exterior boundaries of the system. Collectively, Figures 5 and 6, and the Appendix Figures S24–S71, provide clear evidence for a localization‐delocalization phase transition in the NHDS, a phenomenon exclusively observable in NH systems.

**FIGURE 6:**
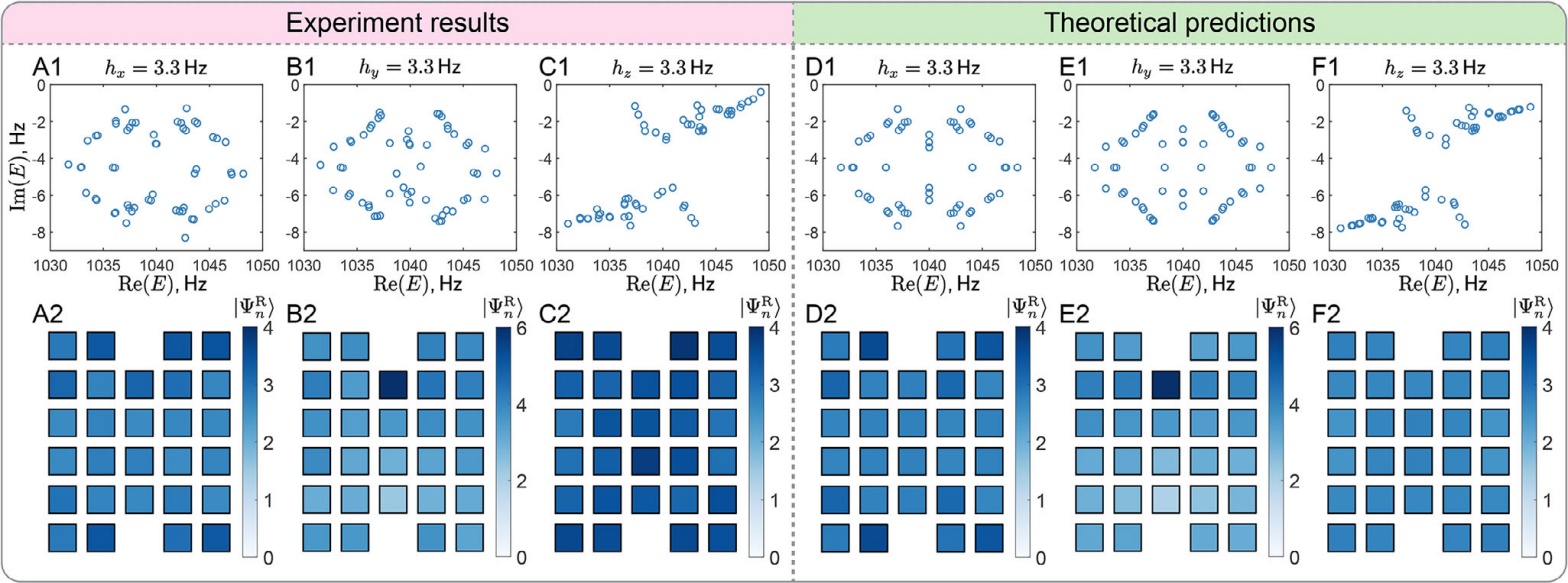
Similar to Figure 5, but for *strong* NH perturbations, showing the absence of NHDS. Complex energy spectrum for (A1,D1) h=(3.3Hz,0,0) (B1,E1) h=(0,3.3Hz,0), and (C1,F1) h=(0,0,3.3Hz). Corresponding total amplitude of all the right eigenstates are shown in (A2,D2), (B2,E2), and (C2,F2), respectively. (A,B,C) Experimental observations and (D,E,F) corresponding theoretical predictions.

It is noteworthy that Figure 6B2,E2 illustrate a pronounced D‐NHSE in the absence of NHDS for stronger $h_{y}$, with a higher right eigenstate weight at the top dislocation core compared to that for a weaker $h_{y}$, shown in Figure 5C3,D3. By contrast, for nonzero $h_{x}$ and $h_{z}$, the D‐NHSE is either absent (likelihood) or much weaker, as shown in Figure 6A2,C2, respectively. Therefore, our experimental observations clearly demonstrate a D‐NHSE that is distinct from the conventional point‐gap‐supported NHSE under OBCs [34], and it depends on the relative orientation of the Burgers vector and the directionality of the NHSE.

### 2.7 Observations Under OBCs

**FIGURE 7:**
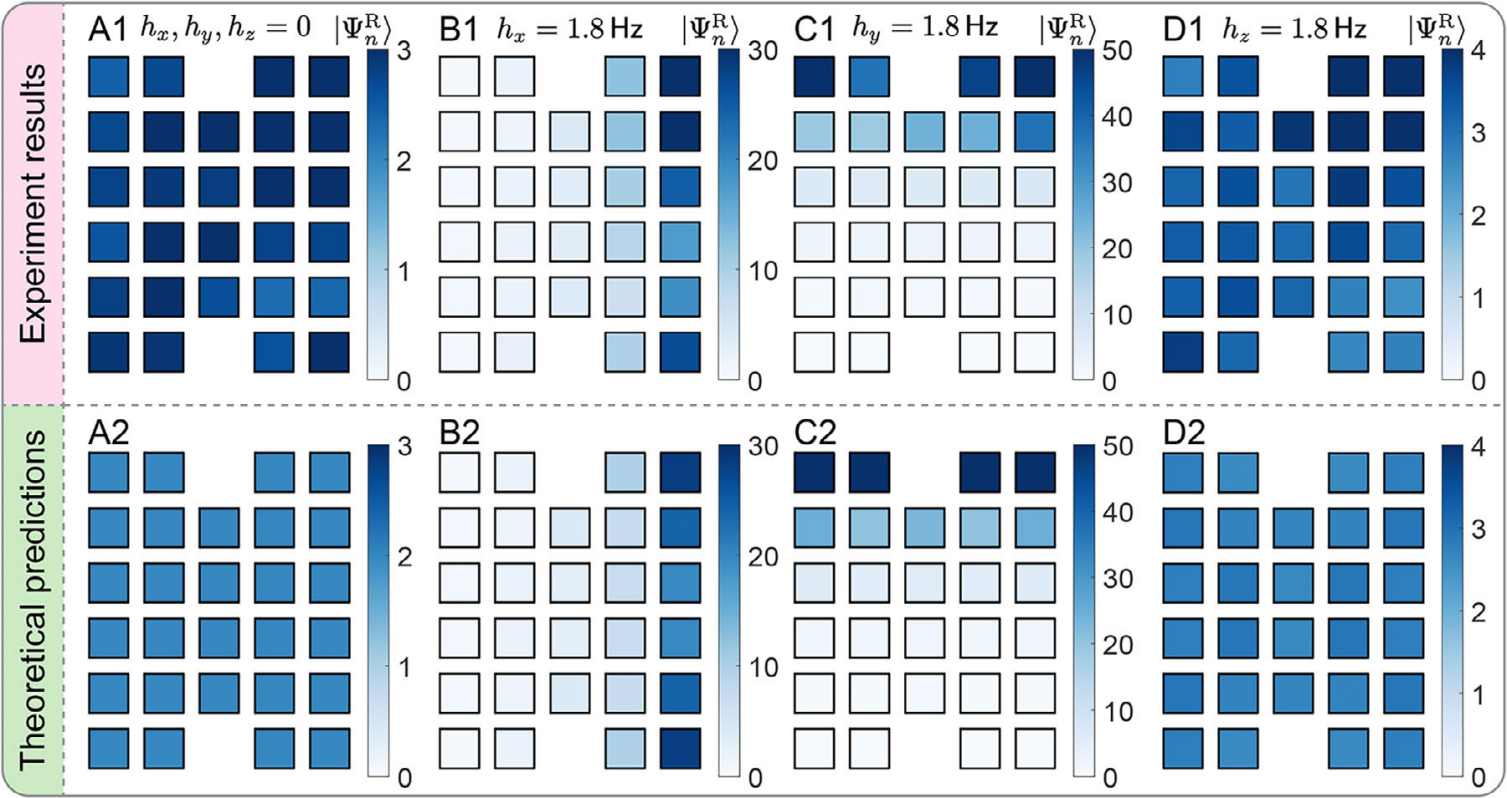
(A1–A4) Experimental observations and (B1–B4) theoretical predictions of the total amplitude of all right eigenstates under OBCs in the M phase with $t_{0}=-m_{0}=3\;\mathrm{Hz}$ for (A) h=(0,0,0) (Hermitian limit), (B) h=(1.8Hz,0,0), (C) h=(0,1.8Hz,0), and (D) h=(0,0,1.8Hz).

Under OBCs, the spatial distribution of the summed weight of all right eigenstates reveals valuable insights. For a Hermitian insulator and a NH one with nonzero $h_{z}$, such a weight extends uniformly throughout the entire lattice, as shown in Figure 7A,D, respectively, confirming the absence of any NHSE therein. However, with the NH perturbations $h_{x}$ and $h_{y}$, clear signatures of NHSE emerge, with the right wavefunctions localizing toward the positive x and y boundaries, respectively, shown in Figure 7B,C. This anisotropic behavior stems from the broken reflection and inversion symmetries by the NH perturbations $h_{x}$ and $h_{y}$. With the emergence of NHSE along the edges, the dislocation cores themselves do not exhibit a pronounced NHSE under OBCs for nonzero $h_{x}$ and $h_{y}$. By contrast, when the Burgers vector is perpendicular to the direction of the NHSE observed under OBCs, a D‐NHSE becomes visible under PBC, as show in Figure 6B2,E2.

## 3 Conclusion

To summarize, our experiments provide conclusive evidence for NHDS and D‐NHSE in a 2D acoustic Chern insulator. In the Hermitian limit, dislocations host zero‐energy states in the M phase, consistent with the bulk‐defect correspondence [1, 4, 5]. Under moderate NH perturbations, these states persist within the line gap. As the perturbation strength increases, we observe a localization‐delocalization transition for NHDS as they gradually spread and merge with bulk states under PBCs when the system approaches the shore of hosting EPs in the Brillouin zone [38]. By contrast, the D‐NHSE appears for any arbitrary strength of NH perturbations, when the direction of the conventional NHSE is orthogonal to the Burgers vector.

Beyond their fundamental significance, these phenomena point toward specific applications in acoustic device engineering. For instance, the NHDS, which exhibits line‐gap protected localization only within a specific spectral window, can serve as the basis for frequency‐selective acoustic concentrators or filters. Conversely, the D‐NHSE, which funnels bulk energy toward the dislocation core, suggests a mechanism for high‐sensitivity sensing. The resulting intensity accumulation at the defect core can naturally amplify the system's response to minute local perturbations, such as mass loading or structural defects. Our results not only bridge the gap between NH band topology and defect physics but also establish crystalline defects as a universal tool for probing NH topological matter.

## 4 Experimental Section

### 4.1 Experimental setup

Figure 2A shows a photograph of our experimental setup. The system consisted of 56 3D‐printed acoustic cavities, each representing a site in the NH lattice. A custom‐designed digital controller was employed to precisely tune the onsite potentials of the acoustic cavities and the hoppings between them. The tuning of onsite potentials and hoppings is implemented using active components, specifically microphone‐loudspeaker (detector‐source) pairs. The hopping implementations were independent of spatial distances between sites, enabling flexible implementation of various boundary conditions and lattice geometries with ease.

The custom‐made digital controller comprises three components: the core board, the motherboard, and the input/output (IO) board. The core board houses a field‐programmable gate array (FPGA, XC7K325T, Xilinx) and a digital signal processor (DSP, TMS320C6678, Texas Instruments), which enable real‐time signal processing. During experiments, the system operated at a sampling frequency of 12.8 kHz. The motherboard supplied power and facilitates communication between the core and IO boards. The IO board handles analog signal acquisition and output, interfacing with microphones and loudspeakers, respectively. Each IO board supports 16 channels for both analog inputs and outputs. The input channels utilize analog‐to‐digital converters (ADCs, ADC7606B, ADI), and the output channels employ digital‐to‐analog converters (DACs, DAC8568, Texas Instruments).

### 4.2 Acoustic Cavities and Tuning of Onsite Potentials

Appendix Section S2.2 shows the tuning process of the onsite potentials of acoustic cavities. The acoustic cavities used in this study were fabricated using 3D printing with a tolerance of 0.2 mm or within 0.3%. The material was LEDO 6060 photosensitive resin, which behaves as acoustically rigid for airborne sound. Each printed cavity had a wall thickness of 6 mm. As shown in Figure 2B, the cavities were hexagonal prisms with an interior height of l=164mm and a side length of 25mm. To characterize the cavity in the experiments, a loudspeaker (source) excites the cavity, and a microphone (detector) measures the acoustic pressure. The onsite potential, $\omega_{0}$, of a cavity is retrieved using the Green's function for a single site:

(3) $$G_{0}\left(\omega \right)=-\frac{ℑ(\omega_{0})}{\omega -\omega_{0}}$$

where ω is the excitation frequency.

### 4.3 Tuning of Hoppings

Figure 2D shows the tuning process of the hoppings. Both nonreciprocal and reciprocal hoppings between the cavities were implemented using detector‐source (microphone‐loudspeaker) pairs. In our platform, the hopping strength and phase were precisely controlled by a digital multi‐channel controller, allowing flexible, and reconfigurable manipulation of lattice hoppings. Each unidirectional hopping was realized through a loudspeaker, a microphone, and an audio amplifier (Texas Instruments LM386). The loudspeaker and microphone were positioned at the bottom of each cavity. The microphone captures the acoustic pressure signal, which was processed by the controller to adjust its phase and amplitude before being emitted by the loudspeaker in the connected cavity. The analytical solution of this model is described in Appendix Section S2.4. Crucially, to avoid self‐oscillation, we actively verify that the system remains in the stable regime by confirming that all eigenenergies in the reconstructed complex spectrum possess negative imaginary parts (see Appendix Section S2.6 for details).

The tuning of the hopping parameters was performed as follows. As illustrated in Figure 2D, a microphone is placed inside cavity 1 to detect the acoustic pressure. The detected pressure signal was then phase‐adjusted, amplified, and emitted by the loudspeaker positioned in cavity 2. To determine both the amplitude and the phase of the unidirectional hopping, we excite at cavity 1 using a loudspeaker (measurement source) and measure the acoustic pressure in both cavities using two microphones (measurement detector). The cross‐power spectral density of these two measured pressure signals is expressed by

(4) $$P\left(\omega \right)\equiv \frac{\psi_{2}\left(\omega \right)}{\psi_{1}\left(\omega \right)}=\frac{\kappa_{0}}{\omega -\omega_{0}}$$

where $\kappa_{0}$ is the tuned hopping.

### 4.4 Implementation of Boundary Conditions

Leveraging the fully programmable nature of our active coupling scheme, we can seamlessly switch between PBCs and OBCs without physical reconfiguration of the acoustic structure. Since all hoppings were implemented via microphone‐loudspeaker pairs connected through digital circuits, PBCs were realized by electronically enabling the feedback loops that connect the boundary sites across the lattice edges. Conversely, OBCs were achieved simply by disabling these specific boundary coupling channels in the controller. A schematic illustration using a simplified 1D chain model is provided in Appendix Section S2.7 to clarify this implementation.

### 4.5 Measurement Process

To obtain the full Green's function matrix, an acoustic source (loudspeaker) was sequentially excited at each cavity site, and the acoustic pressure was measured in all cavities using microphones. This procedure was systematically repeated for every site across the entire lattice. The complex‐valued energy spectra, as well as left and right eigenstates of NH acoustic Chern insulators were obtained based on the method proposed in Ref. [49].

## Note Added

During the review process of this manuscript, we became aware of a related work by Wu et al. [57]. We note that the two works explore distinct and complementary regimes of non‐Hermitian defect physics. Wu et al. investigate the point‐gap topology in a 2D Hatano‐Nelson lattice, focusing on the D‐NHSE. Conversely, our work realizes a line‐gap non‐Hermitian Chern insulator, which enables the observation of the coexistence of D‐NHSE and topological defect‐bound states (NHDS), as well as the interplay with EPs. Collectively, these studies establish a comprehensive experimental framework for understanding defect engineering across different non‐Hermitian topological classes.

## Conflicts of Interest

The authors declare no conflicts of interest.

## Supporting information


**Supporting File**: adma72230‐sup‐0001‐SuppMat.pdf.

## Data Availability

The data that support the findings of this study are available from the corresponding author upon reasonable request.
