# Supplementary material for: Observation of Dislocation Bound States and Skin Effects in Non‐Hermitian Chern Insulators
Source: Adv Mater. 2026 Jan 27;38(13):e15496. doi: 10.1002/adma.202515496 (PMC12957872; doi:10.1002/adma.202515496)
Supplement: Supplementary file 1 — Supporting File: adma72230‐sup‐0001‐SuppMat.pdf. [file ADMA-38-e15496-s001.pdf]

Supplementary Materials

“Observation of dislocation bound states and skin effects in non-Hermitian Chern insulators”

Jia-Xin Zhong[1], Bitan Roy*[2], and Yun Jing†[1]

[1]Graduate Program in Acoustics, The Pennsylvania State University, University Park, PA 16802, USA
[2]Department of Physics, Lehigh University, Bethlehem, Pennsylvania 18015, USA

Contents

S1 Phase diagram of NH Chern insulators **2**

S2 Experimental details **4**

S2.1 Overall structure and measurement process 4
S2.2 Acoustic cavities and tuning of onsite potentials 5
S2.3 Tuning of hoppings 6
S2.4 The coupled-model theory for the unidirectional coupling of two coupled acoustic cavities 8
S2.5 Statistics and long-term stability of onsite potential and hopping tuning 9
S2.6 Audio amplifiers and self-oscillation issues 13
S2.7 Implementation of PBCs and OBCs 13
S2.8 Validation of complex spectrum and eigenstate extraction via passive acoustic tubes 15

S3 Measured Green’s function responses and reconstruction of complex spectrum and eigenstates **18**

S4 Characterizing bulk EPs in a pristine 4×4 lattice **20**

S5 Phase rigidity analysis near bulk EPs for the 5×6 lattice with dislocation **22**

S6 Extended experimental results **24**

References **53**

*bitan.roy@lehigh.edu
†yqj5201@psu.edu

S1 Phase diagram of NH Chern insulators

In this section of the Supplemental Material, we discuss the phase diagram of the Bloch Hamiltonian for 2D non-Hermitian (NH) Chern insulator given by

$$H_{\rm NH}(\mathbf{k}) = H_{\rm H}(\mathbf{k}) + {\rm i}\boldsymbol{\sigma}\cdot\mathbf{h} = \boldsymbol{\sigma}\cdot[\mathbf{d}(\mathbf{k}) + {\rm i}\mathbf{h}] \equiv \boldsymbol{\sigma}\cdot\mathbf{d}_{\rm NH}(\mathbf{k}), \tag{S1}$$

where

$$\mathbf{d}(\mathbf{k}) = (t_0 \sin k_x + {\rm i}h_x, t_0 \sin k_y + {\rm i}h_y, t_0[\cos k_x + \cos k_y] - m_0 + {\rm i}h_z)\,. \tag{S2}$$

The phase diagram of this model is determined from the NH Chern number given by

$$C = {\rm Re}\int_{\rm BZ} \frac{d^2\mathbf{k}}{4\pi}\left[\partial_x \hat{\mathbf{d}}_{\rm NH}(\mathbf{k}) \times \partial_y \hat{\mathbf{d}}_{\rm NH}(\mathbf{k})\right]\cdot \hat{\mathbf{d}}_{\rm NH}(\mathbf{k}), \tag{S3}$$

where $\partial_j \equiv \partial_{k_j}$, $\mathbf{d}_{\rm NH}(\mathbf{k}) = \mathbf{d}(\mathbf{k}) + {\rm i}\mathbf{h}$, and $\hat{\mathbf{d}}_{\rm NH}(\mathbf{k}) = \mathbf{d}_{\rm NH}(\mathbf{k})/\sqrt{\mathbf{d}^2_{\rm NH}(\mathbf{k})}$. For $\mathbf{h} = 0$, we recover the standard definition of the Chern number. The integration is performed within the first Brillouin zone.

For the computation of C, instead of relying on the numerical integration, we convert it into a summation over discrete values of momentum $\mathbf{k} = (k_i, k_j)$, leading to

$$C = {\rm Re}\sum_{k_i,k_j} \frac{4\pi^2}{N_{xy}}\left\{\left[\partial_x \hat{\mathbf{d}}_{\rm NH}(k_i,k_j) \times \partial_y \hat{\mathbf{d}}_{\rm NH}(k_i,k_j)\right]\cdot \hat{\mathbf{d}}_{\rm NH}(k_i,k_j)\right\} \tag{S4}$$

where $k_i = \pi i/N_x$ and $k_j = \pi j/N_y$ with $i = -N_x, -N_x+1, \cdots, N_x-1, N_x$ and $j = -N_y, -N_y + 1, \cdots, N_y-1, N_y$. We always choose $N_x = N_y = N$ and $N_{xy} = N_x N_y = N^2$. In the Hermitian system ($\mathbf{h} = 0$), the above summation expression for the Chern number converges to the desired quantized values (within numerical accuracy up to two decimal points) with $N = 10^5$. When the NH parameter values are such that the system is far from the EPs, as are the cases with $t_0 = \pm m_0 = 1$ and $\mathbf{h} = (0.3,0,0), (0,0.3,0), (0,0,0.3)$ or $\mathbf{h} = (0.6,0,0), (0,0.6,0), (0,0,0.6)$, the Chern number converges to integer quantized values (within two decimal points) for $N = 5\times 10^5$. However, as the system approaches the EPs, the number of grid points (N) needs to be increased to obtain quantized Chern number. For example, $t_0 = \pm m_0 = 1$ and $\mathbf{h} = (0.9,0,0), (0,0.9,0), (0,0,0.9)$, quantized (up to two decimal point) Chern numbers are obtained for $N = 2\times 10^6$.

The phase diagram of $H_{\rm NH}(\mathbf{k})$, obtained in terms of C is shown in Fig. S1. For h_x or h_y, the red shaded region in Fig. S1(a) where $C = -1$ is determined by

$$\begin{cases} |h_x| < |m_0 + 2|, & -2 \le m_0/t_0 \le -1 \\ |h_x| < |m_0|, & -1 \le m_0/t_0 \le 0. \end{cases} \tag{S5}$$

On the same phase diagram the blue shaded region where $C = 1$ is determined by

$$\begin{cases} |h_x| < |m_0 - 2|, & 1 \le m_0/t_0 \le 2 \\ |h_x| < |m_0|, & 0 \le m_0/t_0 \le 1. \end{cases} \tag{S6}$$

On the other hand, for h_z, the red shaded region in Fig. S1(b) where $C = -1$ is determined by

$$|h_z| < \sqrt{2|m_0| - m_0^2}, \quad -2 \le m_0/t_0 \le 0. \tag{S7}$$

Whereas the blue shaded region where $C = 1$ is determined by

$$|h_z| < \sqrt{2|m_0| - m_0^2}, \quad 0 \le m_0/t_0 \le 2. \tag{S8}$$

Outside these parameter regimes, shown by the white shaded regions in Fig. S1, the NH Chern number is trivial. Respectively, in the red and blue shaded regions the inversion of the NH and Hermitian (when $\mathbf{h} = 0$) bands occur near the M and Γ points of the Brillouin zone.

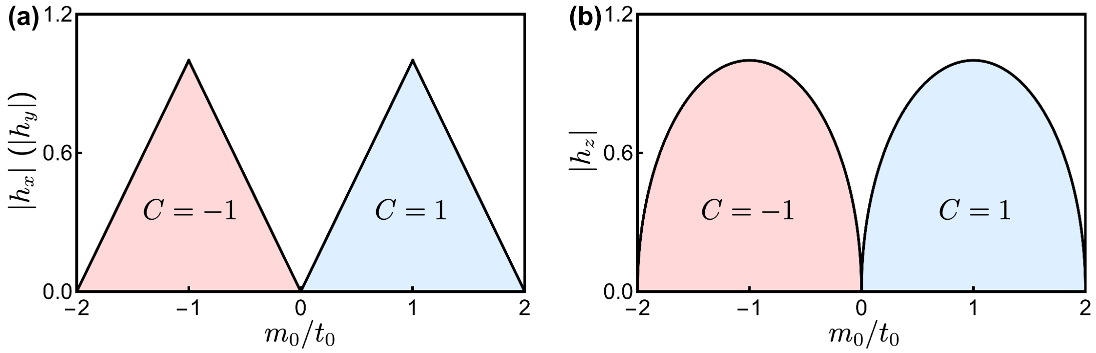

Figure S1: Phase diagram of NH Chern insulators with NH perturbations for (a) h_x (or h_y) and (b) h_z. The eigenenergies are line-gapped only in the red and blue shaded regions, where the NH Chern number is $C = -1$ and $C = 1$, respectively. The system supports topologically protected dislocation states only in the red shaded regions.

S2 Experimental details

This section of the Supplemental Material is devoted to discuss various key details related to the implementation of NH model on acoustic lattice and measurement protocols.

S2.1 Overall structure and measurement process

Figure S2 shows a photograph of our experimental setup. The system consists of 56 3D-printed acoustic cavities, each representing a site in the NH lattice. A custom-designed digital controller is employed to precisely tune the onsite potentials of the acoustic cavities and the hoppings between them, as detailed in Secs. S2.2 and S2.3, respectively. The tuning of onsite potentials and hoppings is implemented using active components, specifically microphone-loudspeaker (detector-source) pairs. The hopping implementations are independent of spatial distances between sites, enabling flexible implementation of various boundary conditions and lattice geometries with ease.

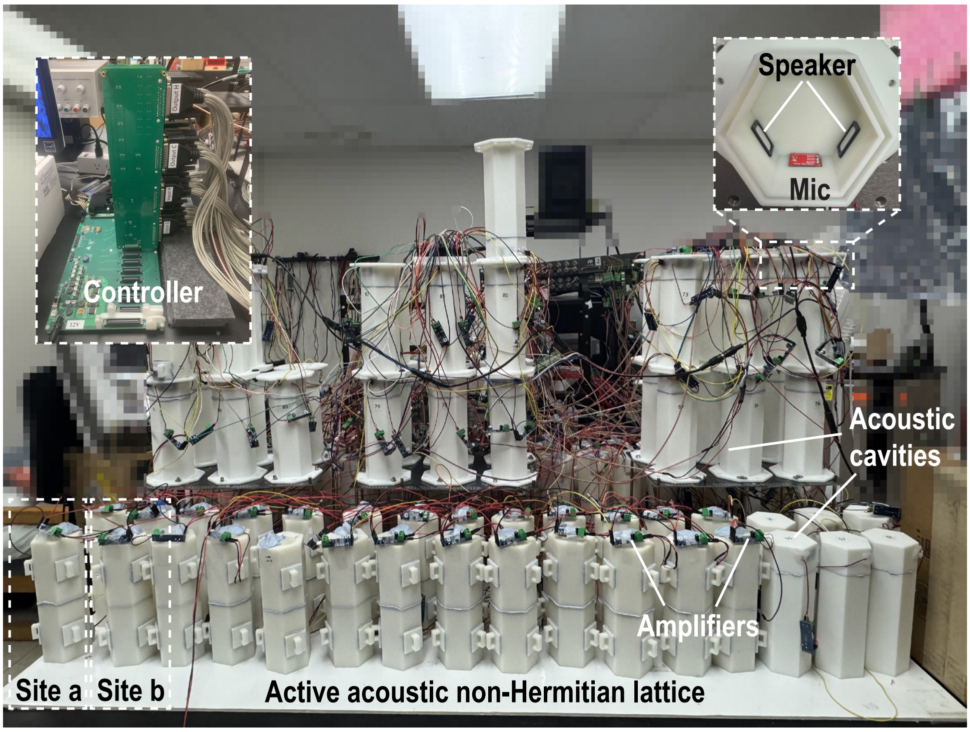

Figure S2: Photo of the experimental setup. The phononic non-Hermitian lattice consists of 90 acoustic cavities, where 56 are used in the final configuration. Onsite potential and hoppings are implemented using microphone-loudspeaker pair. The magnitude and phase of hoppings are tuned using a real-time digital controller. A data acquisition (DAQ) system is programmed to measure the Green's function for all possible combinations of source-detector positions across the entire lattice.

The custom-made digital controller comprises three components: the core board, the motherboard, and the input/output (IO) board. The core board houses a field-programmable gate array (FPGA, XC7K325T, Xilinx) and a digital signal processor (DSP, TMS320C6678, Texas Instruments), which enable real-time signal processing. During experiments, the system op-

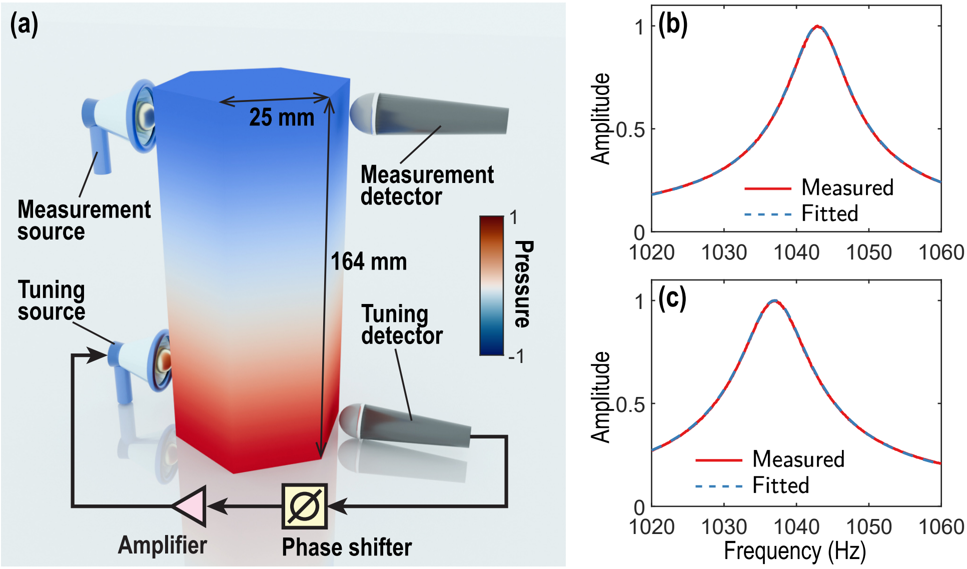

Figure S3: Tuning onsite potential (resonant frequency) of acoustic cavities. (a) Schematic illustration of the tuning process. The air column is designed as a hexagonal prism with a height of 164 mm and a side length of 25 mm. (b–c) Measured and fitted magnitude responses for two typical configurations that emulate the sublattices: (b) sublattice 'a' and (c) sublattice 'b' within a unit cell as annotated in Fig. 1A of the main text. In (b), the target onsite potential is $\omega_0 - m_0 + \mathrm{i}h_z = 1043\,\mathrm{Hz} - 4.2\,\mathrm{i}\,\mathrm{Hz}$, and the value obtained from fitting experimental data is $1042.98\,\mathrm{Hz} - 4.21\,\mathrm{i}\,\mathrm{Hz}$. In (c), the target onsite potential is $\omega_0 + m_0 - \mathrm{i}h_z = 1037\,\mathrm{Hz} - 4.8\,\mathrm{i}\,\mathrm{Hz}$, with the fitted value being $1037.00\,\mathrm{Hz} - 4.81\,\mathrm{i}\,\mathrm{Hz}$. Other parameters are $\omega_0 = 1040\,\mathrm{Hz} - 4.5\,\mathrm{i}\,\mathrm{Hz}$, $m_0 = -3\,\mathrm{Hz}$, and $h_z = 0.3\,\mathrm{Hz}$.

erates at a sampling frequency of 12.8 kHz. The motherboard supplies power and facilitates communication between the core and IO boards. The IO board handles analog signal acquisition and output, interfacing with microphones and loudspeakers, respectively. Each IO board supports 16 channels for both analog inputs and outputs. The input channels utilize analog-to-digital converters (ADCs, ADC7606B, ADI), and the output channels employ digital-to-analog converters (DACs, DAC8568, Texas Instruments).

To obtain the full Green's function matrix, an acoustic source (loudspeaker) is sequentially excited at each cavity site, and the acoustic pressure is measured in all cavities using microphones. This procedure is systematically repeated for every site across the entire lattice. The complex-valued energy spectra as well as left and right eigenstates of NH acoustic Chern insulators are obtained based on the method proposed in Ref. [1].

S2.2 Acoustic cavities and tuning of onsite potentials

Figure S3 shows the tuning process of the onsite potentials of acoustic cavities. The acoustic cavities used in this study were fabricated using 3D printing with a tolerance of 0.2 mm or within 0.3%. The material is LEDO 6060 photosensitive resin, which behaves as acoustically rigid for airborne sound. Each printed cavity has a wall thickness of 6 mm. As shown in Figs. S2 and S3, the cavities are hexagonal prisms with an interior height of $l = 164\,\mathrm{mm}$ and

a side length of 25 mm. To characterize the cavity in the experiments, a loudspeaker (source) excites the cavity, and a microphone (detector) measures the acoustic pressure, as illustrated in Fig. S3(a). The onsite potential, ω_0, of a cavity is retrieved using the Green's function for a single site:

$$G_0(\omega) = -\frac{\mathrm{Im}(\omega_0)}{\omega - \omega_0}, \tag{S9}$$

where ω is the excitation frequency.

The original onsite potential of the cavity is denoted by a complex number ω_0 with real part representing the resonant frequency and imaginary part representing the intrinsic loss. To tune the onsite potential to a target value $\omega_0^{\mathrm{tgt}} = \omega_0 + \Delta\omega_0$, we use an additional microphone-loudspeaker pair connected through a feedback circuit to form an active control loop, as illustrated in Fig. S3(a). By doing the fitting using Eq. (S9), we extract that the original onsite potential of the cavity, e.g., $\omega_0 = 1040\,\mathrm{Hz} - 4.5\,\mathrm{i}\,\mathrm{Hz}$.

To tune the onsite potential to the target value (e.g., $\omega_0^{\mathrm{tgt}} = 1043\,\mathrm{Hz} - 4.2\,\mathrm{i}\,\mathrm{Hz}$), we set the the amplifier to be unit gain $\left|g^{(1)}\right| = 1$ and adjust the phase shift to be $\angle g^{(1)} = 0$. We can measure the frequency response of the cavity again and fit it using Eq. (S9) to extract the new onsite potential. For example, this value might be $\omega_0^{(1)} = 1041.9\,\mathrm{Hz} - 4.7\,\mathrm{i}\,\mathrm{Hz}$ after the first tuning. Then, the difference between the new onsite potential and the original one is calculated as $\Delta\omega_0^{(1)} = 1.1\,\mathrm{Hz} + 0.5\,\mathrm{i}\,\mathrm{Hz}$. This value depends on the specific sensitivites of the whole feedback loop, which can vary at different sites.

The next step is to calculate the required gain factor $g^{(2)}$ to reach the target onsite potential ω_0^{tgt}:

$$g^{(2)} = \frac{\omega_0^{\mathrm{tgt}} - \omega_0}{\Delta\omega_0^{(1)}}. \tag{S10}$$

By repeating the measurement of the frequency of the cavity with this gain factor $g^{(2)}$, we can obtain a new onsite potential $\omega_0^{(2)}$ and confirm whether it reaches the target value ω_0^{tgt}. If not, we can repeat the above process iteratively until the onsite potential converges to the target value ω_0^{tgt} within a desired precision (0.1 Hz in our experiments). Figures S3(b) and (c) show two examples of the measured frequency responses and the fitted onsite potentials for two sublattices in our acoustic NH Chern lattice. We can see that the fitted onsite potentials are very close to the target values, which confirms the effectiveness of our tuning method.

S2.3 Tuning of hoppings

Figure S4 shows the tuning process of the hoppings. Both nonreciprocal and reciprocal hoppings between the cavities are implemented using detector-source (microphone-loudspeaker) pairs. In our platform, the hopping strength and phase are precisely controlled by a digital multi-channel controller, allowing flexible and reconfigurable manipulation of lattice hoppings.

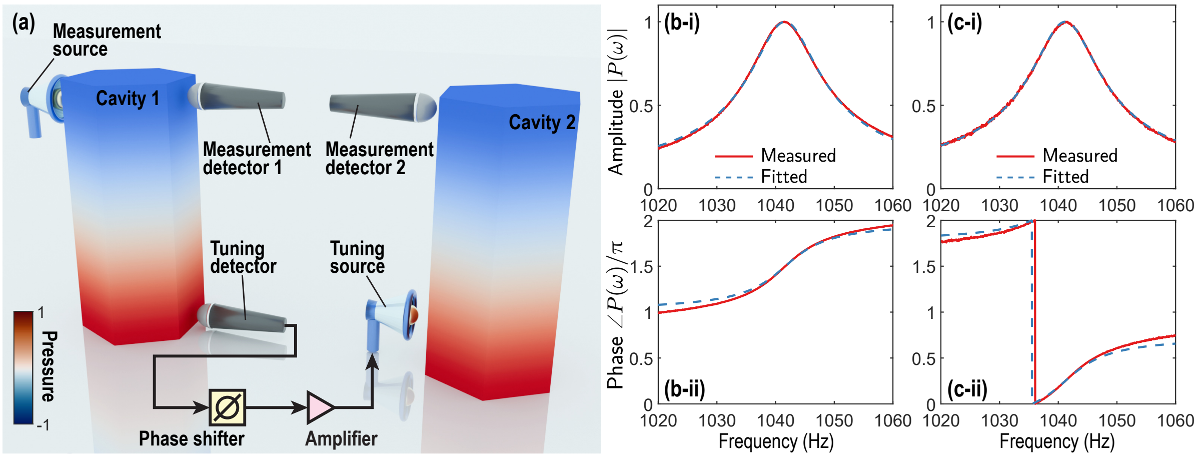

Figure S4: Tuning hoppings between two acoustic cavities. (a) Schematic illustration of the unidirectional hopping implmeneted using a detector and a source. The bottom microphone (tuning detector) in cavity 1 captures the acoustic pressure signal, which is processed by the controller to adjust its phase and amplitude before being emitted by the bottom loudspeaker (tuning source) in cavity 2. To determine both the amplitude and the phase of the unidirectional hopping κ_0, we excite at cavity 1 using a loudspeaker (measurement source) and measure the acoustic pressure in both cavities using two microphones (measurement detector). Experimentally measured and numerically fitted (**i**) amplitude and (**ii**) phase responses of the cross-power spectral density between the acoustic signals measured in cavities 1 and 2. For configuration (b), the target hopping strength is $\kappa_0 = t_0/2 = 1.5\,\mathrm{Hz}$, and the retrieved value through fitting experimental results is $\kappa_0 = 1.51\,\mathrm{Hz} + 0.01\mathrm{i}\,\mathrm{Hz}$. For configuration (c), the target hopping strength is $\kappa_0 = -h_y + \mathrm{i}h_x = -0.3\,\mathrm{Hz} + 0.3\mathrm{i}\,\mathrm{Hz}$, and the retrieved value through fitting experimental results is $\kappa_0 = -0.303\,\mathrm{Hz} + 0.302\mathrm{i}\,\mathrm{Hz}$.

As illustrated in Fig. S4a, each unidirectional hopping is realized through a loudspeaker, a microphone, and an audio amplifier. The loudspeaker and microphone are positioned at the bottom of each cavity. The microphone captures the acoustic pressure signal, which is processed by the controller to adjust its phase and amplitude before being emitted by the loudspeaker in the connected cavity. The analytical solution of this model is described in Sec. S2.4.

The tuning of the hopping parameters is performed as follows. As illustrated in Fig. S4a, a microphone is placed inside cavity 1 to detect the acoustic pressure. The detected pressure signal is then phase-adjusted, amplified, and emitted by the loudspeaker positioned in cavity 2. To determine both the amplitude and the phase of the unidirectional hopping κ_0 in Eq. (S11), we excite at cavity 1 using a loudspeaker (measurement source in Fig. S4a) and measure the acoustic pressure in both cavities using two microphones (measurement detector in Fig. S4). The cross-power spectral density of these two measured pressure signals is expressed by Eq. (S15).

S2.4 The coupled-model theory for the unidirectional coupling of two coupled acoustic cavities

Suppose two acoustic cavities (resonators) are connected by a unidirectional coupling κ_0. The tight-binding model Hamiltonian is

$$H_2 = \begin{pmatrix} \omega_0 & 0 \\ \kappa_0 & \omega_0 \end{pmatrix}. \tag{S11}$$

Here, $\mathrm{Re}(\omega_0)$ is the first resonant (angular) frequency of a single cavity, $-\mathrm{Im}(\omega_0) > 0$ is the intrinsic loss, and κ_0 is the unidirectional coupling realized by the active components. In general, $\kappa_0 \in \mathbb{C}$ is a complex number.

Based on the coupled-mode theory, the dynamic equation for this system with a source excitation $|s\rangle$ is

$$\mathrm{i}\frac{\mathrm{d}\,|\psi(t)\rangle}{\mathrm{d}t} = H\,|\psi(t)\rangle + |s(t)\rangle\,, \tag{S12}$$

where $|\psi(t)\rangle = \begin{bmatrix} \psi_1(t) & \psi_2(t) \end{bmatrix}^{\mathrm{T}}$ is the time-dependent sound pressure vector.

For a harmonic excitation at an angular frequency ω, the excitation and sound pressure vectors can be written as $|s(t)\rangle = |s\rangle\,\mathrm{e}^{-\mathrm{i}\omega t}$ and $|\psi(t)\rangle = |\psi\rangle\,\mathrm{e}^{-\mathrm{i}\omega t}$, respectively. The dynamic equation (S12) is then expressed as

$$(\omega - H_2)\,|\psi\rangle = |s\rangle\,. \tag{S13}$$

When the source is excited at cavity 1, the excitation vector is $|s\rangle = \psi_{\mathrm{in}}\begin{bmatrix} 1 & 0 \end{bmatrix}^{\mathrm{T}}$, where ψ_{in} is the complex amplitude. Consequently, the sound pressure distribution is obtained by solving Eq. (S13) to give

$$|\psi(t)\rangle = \psi_{\mathrm{in}}\mathrm{e}^{-\mathrm{i}\omega t}\begin{bmatrix} \dfrac{1}{\omega - \omega_0} & \dfrac{\kappa_0}{(\omega - \omega_0)^2} \end{bmatrix}^{\mathrm{T}}. \tag{S14}$$

To determine the phase of the unidirectional coupling κ_0, the cross-power spectral density between two measured signals is calculated in experiments

$$P(\omega) \equiv \frac{\psi_2(\omega)}{\psi_1(\omega)} = \frac{\kappa_0}{\omega - \omega_0}. \tag{S15}$$

It is observed that, when $\omega = \mathrm{Re}(\omega_0)$, the phase of P and κ_0 follows the relation

$$\angle P(\mathrm{Re}(\omega_0)) = \angle\kappa_0 - \frac{\pi}{2}. \tag{S16}$$

S2.5 Statistics and long-term stability of onsite potential and hopping tuning

We conducted statistics and long-term stability tests for both onsite potential tuning and hopping tuning. We first tune the onsite potentials and hopping strengths to the target values, and then measure them every 15 minutes over a period of 6 hours to evaluate their long-term stability. The results for the onsite potential tuning and hopping tuning are shown in Fig. S5 and Fig. S6, respectively, for the case of $h_x = 1.8\,\text{Hz}\,(h_y = h_z = 0)$. The results for the onsite potential tuning and hopping tuning are shown in Fig. S7 and Fig. S8, respectively, for the case of $h_y = 1.8\,\text{Hz}\,(h_x = h_z = 0)$. The results for the onsite potential tuning and hopping tuning are shown in Fig. S9 and Fig. S10, respectively, for the case of $h_z = 1.8\,\text{Hz}\,(h_x = h_y = 0)$. These three cases are representative configurations showing NHDS and D-NHSE. The corresponding energy spectra and eigenstates are presented in Fig. 3 of the main text.

Taking the case with $h_x = 1.8\,\text{Hz}$ as an example, as shown in Fig. S5(a), the onsite potentials of two representative cavities are measured over a period of 6 hours, showing minimal drift ($< 0.2\,\text{Hz}$). The mean and standard deviation of the measured onsite potentials for all 56 cavities are shown in Figs. S5(b) and (c), respectively. These results indicate that the onsite potentials are uniformly tuned across the entire lattice, with standard deviations less than $0.22\,\text{Hz}$ for both real and imaginary parts. Figures S5(d–f) show the measured onsite potentials for all 56 cavities in the complex plane at time 0 hour, 3 hours, and 6 hours, respectively. It is noted that the original onsite potentials before tuning (black dots) are significantly different from the target values (dashed lines), while the tuned onsite potentials (colored dots) are very close to the target values and remain stable over a long time (several hours).

As shown in Fig. S6(a), the hopping strengths for five representative hoppings are measured over a period of 6 hours, also showing minimal drift ($< 0.1\,\text{Hz}$). The mean and standard deviation of the measured hopping strengths for all hoppings in the lattice consisting of 56 cavities are shown in Figs. S6(b) and (c), respectively. These results indicate that the hopping strengths are uniformly tuned across the entire lattice, with standard deviations less than $0.06\,\text{Hz}$ for both real and imaginary parts. Figures S6(d–f) show the measured hopping strengths in the complex plane at time 0 hour, 3 hours, and 6 hours, respectively. It is seen that the tuned hopping strengths (colored dots) are very close to the target values (dashed lines) and remain stable over time.

The results from Figs. S5–S10 demonstrate that both the onsite potentials and hopping strengths can be precisely regulated to the target values with high stability over a period of 6 hours. Such long-term stability and precision are enough to ensure the reliability and reproducibility of our experimental observations of NHDS and D-NHSE.

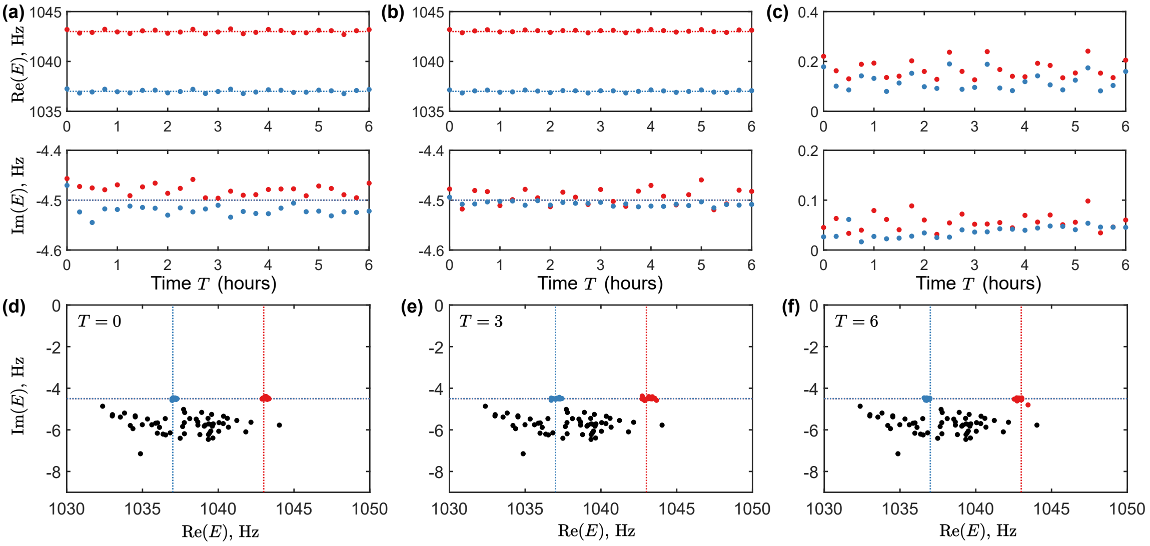

Figure S5: Statistics and long-term stability of onsite potential tuning. (a) Real (top) and imaginary (bottom) parts of the onsite potentials of two representative cavities (first unit cell) measured over a time period (T) of 6 hours. (b) Mean and (c) standard deviation of the real and imaginary parts of the measured onsite potentials for all 56 cavities. Results are shown on sublattice 'a' (●) and sublattice 'b' (●). Measured onsite potentials for 56 cavities shown in the complex plane at time (d) $T = 0$ hour, (e) $T = 3$ hours, and (f) $T = 6$ hours. The values before tuning are shown as ● . Dashed lines indicate the target onsite potentials $1043\,\text{Hz} - 4.5\text{i}\,\text{Hz}$ (red) and $1037\,\text{Hz} - 4.5\text{i}\,\text{Hz}$ (blue). The rest of the parameters are $\omega_0 = 1040\,\text{Hz} - 4.5\text{i}\,\text{Hz}$, $t_0 = -m_0 = 3\,\text{Hz}$, $h_x = 1.8\,\text{Hz}$, and $h_y = h_z = 0$.

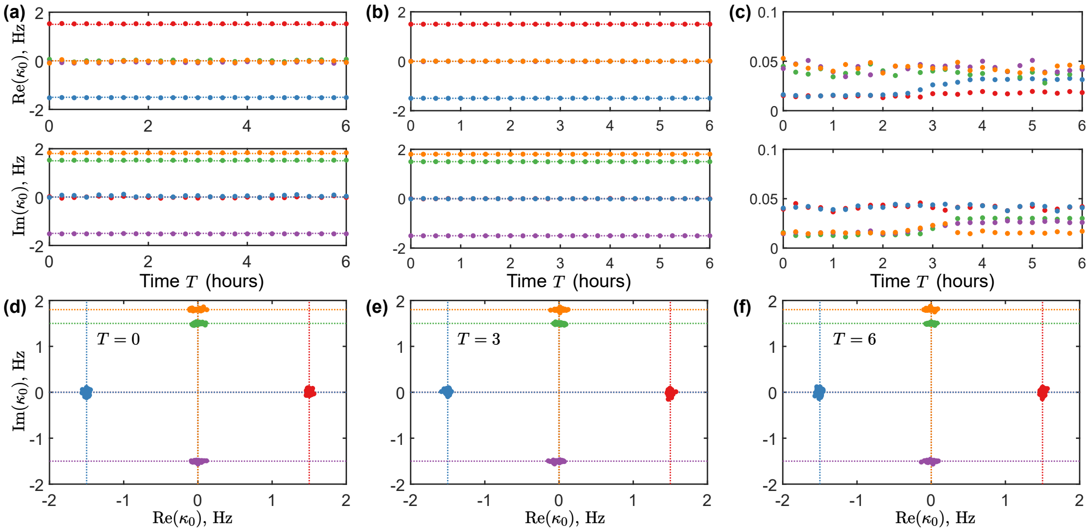

Figure S6: Statistics and long-term stability of hopping tuning. (a) Real (top) and imaginary (bottom) parts of the measured hopping strengths for five representative hoppings over a time period (T) of 6 hours. (b) Mean and (c) standard deviation of the measured hopping strengths for all hoppings in the lattice consisting of 56 cavities. Measured hopping strengths shown in the complex plane at time (d) $T = 0$ hour, (e) $T = 3$ hours, and (f) $T = 6$ hours. Dashed lines indicate the target hopping strengths $t_0/2 = 1.5\,\text{Hz}$ (red), $-t_0/2 = -1.5\,\text{Hz}$ (blue), $\text{i}t_0/2 = 1.5\text{i}\,\text{Hz}$ (green), $-\text{i}t_0/2 = -1.5\text{i}\,\text{Hz}$ (purple), and $\text{i}h_x = 1.8\text{i}\,\text{Hz}$ (orange). Colored dots are the corresponding measured hopping strengths. The rest of the parameters are $\omega_0 = 1040\,\text{Hz} - 4.5\text{i}\,\text{Hz}$, $t_0 = -m_0 = 3\,\text{Hz}$, $h_x = 1.8\,\text{Hz}$, and $h_y = h_z = 0$.

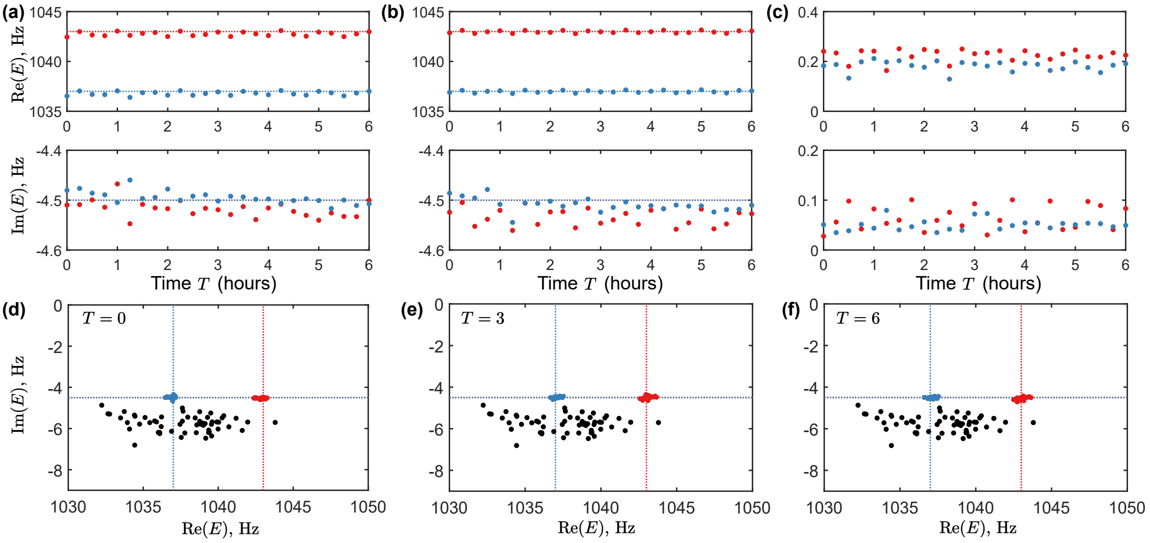

Figure S7: Statistics and long-term stability of onsite potential tuning. (a) Real (top) and imaginary (bottom) parts of the onsite potentials of two representative cavities (first unit cell) measured over a period of 6 hours. (b) Mean and (c) standard deviation of the real and imaginary parts of the measured onsite potentials for all 56 cavities. The results are shown on sublattice ‘a’ (●) and sublattice ‘b’ (●). Measured onsite potentials for 56 cavities shown in the complex plane at time (d) $T = 0$ hour, (e) $T = 3$ hours, and (f) $T = 6$ hours. The values before tuning are shown as ● . Dashed lines indicate the target onsite potentials are $1043\,\text{Hz} - 4.5\text{i}\,\text{Hz}$ (red) and $1037\,\text{Hz} - 4.5\text{i}\,\text{Hz}$ (blue). The rest of the parameters are $\omega_0 = 1040\,\text{Hz} - 4.5\text{i}\,\text{Hz}$, $t_0 = -m_0 = 3\,\text{Hz}$, $h_y = 1.8\,\text{Hz}$, and $h_x = h_z = 0$.

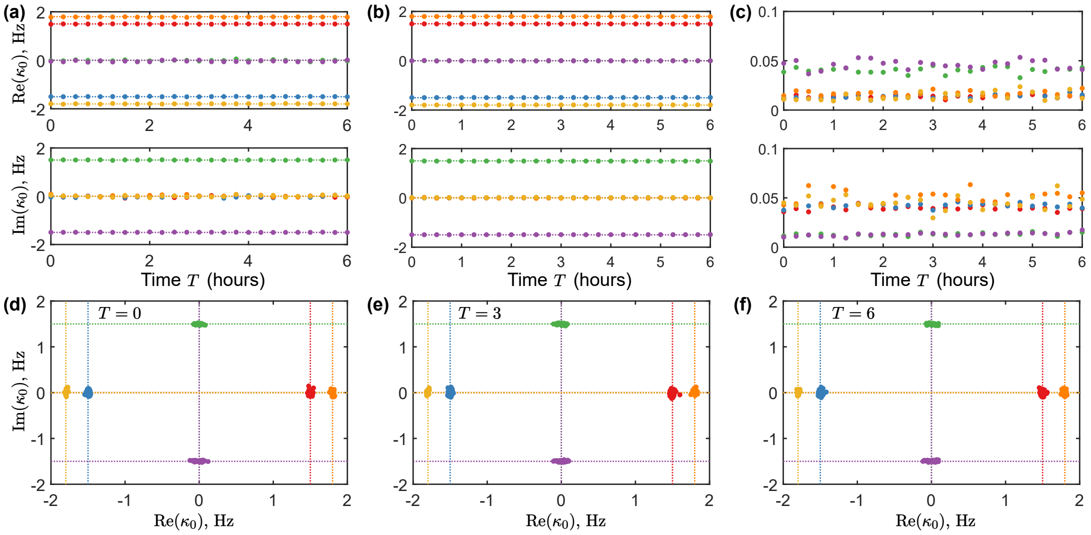

Figure S8: Statistics and long-term stability of hopping tuning. (a) Real (top) and imaginary (bottom) parts of the measured hopping strengths for six representative hoppings over a period of 6 hours. (b) Mean and (c) standard deviation of the measured hopping strengths for all hoppings in the lattice consisting of 56 cavities. Measured hopping strengths shown in the complex plane at time (d) $T = 0$ hour, (e) $T = 3$ hours, and (f) $T = 6$ hours. Dashed lines indicate the target hopping strengths $t_0/2 = 1.5\,\text{Hz}$ (red), $-t_0/2 = -1.5\,\text{Hz}$ (blue), $\text{i}t_0/2 = 1.5\text{i}\,\text{Hz}$ (green), $-\text{i}t_0/2 = -1.5\text{i}\,\text{Hz}$ (purple), $h_y = 1.8\,\text{Hz}$ (orange), and $-h_y = -1.8\,\text{Hz}$ (yellow). Colored dots are the corresponding measured hopping strengths. The rest of the parameters are $\omega_0 = 1040\,\text{Hz} - 4.5\text{i}\,\text{Hz}$, $t_0 = -m_0 = 3\,\text{Hz}$, $h_y = 1.8\,\text{Hz}$, and $h_x = h_z = 0$.

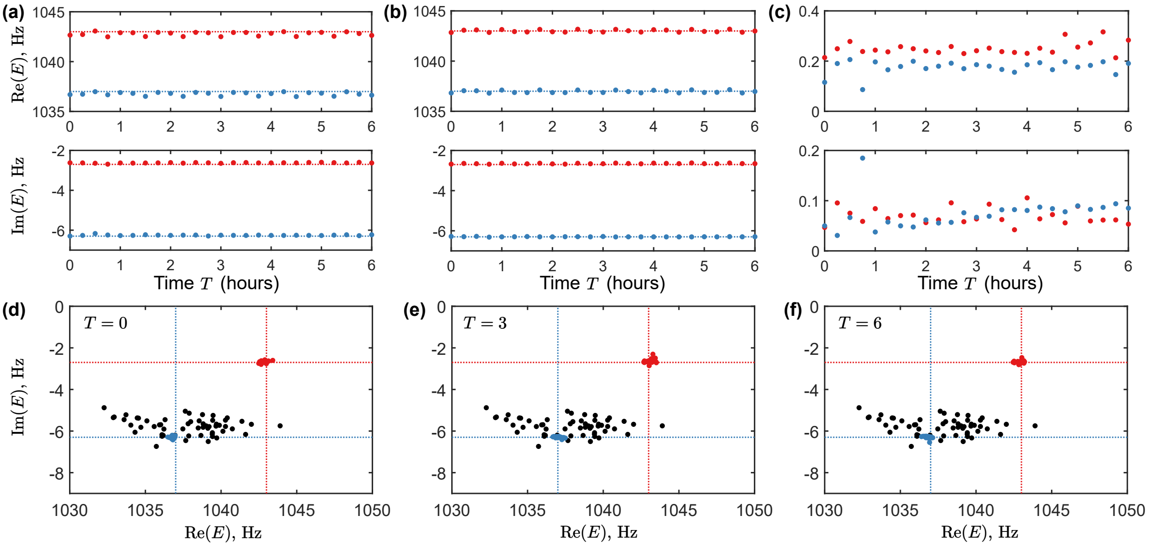

Figure S9: Statistics and long-term stability of onsite potential tuning. (a) Real (top) and imaginary (bottom) parts of the onsite potentials of two representative cavities (first unit cell) measured over a period of 6 hours. (b) Mean and (c) standard deviation of the real and imaginary parts of the measured onsite potentials for all 56 cavities. The results are shown on sublattice 'a' (●) and sublattice 'b' (●). Measured onsite potentials for 56 cavities shown in the complex plane at time (d) $T = 0$ hour, (e) $T = 3$ hours, and (f) $T = 6$ hours. The values before tuning are shown as ● . Dashed lines indicate the target onsite potentials $1043\,\text{Hz} - 2.7\text{i}\,\text{Hz}$ (red) and $1037\,\text{Hz} - 6.3\text{i}\,\text{Hz}$ (blue). The rest of the parameters are $\omega_0 = 1040\,\text{Hz} - 4.5\text{i}\,\text{Hz}$, $t_0 = -m_0 = 3\,\text{Hz}$, $h_z = 1.8\,\text{Hz}$, and $h_x = h_y = 0$.

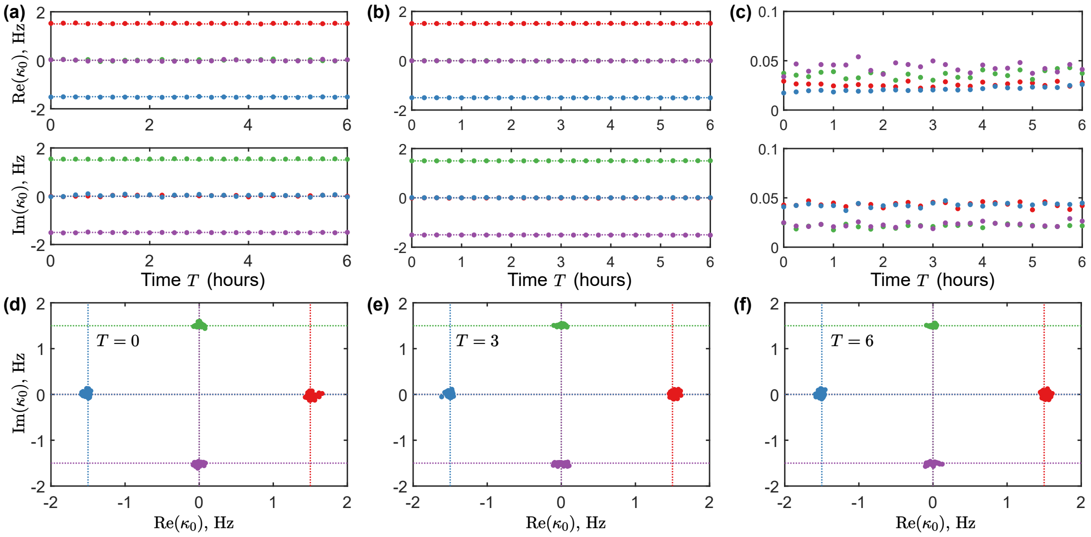

Figure S10: Statistics and long-term stability of hopping tuning. (a) Real (top) and imaginary (bottom) parts of the measured hopping strengths for four representative hoppings over a period of 6 hours. (b) Mean and (c) standard deviation of the measured hopping strengths for all hoppings in the lattice consisting of 56 cavities. Measured hopping strengths shown in the complex plane at time (d) $T = 0$ hour, (e) $T = 3$ hours, and (f) $T = 6$ hours. Dashed lines indicate the target hopping strengths $t_0/2 = 1.5\,\text{Hz}$ (red), $-t_0/2 = -1.5\,\text{Hz}$ (blue), $\text{i}t_0/2 = 1.5\text{i}\,\text{Hz}$ (green), and $-\text{i}t_0/2 = -1.5\text{i}\,\text{Hz}$ (purple). Colored dots are the corresponding measured hopping strengths. The rest of the parameters are $\omega_0 = 1040\,\text{Hz} - 4.5\text{i}\,\text{Hz}$, $t_0 = -m_0 = 3\,\text{Hz}$, $h_z = 1.8\,\text{Hz}$, and $h_x = h_y = 0$.

S2.6 Audio amplifiers and self-oscillation issues

The audio power amplifier used in all coupling channels is the Texas Instruments LM386. According to the datasheet, its total harmonic distortion in our operating range is below 0.2%, and its intrinsic noise floor is much lower than the acoustic background and microphone noise in our setup. In the present implementation, the gain of each LM386 stage is set to unity; the effective loop gain and sensitivity of each coupling channel are therefore governed by the product of microphone sensitivity, amplifier gain, loudspeaker efficiency, and the complex-valued feedback coefficients (gain and phase) implemented in the controller.

Self-oscillation occurs when the total feedback gain drives at least one eigenstate of the non-Hermitian lattice into the unstable regime. In spectral terms, this means that there exists at least one eigenenergy with a positive imaginary part in the complex spectrum [2]. The temporal evolution of the corresponding state is proportional to $\exp[\mathrm{Im}(E_n)t]$, so any eigenenergy E_n with $\mathrm{Im}(E_n) > 0$ will grow exponentially in time. Even in the absence of an external pump source, background noise can then excite this mode and lead to self-oscillation. Because we can experimentally reconstruct the full complex spectrum via the Green's-function-based method [3], we directly check the lattice whether any eigenenergy has a positive imaginary part. In all configurations reported in this work, the measured spectra show that all eigenenergies have negative imaginary parts, confirming that the system is stable and does not self-oscillate.

S2.7 Implementation of PBCs and OBCs

In our experiments, all hoppings are impemented through microphone-loudspeaker pairs. As they are connected by electrical circuits, we can easily connect the last site to the first site to realize PBCs, or leave them unconnected to realize OBCs. To better illustrate this implementation, we use a simple 1D chain with four sites as an example, as shown in Fig. S11. To realize PBCs, we connect site 1 and site 4 through a microphone-loudspeaker pair (indicated by the dashed line in Fig. S11). To realize OBCs, we simply remove this hopping by disconnecting the microphone-loudspeaker pair between site 1 and site 4. This implementation method can be easily extended to our 2D acoustic NH Chern lattice.

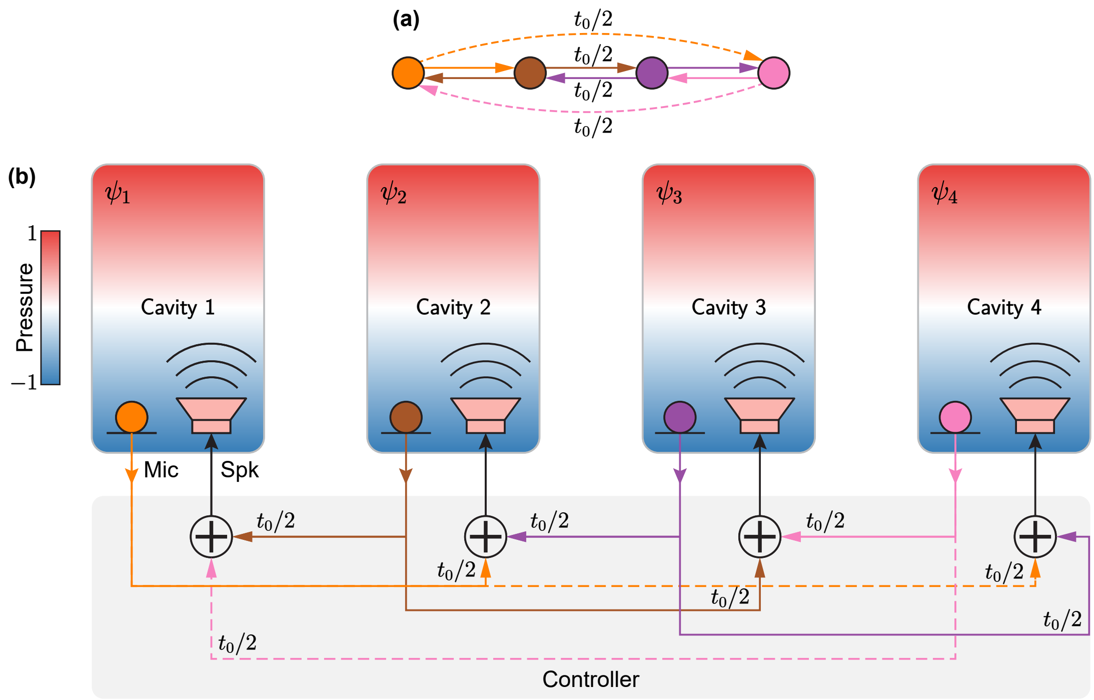

Figure S11: A schematic implementation of PBCs and OBCs in experiments. (a) Sketch of the tight-binding model of a simple 1D chain with $N = 4$ sites. The corresponding Bloch Hamiltonian is given by $H = t_0 \cos k$ with t_0 being the hopping strength and k being the momentum. (b) A schematic illustration of implementing PBCs and OBCs in our experimental setup. Each hopping is realized through a microphone-loudspeaker (Mic-Spk) pair connected by an electrical circuit (controller). In both panels, the chain is under OBCs (PBCs) when the hoppings between site 1 and site 4 (indicated by dashed lines) are removed (included).

S2.8 Validation of complex spectrum and eigenstate extraction via passive acoustic tubes

The method of measuring the complex energy spectrum and eigenstates via Green's functions is quite general and can be applied to a wide range of non-Hermitian acoustic systems, including those with coupling tubes. The key reason is that this method is based on the fundamental relationship between the Green's function and the system's Hamiltonian, which holds regardless of the specific implementation of couplings. In fact, this method is not limited to acoustic systems; it can also be applied to other wave systems such as photonic, mechanical, elastic, and microwave systems, as long as the Green's function can be measured.

The challenge of this method lies in accurately measuring the Green's function, for both amplitude and phase responses. In most works, only the amplitude response is measured, which is insufficient for reconstructing the full complex spectrum and eigenstates. While in our work, we additionally obtain the accurate phase response by taking the excitation signal as the reference. Another challenge is the measurement of the full Green's function matrix, which requires exciting and detecting signals at all possible pairs of sites in the lattice. In most previous works, the responses are measured with the source excited at only one site, which requires N measurements for a lattice size of N. However, in order to reconstruct the full Green's function matrix, we need to excite and detect signals at all possible pairs of sites resulting in N^2 measurements, which can be time-consuming for two-dimensional lattices investigated in this work. We overcome this challenge by programming a data acquisition (DAQ) system (National Instruments PCIe-6353) to automatically perform all measurements.

When we obtain the full Green's function matrix $G(\omega)$ at frequency ω, we can reconstruct the complex energy spectrum and eigenstates using the same procedure as described in our work [3]. Specifically, the Green's function is directly related to the lattice Hamiltonian or non-Hermitian operator H via the relation

$$G(\omega) = (\omega - H)^{-1}. \tag{S17}$$

Using the spectral decomposition, $H = \sum_n E_n \left|\psi_n^{\mathrm{R}}\right\rangle \left\langle \psi_n^{\mathrm{L}}\right|$, the Green's function can be expressed as

$$G(\omega) = \sum_n \frac{1}{\omega - E_n} \left|\psi_n^{\mathrm{R}}\right\rangle \left\langle \psi_n^{\mathrm{L}}\right|. \tag{S18}$$

Here, n is the state index, E_n is the complex eigenenergy, and $\left|\psi_n^{\mathrm{R}}\right\rangle$ and $\left\langle \psi_n^{\mathrm{L}}\right|$ are the corresponding right and left eigenstates, respectively. When $G(\omega)$ acts on the right eigenstate $\left|\psi_n^{\mathrm{R}}\right\rangle$ from the left or on the left eigenstate $\left\langle \psi_n^{\mathrm{L}}\right|$ from the right, we have

$$G(\omega)\left|\psi_n^{\mathrm{R}}\right\rangle = \frac{1}{\omega - E_n}\left|\psi_n^{\mathrm{R}}\right\rangle, \quad \left\langle \psi_n^{\mathrm{L}}\right| G(\omega) = \frac{1}{\omega - E_n}\left\langle \psi_n^{\mathrm{L}}\right|. \tag{S19}$$

Equation (S19) demonstrates that the right (left) eigenstate of the Hamiltonian H is also the right (left) eigenstate of the Green's function $G(\omega)$, with the corresponding eigenvalue being $1/(\omega - E_n)$. Therefore, by diagonalizing the measured Green's function matrix $G(\omega)$ at each frequency ω, we can obtain N well-defined single-peak curves that correspond to the magnitude of $1/(\omega - E_n)$. By fitting each curve with this simple expression, we can precisely determine both the real and imaginary parts of the complex eigenenergy E_n, thereby obtaining the complete complex-valued energy spectrum. In addition to the spectrum, the left and right eigenstates can also be directly extracted as the eigenstates of $G(\omega)$.

To further illustrate the general applicability of this method, we consider an minimal model of two coupled acoustic cavities with coupling tubes, as shown in Fig. S12(a–b). This system can be described by the following Hamiltonian:

$$H = \begin{bmatrix} \omega_1 & \kappa \\ \kappa & \omega_2 \end{bmatrix}, \tag{S20}$$

where κ is the reciprocal coupling strength provided by the coupling tube. Here we use ω_1 and ω_2 to denote the onsite potentials of the two cavities, with real and imaginary parts representing the resonant frequency and intrinsic loss, respectively. Note that, in general, $\omega_1 \neq \omega_2$ due to fabrication imperfections. Before connecting the two cavities, we can measure their individual frequency response and fit them with the expression $1/(\omega - \omega_j)$ $(j = 1,2)$ to extract the onsite potentials $\omega_1 = 1039.58\,\mathrm{Hz} - 6.07\mathrm{i}\,\mathrm{Hz}$ and $\omega_2 = 1042.94\,\mathrm{Hz} - 4.73\mathrm{i}\,\mathrm{Hz}$. After connecting the two cavities via the coupling tube, we use the same method described in Sec. S3 of the SM of Wu's work [4] to determine the reciprocal hopping strength $\kappa = 112.1\,\mathrm{Hz}$. By substituting these parameters into Eq. (S20), we can calculate the complex eigenenergies of the coupled system as $E_1^{\mathrm{pred}} = 929.12\,\mathrm{Hz} - 5.41\mathrm{i}\,\mathrm{Hz}$ and $E_2^{\mathrm{pred}} = 1153.41\,\mathrm{Hz} - 5.39\mathrm{i}\,\mathrm{Hz}$. These predicted eigenenergies can be directly compared with the experimental results obtained via the Green's function method.

We then use the Green's-function-based method [3] to measure this coupled system. The measured Green's function amplitude and phase are shown in Fig. S12(c). By diagonalizing the measured Green's function matrix at each frequency ω, we obtain eigenvalues forming two well-defined single-peak curves, as shown in Fig. S12(d). By fitting these curves with the expression $1/(\omega - E_n)$, we can extract the complex eigenenergies as $E_1^{\mathrm{exp}} = 922.91\,\mathrm{Hz} - 4.81\mathrm{i}\,\mathrm{Hz}$ and $E_2^{\mathrm{exp}} = 1158.42\,\mathrm{Hz} - 5.94\mathrm{i}\,\mathrm{Hz}$. These values align well with the predicted eigenenergies, as shown in Fig. S12(e). Furthermore, the corresponding eigenstates can also be directly obtained as the eigenstates of the measured Green's function. The predicted and experimentally measured eigenstates are shown in Fig. S12(f), demonstrating good agreement. This example illustrates that the Green's-function-based method can be effectively applied to measure the complex energy spectrum and eigenstates in acoustic systems with coupling tubes.

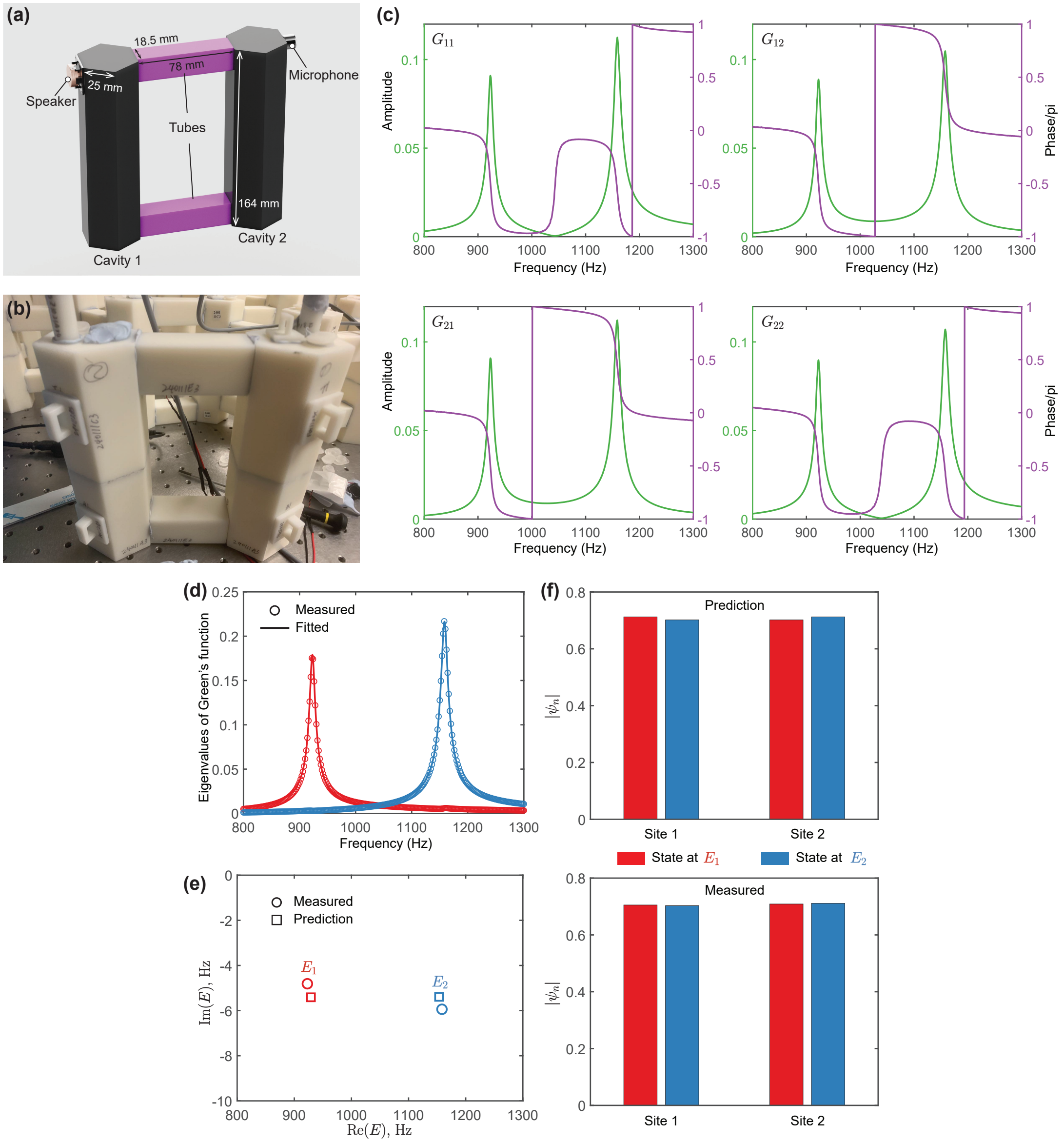

Figure S12: Measurement of complex energy spectrum using the Green's-function-based approach for two coupled acoustic cavities connected via a coupling tube. (a) Schematic of two coupled acoustic cavities connected via a coupling tube. Only the airborne domain is shown for clarity. (b) Photo of the experimental setup. (c) Measured Green's function amplitude (left axis) and phase (right axis). Here, G_{ij} represents the Green's function measured with the source excited at cavity j and the response detected at cavity i. (d) Eigenvalues (circles) of measured Green's function, with solid lines representing fitted curves. (e) Experimentally measured eigenenergies (circles, E_1 and E_2) and the predicted eigenenergies (squares). (f) Predicted (top row) and experimentally measured (bottom row) eigenstates corresponding to the two eigenenergies.

S3 Measured Green's function responses and reconstruction of complex spectrum and eigenstates

In Figs. S13(b,c), we present the measured Green's function amplitude at all sites with the source excited at two different sites, i.e., site 1 and site 14, respectively. The site index is illustrated in Fig. S13(a).

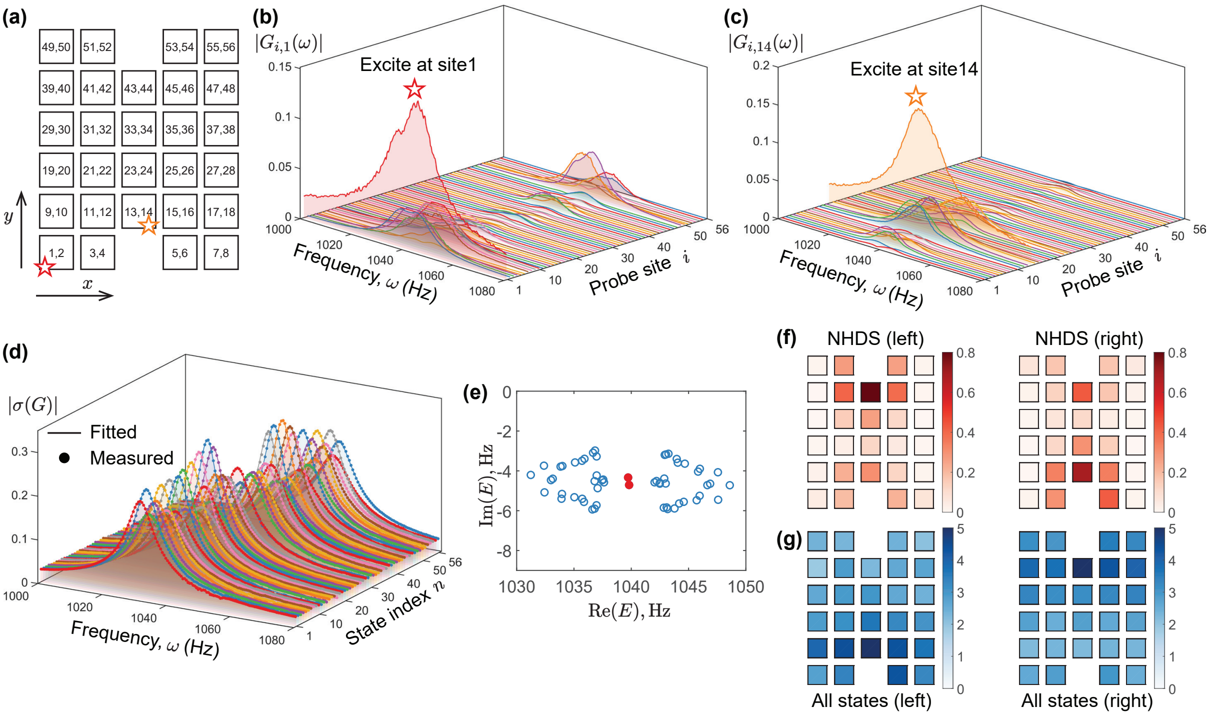

Figure S13: Experimental results of NH acoustic Chern insulators with an edge dislocation-antidislocation pair under PBCs in the M **phase.** (a) Sketch of the site index in the lattice. The first and second numbers inside the square represent the index of the sublattice 'a' and 'b' within a unit cell, respectively. Measured Green's function amplitude at all sites with the source excited at site (b) 1 and (c) 14. (d) Amplitude of the eigenvalues of the (dottes) measured Green's function at each frequency. Solid lines indicate the fitted curves of the eigenvalues using the expression $1/(\omega - E_n)$. (e) Energy spectrum reconstructed from the measured Green's function, where the red dots (●) correspond to NHDS and Blue circles (○) represent the other states. (f) Left and right eigenstates of the NHDS reconstructed from the measured Green's function. (g) Summed amplitude distributions of all left and right eigenstates. The rest of the parameters are chosen to be $\omega_0 = 1040\,\text{Hz} - 4.5\text{i}\,\text{Hz}, t_0 = -m_0 = 3\,\text{Hz}, h_y = 1.8\,\text{Hz}, h_x = h_z = 0$.

From Figs. S13(b,c), we can see that the response amplitude is significantly larger at the cavities near the source site, and gradually decreases as the distance from the source site increases. This is hard to observe the signature of the NHDS nor the D-NHSE directly from these Green's function responses. The reason is that the overall loss in the system leads to a rapid decay of the response amplitude when the acoustic wave propagates away from the source site [5]. In other words, the imaginary part of the eigenenergies of all states is negative and small, as seen from Fig. S13(e). Therefore, observing the measured spectral response is insufficient to identify the NHDS or the D-NHSE. Our Green's-function-based approach is essential to address this challenge.

As shown in Fig. S13(d), by diagonalizing the measured Green's function matrix at each frequency ω, we obtain eigenvalues forming well-defined single-peak curves. By fitting these curves with the expression $1/(\omega - E_n)$, we can extract the complex eigenenergies, thereby obtaining the complete complex-valued energy spectrum, as shown in Fig. S13(e). In addition to the spectrum, the left and right eigenstates can also be directly extracted as the eigenstates of the measured Green's function. The NHDS can be clearly identified from the reconstructed energy spectrum and eigenstates, with the amplitude distribution shown in Fig. S13(f). Moreover, by summing the amplitude distributions of all left and right eigenstates, we can directly observe the D-NHSE, as shown in Fig. S13(g).

S4 Characterizing bulk EPs in a pristine 4×4 lattice

Figures S14–S16 show the experimental observation of bulk EPs in a pristine 4×4 acoustic Chern lattice without dislocations under PBCs with NH perturbations for $t_0 = -m_0 = 3\,\text{Hz}$. For comparison, we also include theoretical calculations that account for disorder. Specifically, we modify the real-space Hamiltonian H_{real} by adding a random disorder term H_{dis}, whose entries are complex random variables uniformly distributed in $[-W, W]$ with $W = 0.1\,\text{Hz}$. The experimental results are found to yield a better agreement with the theoretical prediction when accounting for disorders in the system.

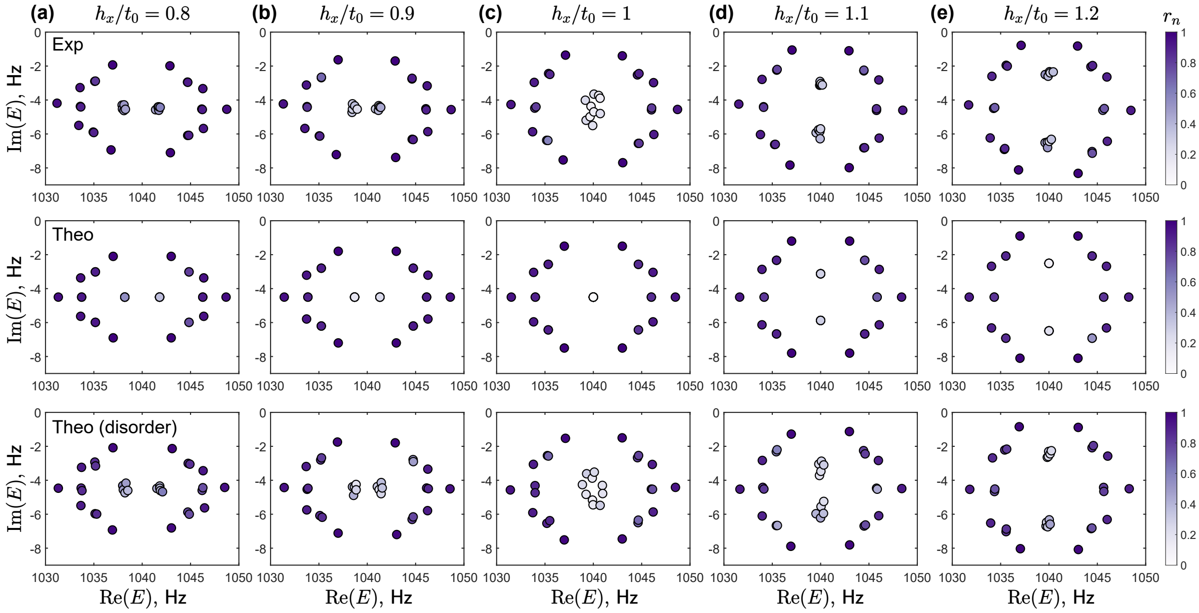

Figure S14: Experimental observation of bulk EPs in a pristine 4×4 acoustic Chern lattice without dislocations under PBCs with NH perturbations for $t_0 = -m_0 = 3\,\text{Hz}$. The phase rigidity, r_n, of the nth state is shown by color. The spectra are shown for varying NH perturbation strengths: (a) $h_x/t_0 = 0.8$, (b) $h_x/t_0 = 0.9$, (c) $h_x/t_0 = 1.0$, (d) $h_x/t_0 = 1.1$, and (e) $h_x/t_0 = 1.2$. In each subfigure, the panels from top to bottom show the experimental results, the theoretical prediction for the clean (disorder-free) case, and the theoretical calculation including disorder, respectively.

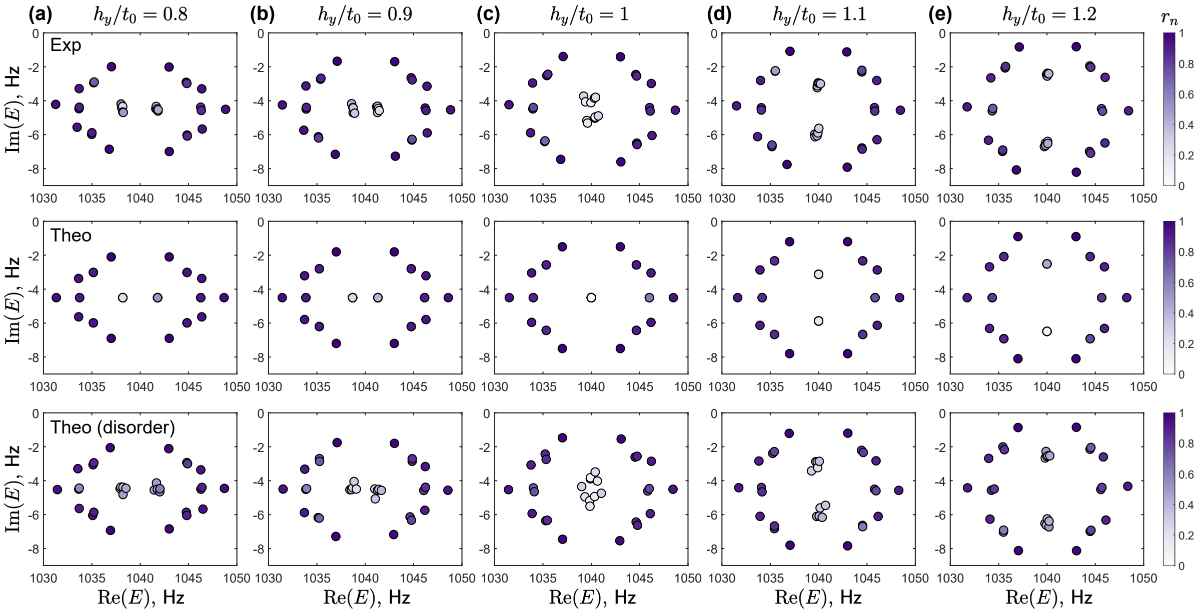

Figure S15: Experimental observation of bulk EPs in a pristine 4×4 acoustic Chern lattice without dislocations under PBCs with NH perturbations for $t_0 = -m_0 = 3\,\text{Hz}$. The phase rigidity, r_n, of the nth state is shown by color. The spectra are shown for varying NH perturbation strengths: (a) $h_y/t_0 = 0.8$, (b) $h_y/t_0 = 0.9$, (c) $h_y/t_0 = 1.0$, (d) $h_y/t_0 = 1.1$, and (e) $h_y/t_0 = 1.2$. In each subfigure, the panels from top to bottom show the experimental results, the theoretical prediction for the clean (disorder-free) case, and the theoretical calculation including disorder, respectively.

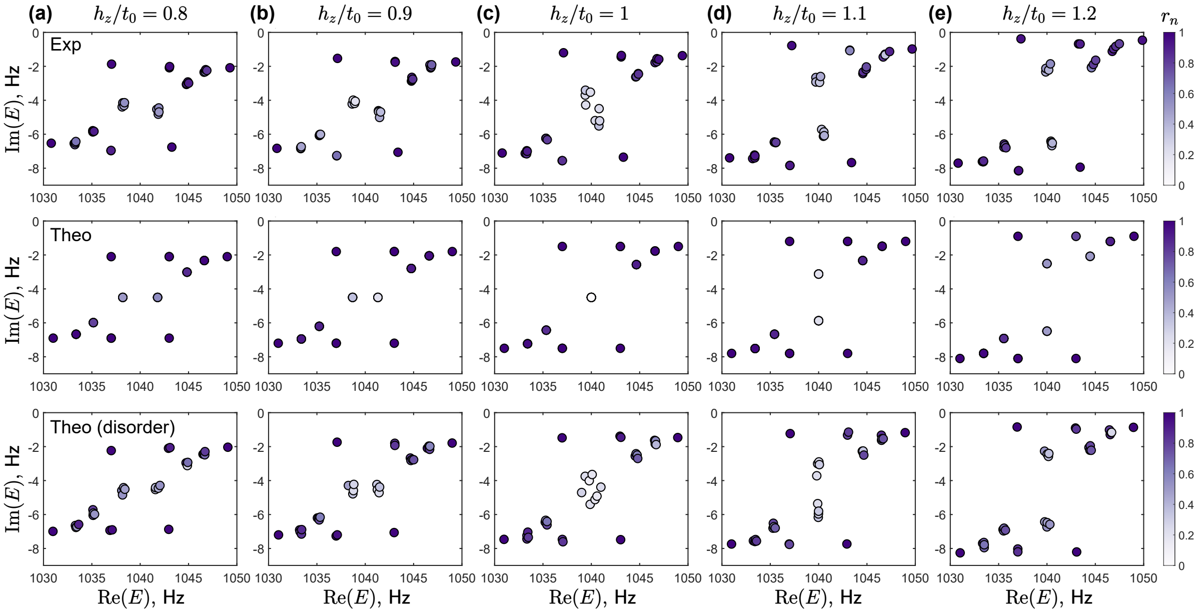

Figure S16: Experimental observation of bulk EPs in a pristine 4×4 acoustic Chern lattice without dislocations under PBCs with NH perturbations for $t_0 = -m_0 = 3\,\text{Hz}$. The phase rigidity, r_n, of the nth state is shown by color. The spectra are shown for varying NH perturbation strengths: (a) $h_z/t_0 = 0.8$, (b) $h_z/t_0 = 0.9$, (c) $h_z/t_0 = 1.0$, (d) $h_z/t_0 = 1.1$, and (e) $h_z/t_0 = 1.2$. In each subfigure, the panels from top to bottom show the experimental results, the theoretical prediction for the clean (disorder-free) case, and the theoretical calculation including disorder, respectively.

S5 Phase rigidity analysis near bulk EPs for the 5×6 lattice with dislocation

Figures S17–S19 show the experimental observation of spectra in a 5×6 acoustic Chern lattice in the presence of an edge dislocation-antidislocation pair under PBCs with NH perturbations for $t_0 = -m_0 = 3\,\text{Hz}$. For comparison, we also include theoretical calculations that account for disorder. Specifically, we modify the real-space Hamiltonian H_{real} by adding a random disorder term H_{dis}, whose entries are complex random variables uniformly distributed in $[-W, W]$ with $W = 0.1\,\text{Hz}$.

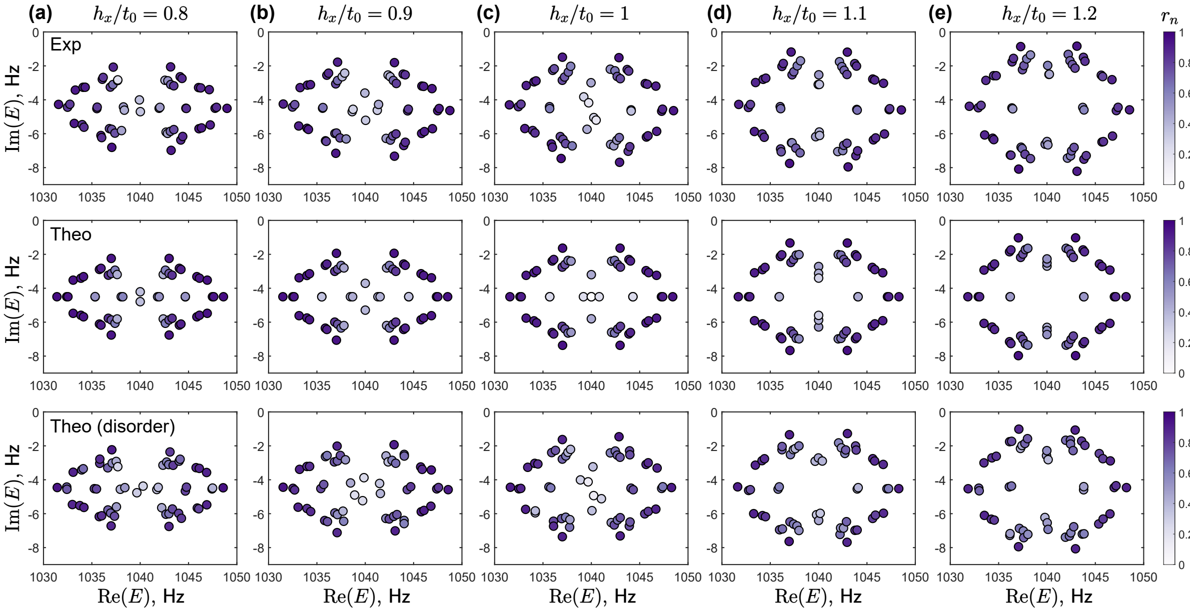

Figure S17: Experimental observation of a 5×6 acoustic Chern lattice in the presence of an edge dislocation-antidislocation pair under PBCs with NH perturbations for $t_0 = -m_0 = 3\,\text{Hz}$. The phase rigidity, r_n, of the nth state is shown by color. The spectra are shown for varying NH perturbation strengths: (a) $h_x/t_0 = 0.8$, (b) $h_x/t_0 = 0.9$, (c) $h_x/t_0 = 1.0$, (d) $h_x/t_0 = 1.1$, and (e) $h_x/t_0 = 1.2$. In each subfigure, the panels from top to bottom show the experimental results, the theoretical prediction for the clean (disorder-free) case, and the theoretical calculation including disorder, respectively.

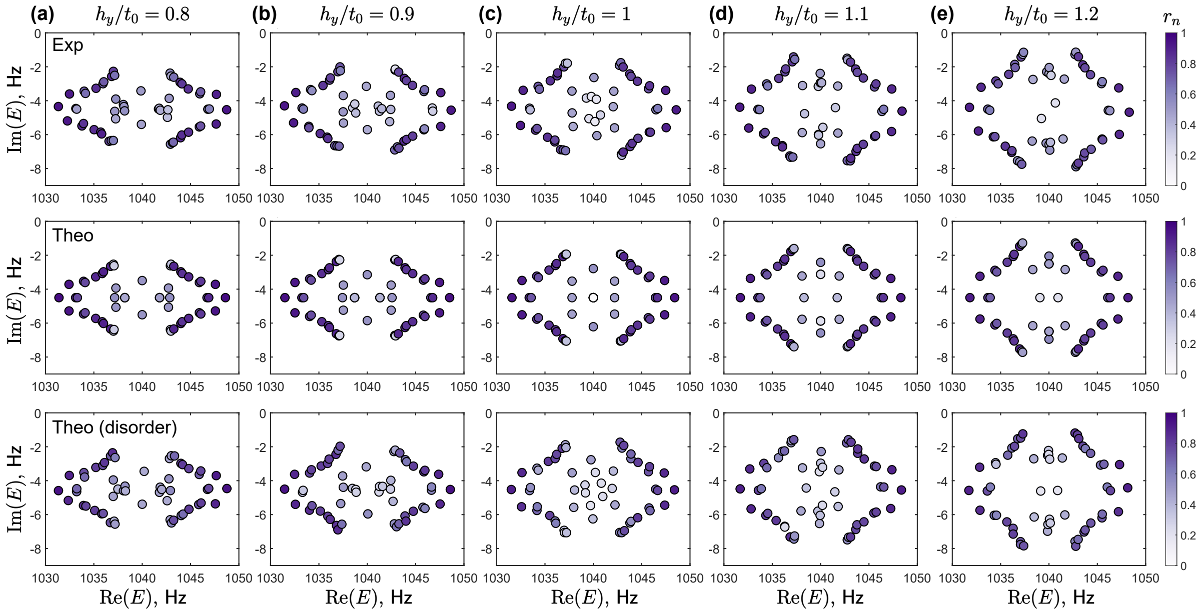

Figure S18: Experimental observation of a 5×6 acoustic Chern lattice in the presence of an edge dislocation-antidislocation pair under PBCs with NH perturbations for $t_0 = -m_0 = 3\,\text{Hz}$. The phase rigidity, r_n, of the nth state is shown by color. The spectra are shown for varying NH perturbation strengths: (a) $h_y/t_0 = 0.8$, (b) $h_y/t_0 = 0.9$, (c) $h_y/t_0 = 1.0$, (d) $h_y/t_0 = 1.1$, and (e) $h_y/t_0 = 1.2$. In each subfigure, the panels from top to bottom show the experimental results, the theoretical prediction for the clean (disorder-free) case, and the theoretical calculation including disorder, respectively.

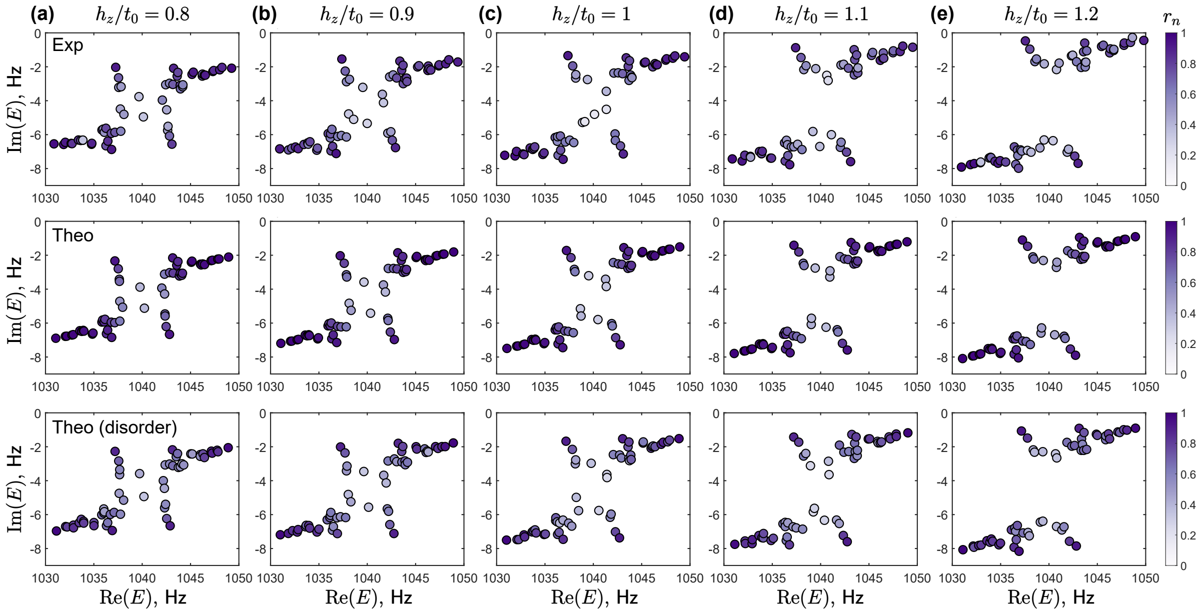

Figure S19: Experimental observation of a 5×6 acoustic Chern lattice in the presence of an edge dislocation-antidislocation pair under PBCs with NH perturbations for $t_0 = -m_0 = 3\,\text{Hz}$. The phase rigidity, r_n, of the nth state is shown by color. The spectra are shown for varying NH perturbation strengths: (a) $h_z/t_0 = 0.8$, (b) $h_z/t_0 = 0.9$, (c) $h_z/t_0 = 1.0$, (d) $h_z/t_0 = 1.1$, and (e) $h_z/t_0 = 1.2$. In each subfigure, the panels from top to bottom show the experimental results, the theoretical prediction for the clean (disorder-free) case, and the theoretical calculation including disorder, respectively.

S6 Extended experimental results

In this section of the Supplemental Material, we display additional results from our experimental measurements and their comparison with theoretical predictions. The outcomes are summarized in Table 1 and the results are explicitly displayed in Figs. S20-S71. While discussion the results we will be using a few abbreviations. So, we announce them up front. Specifically, NH stands for non-Hermitian, PBCs stand for perodic boundary conditions, OBCs stand for open boundary conditions, NHDS stands for non-Hermitian dislocation states, and D-NHSE stands for dislocation induced non-Hermitian skin effect.

Table 1: Figure references of the extended experimental results. Parameters are $\omega_0 = 1040\,\text{Hz} - 4.5\text{i}\,\text{Hz}$ and $t_0 = 3\,\text{Hz}$.

Figure label	Boundary condition	m_0/t_0	h_x/t_0	h_y/t_0	h_z/t_0	Comment
S20	PBC	-1	0	0	0	Zero-energy dislocation bound states.
S21	OBC	-1	0	0	0	Zero-energy dislocation bound states. Boundary states.
S22	PBC	1	0	0	0	No dislocation bound states.
S23	OBC	1	0	0	0	No dislocation bound states. Mid-gap boundary states.
S24	PBC	-1	0.3	0	0	Zero-energy NHDSs. Asymmetric NHDS distribution along x direction. No D-NHSE.
S25	OBC	-1	0.3	0	0	Zero-energy NHDSs. Asymmetric NHDS distribution along x direction. NHSE along x direction.
S26	PBC	1	0.3	0	0	No NHDS. No D-NHSE.
S27	OBC	1	0.3	0	0	No NHDS. NHSE along x direction.
S28	PBC	-1	0.6	0	0	Zero-energy NHDS. No D-NHSE.
S29	OBC	-1	0.6	0	0	Zero-energy NHDS. Asymmetric NHDS distribution along x direction. NHSE along x direction.
S30	PBC	1	0.6	0	0	No NHDS. No D-NHSE.
S31	OBC	1	0.6	0	0	No NHDS. NHSE along x direction.
S32	PBC	-1	0.9	0	0	Zero-energy NHDS with weights at the dislocation cores start to show signs of diminishing. No D-NHSE.

Continued on next page

Figure label	Boundary condition	m_0/t_0	h_x/t_0	h_y/t_0	h_z/t_0	Comment
S33	OBC	-1	0.9	0	0	Zero-energy NHDSs with weights at the dislocation cores start to show signs of diminishing. Asymmetric NHDS distribution along x direction. NHSE along x direction.
S34	PBC	1	0.9	0	0	No NHDS. No D-NHSE.
S35	OBC	1	0.9	0	0	No NHDS. NHSE along x direction.
S36	PBC	-1	1.1	0	0	No NHDS. No D-NHSE.
S37	OBC	-1	1.1	0	0	No NHDS. NHSE along x direction.
S38	PBC	1	1.1	0	0	No NHDS. No D-NHSE.
S39	OBC	1	1.1	0	0	No NHDS. NHSE along x direction.
S40	PBC	-1	0	0.3	0	NHDS. D-NHSE along y direction.
S41	OBC	-1	0	0.3	0	Slightly delocalized NHDS. NHSE along y directin.
S42	PBC	1	0	0.3	0	No NHDS. D-NHSE along y direction.
S43	OBC	1	0	0.3	0	No NHDS. NHSE along y direction.
S44	PBC	-1	0	0.6	0	NHDS. D-NHSE along y direction.
S45	OBC	-1	0	0.6	0	Slightly delocalized NHDS. NHSE along y direction.
S46	PBC	1	0	0.6	0	No NHDS. D-NHSE along y direction.
S47	OBC	1	0	0.6	0	No NHDS. NHSE along y direction.
S48	PBC	-1	0	0.9	0	NHDS. D-NHSE along y direction.
S49	OBC	-1	0	0.9	0	Substantially delocalized NHDS. NHSE along y direction.
S50	PBC	1	0	0.9	0	No NHDS. D-NHSE along y direction.
S51	OBC	1	0	0.9	0	No NHDS. NHSE along y direction.
S52	PBC	-1	0	1.1	0	No NHDS. D-NHSE along y direction.
S53	OBC	-1	0	1.1	0	No NHDS. NHSE along y direction.
S54	PBC	1	0	1.1	0	No NHDS. D-NHSE along y direction.
S55	OBC	1	0	1.1	0	No NHDS. NHSE along y direction.
S56	PBC	-1	0	0	0.3	NHDS. No D-NHSE.
S57	OBC	-1	0	0	0.3	NHDS. Boundary states. No NHSE.
S58	PBC	1	0	0	0.3	No NHDS. No D-NHSE.
S59	OBC	1	0	0	0.3	No NHDS. Boundary states. No NHSE.
S60	PBC	-1	0	0	0.6	NHDS. No D-NHSE.

Continued on next page

Figure label	Boundary condition	m_0/t_0	h_x/t_0	h_y/t_0	h_z/t_0	Comment
S61	OBC	-1	0	0	0.6	Slightly delocalized NHDS and boundary states. No NHSE.
S62	PBC	1	0	0	0.6	No NHDS. No D-NHSE.
S63	OBC	1	0	0	0.6	No NHDS. No NHSE.
S64	PBC	-1	0	0	0.9	NHDS. No D-NHSE.
S65	OBC	-1	0	0	0.9	Substantially delocalized NHDS. No NHSE.
S66	PBC	1	0	0	0.9	No NHDS. No D-NHSE.
S67	OBC	1	0	0	0.9	No NHDS. No NHSE.
S68	PBC	-1	0	0	1.1	No NHDS. No D-NHSE.
S69	OBC	-1	0	0	1.1	No NHDS. No NHSE.
S70	PBC	1	0	0	1.1	No NHDS. No D-NHSE.
S71	OBC	1	0	0	1.1	No NHDS. No NHSE.

End of table

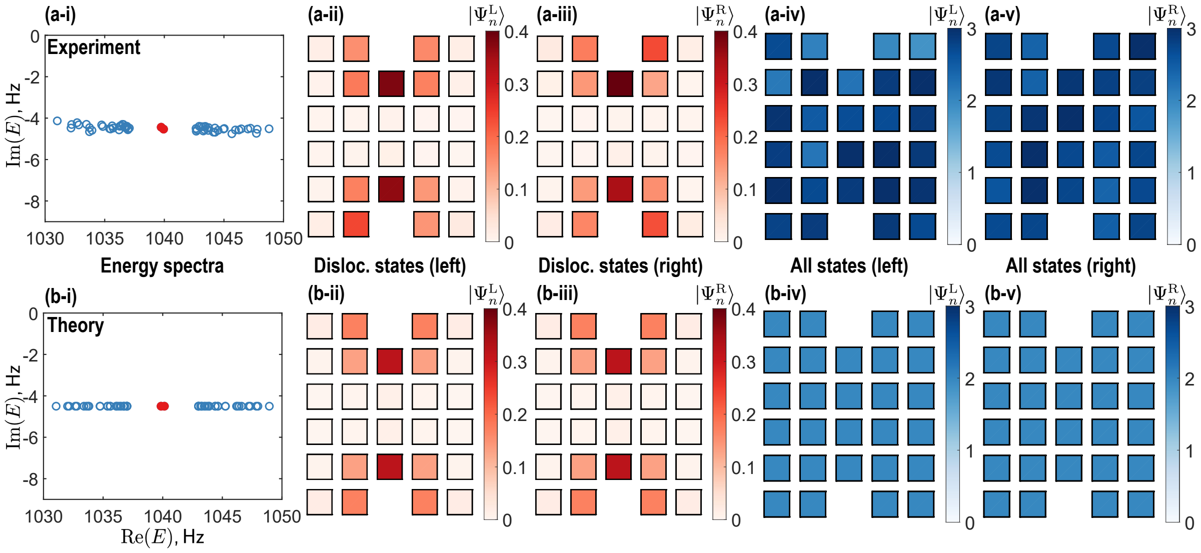

Figure S20: (a) Experimental results and (b) theoretical predictions of Hermitian acoustic Chern insulators with an edge dislocation-antidislocation pair under PBCs in the M phase. (i) Energy spectra. Red dots ●, dislocation states; Blue circles ○, other states. Amplitude distributions of the (ii) left and (iii) right eigenstates for the dislocation states. Summed amplitude distributions of all (iv) left and (v) right eigenstates. Parameters: $\omega_0 = 1040\,\text{Hz} - 4.5\text{i}\,\text{Hz}, t_0 = -m_0 = 3\,\text{Hz}, h_x = h_y = h_z = 0$.

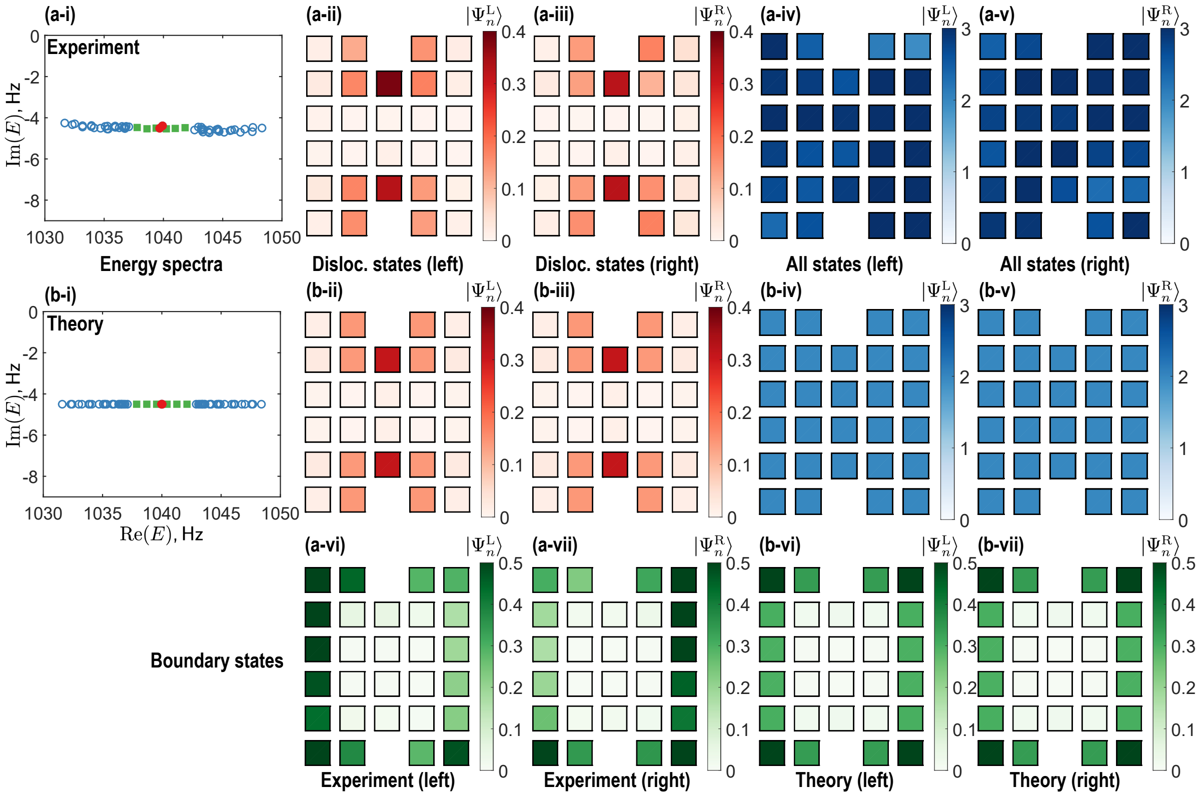

Figure S21: (a) Experimental results and (b) theoretical predictions of Hermitian acoustic Chern insulators with an edge dislocation-antidislocation pair under OBCs in the M phase. (i) Energy spectra. Red dots ●, dislocation states; green squares ■, boundary states; blue circles ○, all other states. Amplitude distributions of the (ii) left and (iii) right eigenstates for the dislocation states. Summed amplitude distributions of all (iv) left and (v) right eigenstates. Amplitude distributions of the (vi) left and (vii) right eigenstates for the boundary states. Parameters: $\omega_0 = 1040\,\text{Hz} - 4.5\text{i}\,\text{Hz}$, $t_0 = -m_0 = 3\,\text{Hz}$, and $h_x = h_y = h_z = 0$.

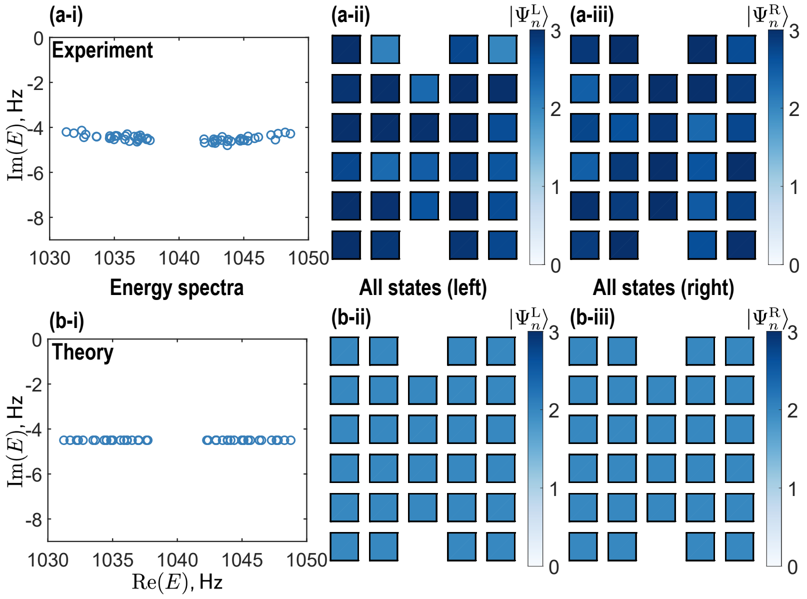

Figure S22: (a) Experimental results and (b) theoretical predictions of Hermitian acoustic Chern insulators with an edge dislocation-antidislocation pair under PBCs in the Γ phase. (i) Energy spectra. Summed amplitude distributions of all (ii) left and (iii) right eigenstates. Parameters: $\omega_0 = 1040\,\text{Hz} - 4.5\text{i}\,\text{Hz}, t_0 = m_0 = 3\,\text{Hz}, h_x = h_y = h_z = 0$.

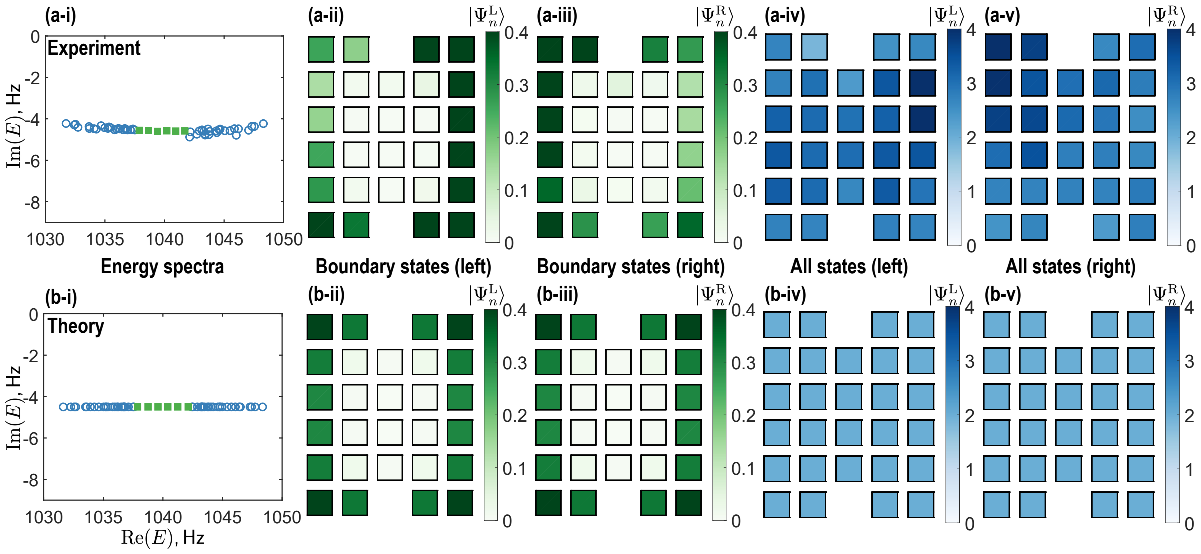

Figure S23: (a) Experimental results and (b) theoretical predictions of Hermitian acoustic Chern insulators with an edge dislocation-antidislocation pair under OBCs in the Γ phase. (i) Energy spectra. Green squares ■, boundary states; Blue circles ○, other states. Amplitude distributions of the (ii) left and (iii) right eigenstates for the boundary states. Summed amplitude distributions of all (iv) left and (v) right eigenstates. Parameters: $\omega_0 = 1040\,\text{Hz} - 4.5\text{i}\,\text{Hz}, t_0 = m_0 = 3\,\text{Hz}, h_x = h_y = h_z = 0$.

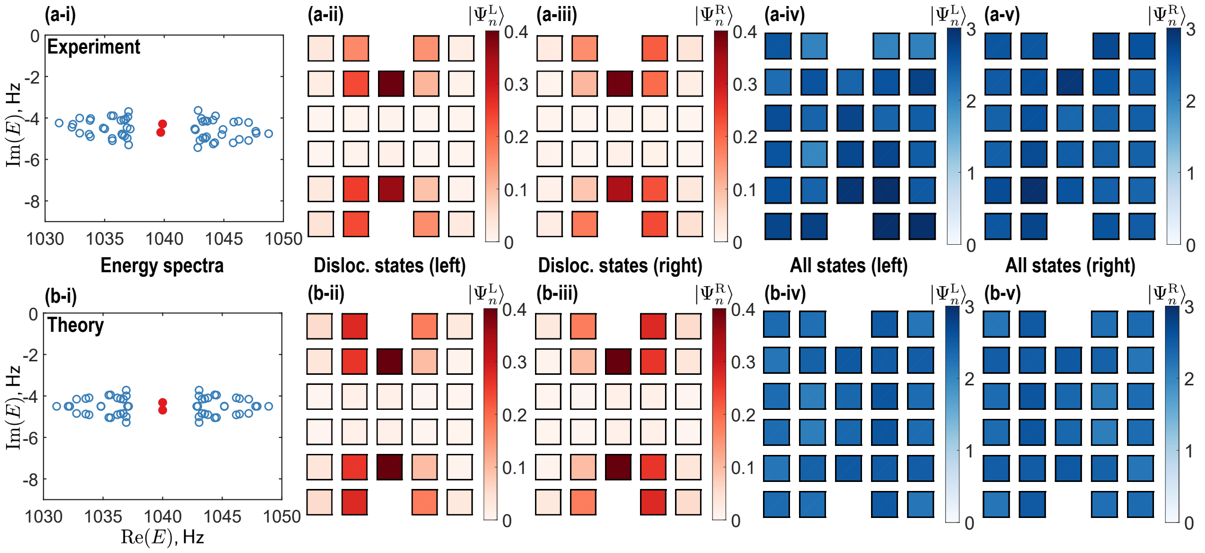

Figure S24: (a) Experimental results and (b) theoretical predictions of NH acoustic Chern insulators with an edge dislocation-antidislocation pair under PBCs in the M phase. (i) Energy spectra. Red dots ●, dislocation states; Blue circles ○, other states. Amplitude distributions of the (ii) left and (iii) right eigenstates for the NHDSs. Summed amplitude distributions of all (iv) left and (v) right eigenstates. Parameters: $\omega_0 = 1040\,\text{Hz} - 4.5\text{i}\,\text{Hz}, t_0 = -m_0 = 3\,\text{Hz}, h_x = 0.9\,\text{Hz}, h_y = h_z = 0$.

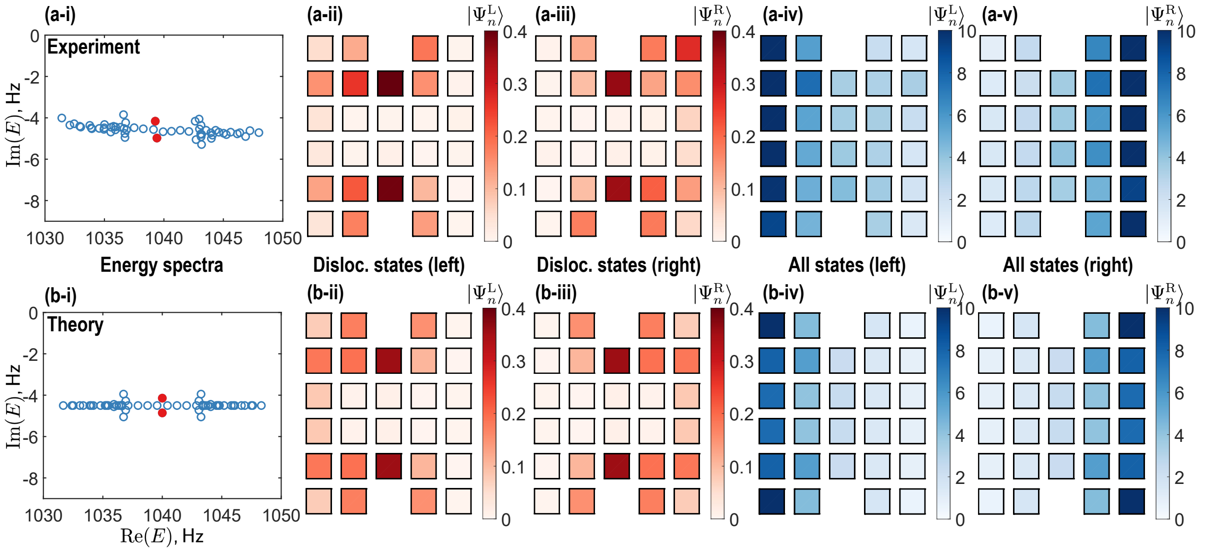

Figure S25: (a) Experimental results and (b) theoretical predictions of NH acoustic Chern insulators with an edge dislocation-antidislocation pair under OBCs in the M phase. (i) Energy spectra. Red dots ●, dislocation states; blue circles ○, all other states. Amplitude distributions of the (ii) left and (iii) right eigenstates for the dislocation states. Summed amplitude distributions of all (iv) left and (v) right eigenstates. Parameters: $\omega_0 = 1040\,\text{Hz} - 4.5\text{i}\,\text{Hz}, t_0 = -m_0 = 3\,\text{Hz}, h_x = 0.9\,\text{Hz}, h_y = h_z = 0$.

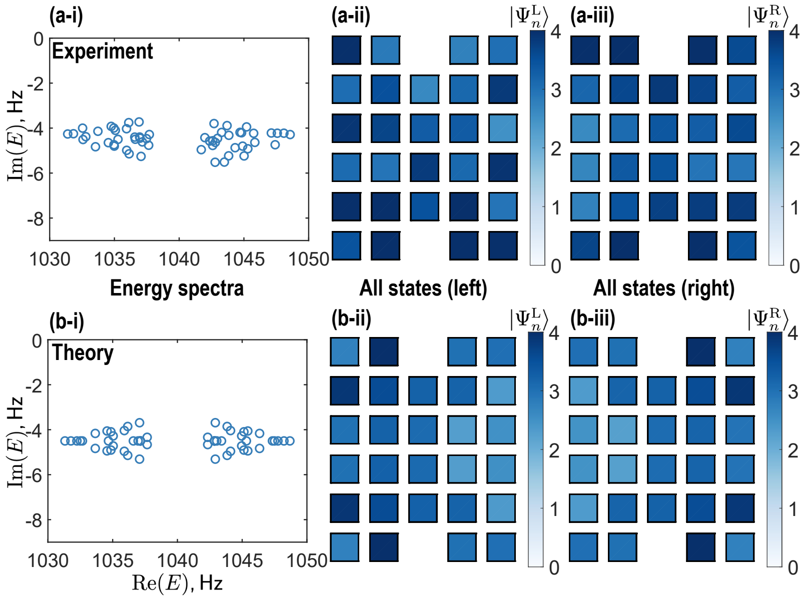

Figure S26: (a) Experimental results and (b) theoretical predictions of NH acoustic Chern insulators with an edge dislocation-antidislocation pair under PBCs in the Γ phase. (i) Energy spectra. Summed amplitude distributions of all (ii) left and (iii) right eigenstates. Parameters: $\omega_0 = 1040\,\mathrm{Hz} - 4.5\mathrm{i}\,\mathrm{Hz}, t_0 = m_0 = 3\,\mathrm{Hz}, h_x = 0.9\,\mathrm{Hz}, h_y = h_z = 0$.

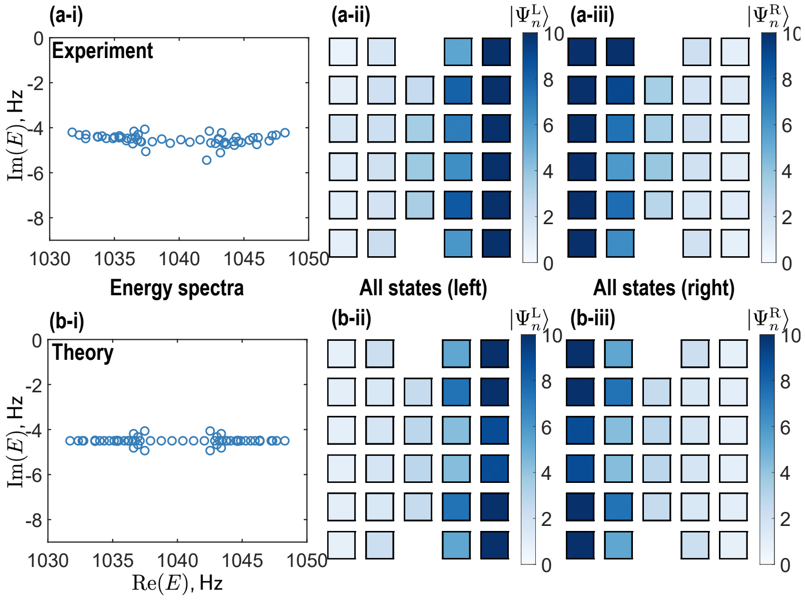

Figure S27: (a) Experimental results and (b) theoretical predictions of NH acoustic Chern insulators with an edge dislocation-antidislocation pair under OBCs in the Γ phase. (i) Energy spectra. Summed amplitude distributions of all (ii) left and (iii) right eigenstates. Parameters: $\omega_0 = 1040\,\mathrm{Hz} - 4.5\mathrm{i}\,\mathrm{Hz}, t_0 = m_0 = 3\,\mathrm{Hz}, h_x = 0.9\,\mathrm{Hz}, h_y = h_z = 0$.

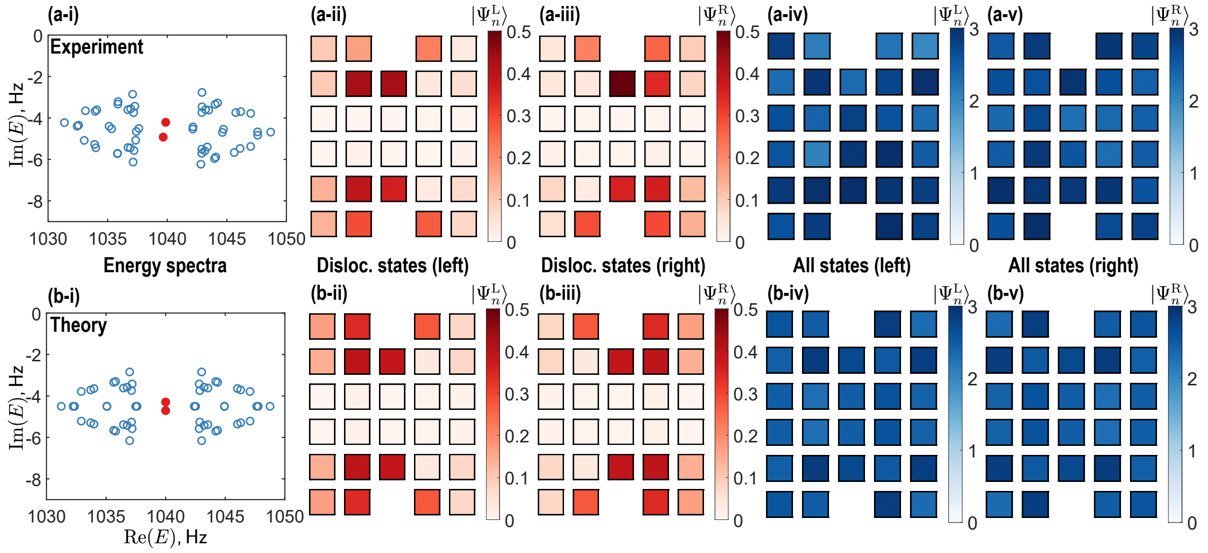

Figure S28: (a) Experimental results and (b) theoretical predictions of NH acoustic Chern insulators with an edge dislocation-antidislocation pair under PBCs in the M phase. (i) Energy spectra. Red dots ●, dislocation states; Blue circles ○, other states. Amplitude distributions of the (ii) left and (iii) right eigenstates for the NHDSs. Summed amplitude distributions of all (iv) left and (v) right eigenstates. Parameters: $\omega_0 = 1040\,\mathrm{Hz} - 4.5\mathrm{i}\,\mathrm{Hz}, t_0 = -m_0 = 3\,\mathrm{Hz}, h_x = 1.8\,\mathrm{Hz}, h_y = h_z = 0$.

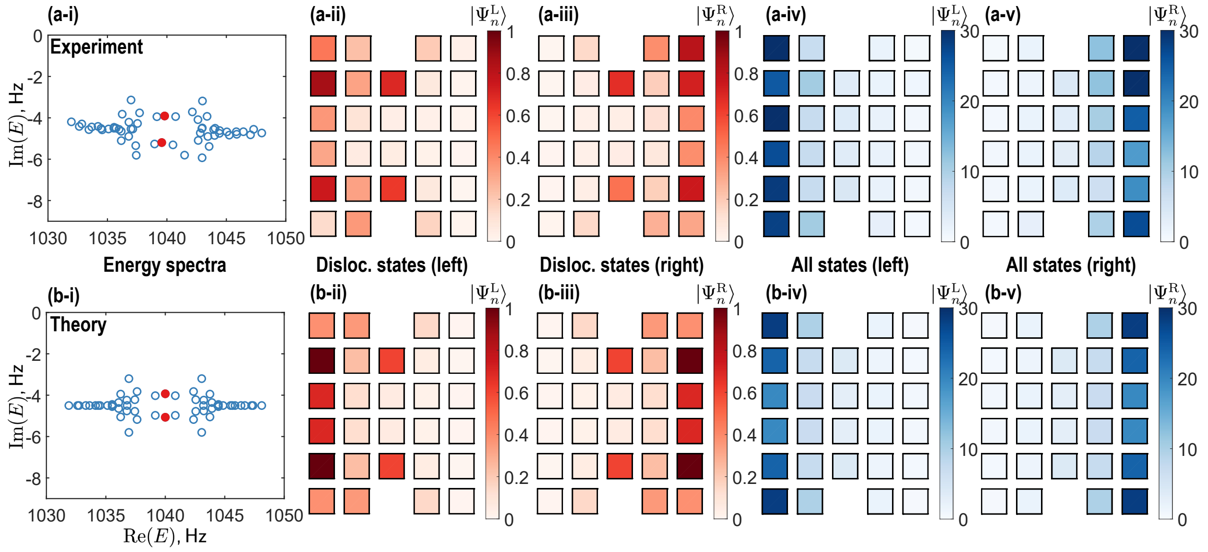

Figure S29: (a) Experimental results and (b) theoretical predictions of NH acoustic Chern insulators with an edge dislocation-antidislocation pair under OBCs in the M phase. (i) Energy spectra. Red dots ●, dislocation states; Blue circles ○, other states. Amplitude distributions of the (ii) left and (iii) right eigenstates for the NHDSs. Summed amplitude distributions of all (iv) left and (v) right eigenstates. Parameters: $\omega_0 = 1040\,\mathrm{Hz} - 4.5\mathrm{i}\,\mathrm{Hz}, t_0 = -m_0 = 3\,\mathrm{Hz}, h_x = 1.8\,\mathrm{Hz}, h_y = h_z = 0$.

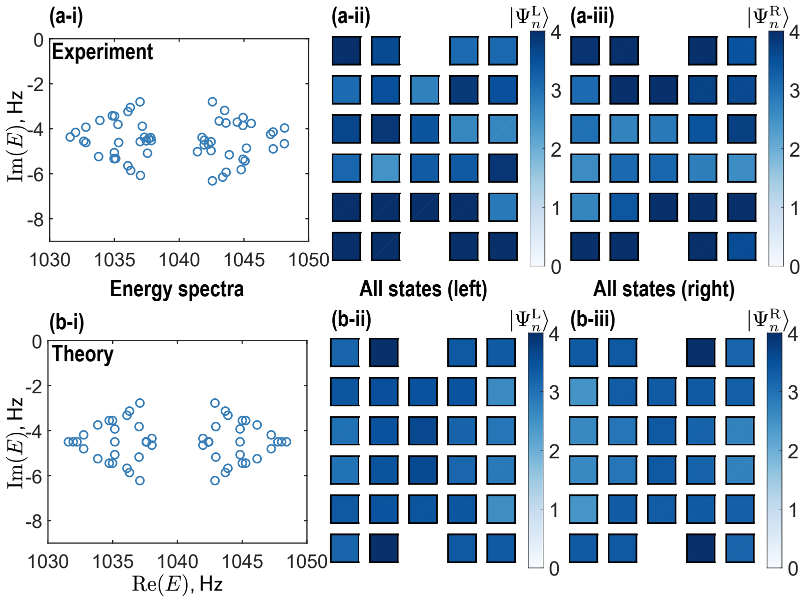

Figure S30: (a) Experimental results and (b) theoretical predictions of NH acoustic Chern insulators with an edge dislocation-antidislocation pair under PBCs in the Γ phase. (i) Energy spectra. Summed amplitude distributions of all (ii) left and (iii) right eigenstates. Parameters: $\omega_0 = 1040\,\text{Hz} - 4.5\text{i}\,\text{Hz}, t_0 = m_0 = 3\,\text{Hz}, h_x = 1.8\,\text{Hz}, h_y = h_z = 0$.

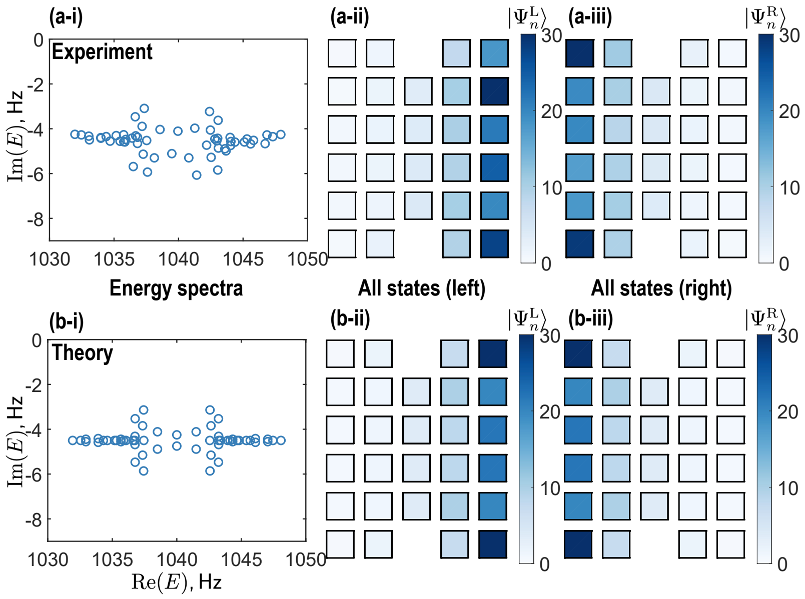

Figure S31: (a) Experimental results and (b) theoretical predictions of NH acoustic Chern insulators with an edge dislocation-antidislocation pair under OBCs in the Γ phase. (i) Energy spectra. Summed amplitude distributions of all (ii) left and (iii) right eigenstates. Parameters: $\omega_0 = 1040\,\text{Hz} - 4.5\text{i}\,\text{Hz}, t_0 = m_0 = 3\,\text{Hz}, h_x = 1.8\,\text{Hz}, h_y = h_z = 0$.

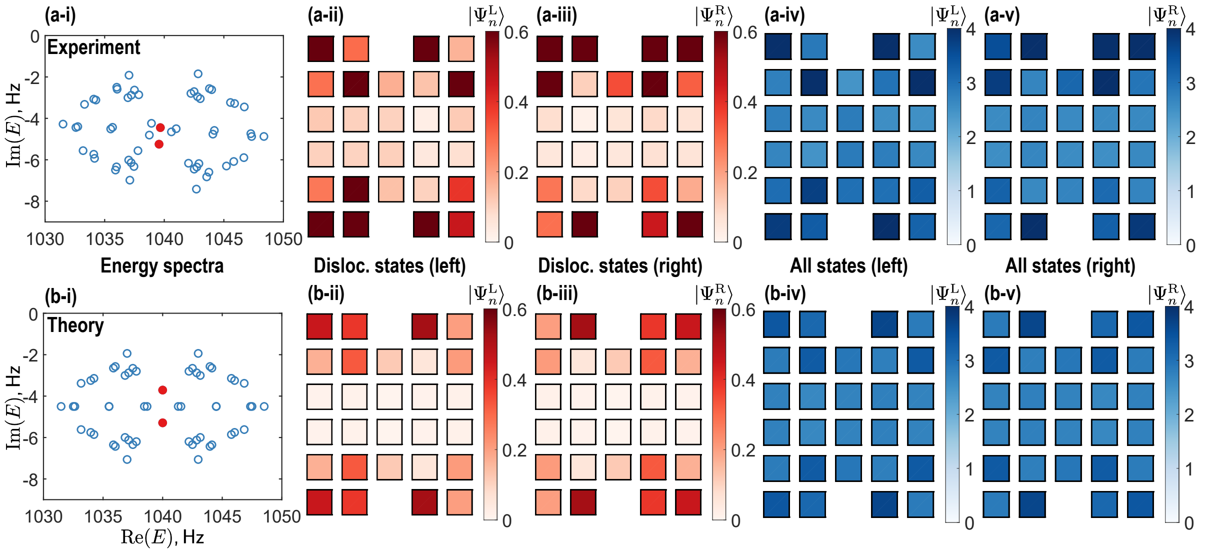

Figure S32: (a) Experimental results and (b) theoretical predictions of NH acoustic Chern insulators with an edge dislocation-antidislocation pair under PBCs in the M phase. (i) Energy spectra. Red dots ●, dislocation states; Blue circles ○, other states. Amplitude distributions of the (ii) left and (iii) right eigenstates for the NHDSs. Summed amplitude distributions of all (iv) left and (v) right eigenstates. Parameters: $\omega_0 = 1040\,\mathrm{Hz} - 4.5\mathrm{i}\,\mathrm{Hz}, t_0 = -m_0 = 3\,\mathrm{Hz}, h_x = 2.7\,\mathrm{Hz}, h_y = h_z = 0$.

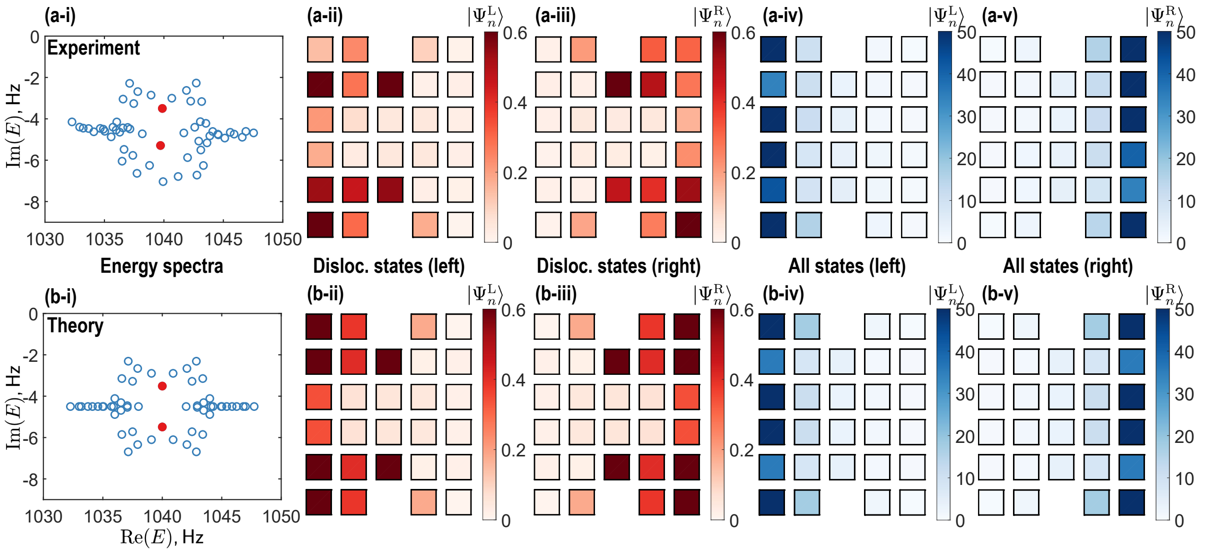

Figure S33: (a) Experimental results and (b) theoretical predictions of NH acoustic Chern insulators with an edge dislocation-antidislocation pair under OBCs in the M phase. (i) Energy spectra. Red dots ●, dislocation states; Blue circles ○, other states. Amplitude distributions of the (ii) left and (iii) right eigenstates for the NHDSs. Summed amplitude distributions of all (iv) left and (v) right eigenstates. Parameters: $\omega_0 = 1040\,\mathrm{Hz} - 4.5\mathrm{i}\,\mathrm{Hz}, t_0 = -m_0 = 3\,\mathrm{Hz}, h_x = 2.7\,\mathrm{Hz}, h_y = h_z = 0$.

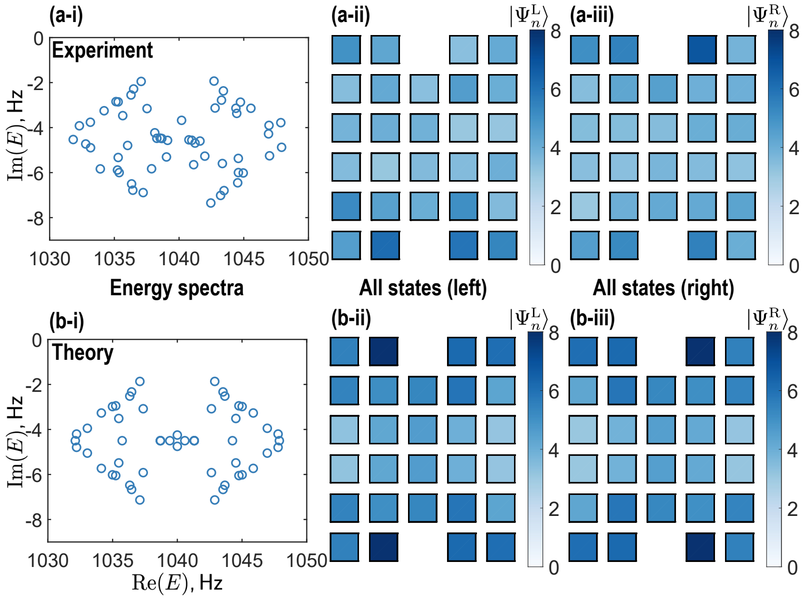

Figure S34: (a) Experimental results and (b) theoretical predictions of NH acoustic Chern insulators with an edge dislocation-antidislocation pair under PBCs in the Γ phase. (i) Energy spectra. Summed amplitude distributions of all (ii) left and (iii) right eigenstates. Parameters: $\omega_0 = 1040\,\mathrm{Hz} - 4.5\mathrm{i}\,\mathrm{Hz}, t_0 = m_0 = 3\,\mathrm{Hz}, h_x = 2.7\,\mathrm{Hz}, h_y = h_z = 0$.

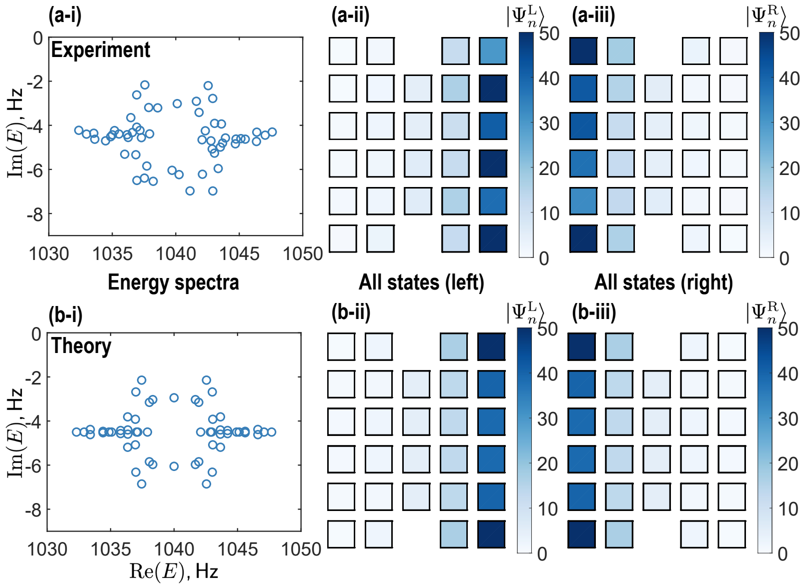

Figure S35: (a) Experimental results and (b) theoretical predictions of NH acoustic Chern insulators with an edge dislocation-antidislocation pair under OBCs in the Γ phase. (i) Energy spectra. Summed amplitude distributions of all (ii) left and (iii) right eigenstates. Parameters: $\omega_0 = 1040\,\mathrm{Hz} - 4.5\mathrm{i}\,\mathrm{Hz}, t_0 = m_0 = 3\,\mathrm{Hz}, h_x = 2.7\,\mathrm{Hz}, h_y = h_z = 0$.

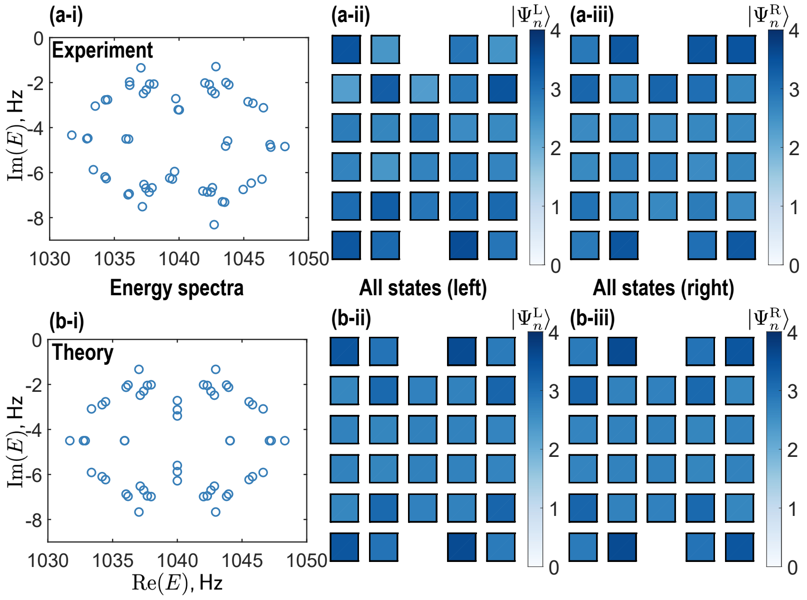

Figure S36: (a) Experimental results and (b) theoretical predictions of NH acoustic Chern insulators with an edge dislocation-antidislocation pair under PBCs in the M phase. (i) Energy spectra. Summed amplitude distributions of all (ii) left and (iii) right eigenstates. Parameters: $\omega_0 = 1040\,\mathrm{Hz} - 4.5\mathrm{i}\,\mathrm{Hz}, t_0 = -m_0 = 3\,\mathrm{Hz}, h_x = 3.3\,\mathrm{Hz}, h_y = h_z = 0$.

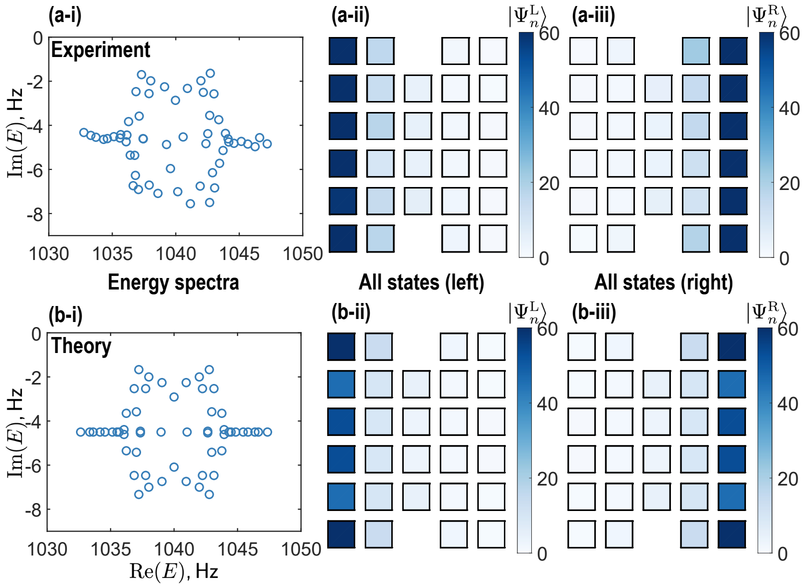

Figure S37: (a) Experimental results and (b) theoretical predictions of NH acoustic Chern insulators with an edge dislocation-antidislocation pair under PBCs in the M phase. (i) Energy spectra. Summed amplitude distributions of all (ii) left and (iii) right eigenstates. Parameters: $\omega_0 = 1040\,\mathrm{Hz} - 4.5\mathrm{i}\,\mathrm{Hz}, t_0 = -m_0 = 3\,\mathrm{Hz}, h_x = 3.3\,\mathrm{Hz}, h_y = h_z = 0$.

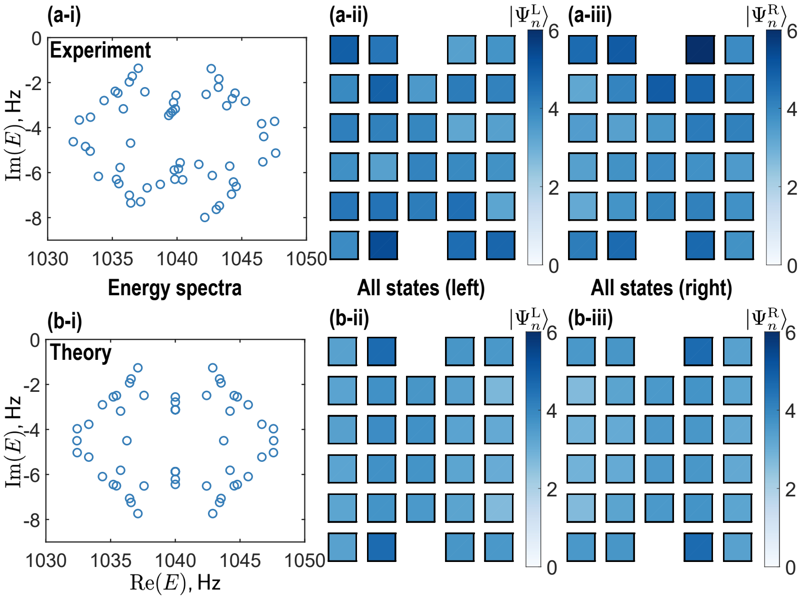

Figure S38: (a) Experimental results and (b) theoretical predictions of NH acoustic Chern insulators with an edge dislocation-antidislocation pair under PBCs in the Γ phase. (i) Energy spectra. Summed amplitude distributions of all (ii) left and (iii) right eigenstates. Parameters: $\omega_0 = 1040\,\text{Hz} - 4.5\text{i}\,\text{Hz}, t_0 = m_0 = 3\,\text{Hz}, h_x = 3.3\,\text{Hz}, h_y = h_z = 0$.

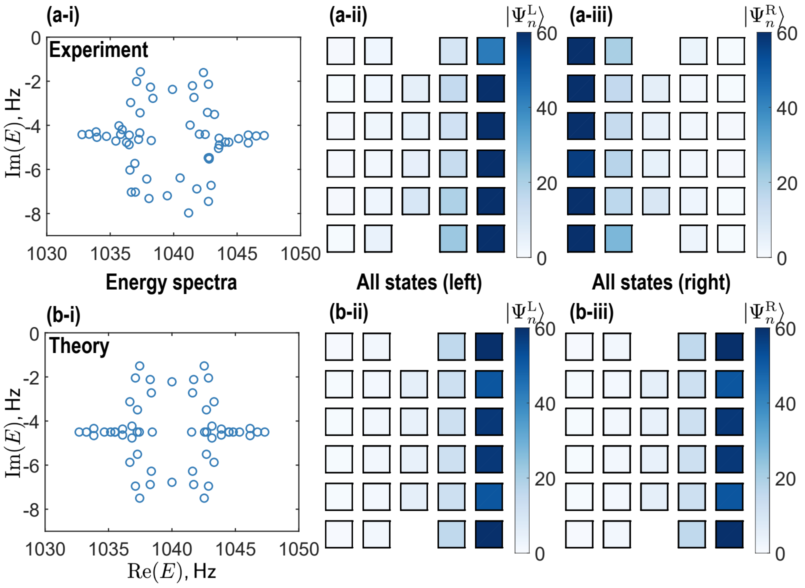

Figure S39: (a) Experimental results and (b) theoretical predictions of NH acoustic Chern insulators with an edge dislocation-antidislocation pair under PBCs in the Γ phase. (i) Energy spectra. Summed amplitude distributions of all (ii) left and (iii) right eigenstates. Parameters: $\omega_0 = 1040\,\text{Hz} - 4.5\text{i}\,\text{Hz}, t_0 = m_0 = 3\,\text{Hz}, h_x = 3.3\,\text{Hz}, h_y = h_z = 0$.

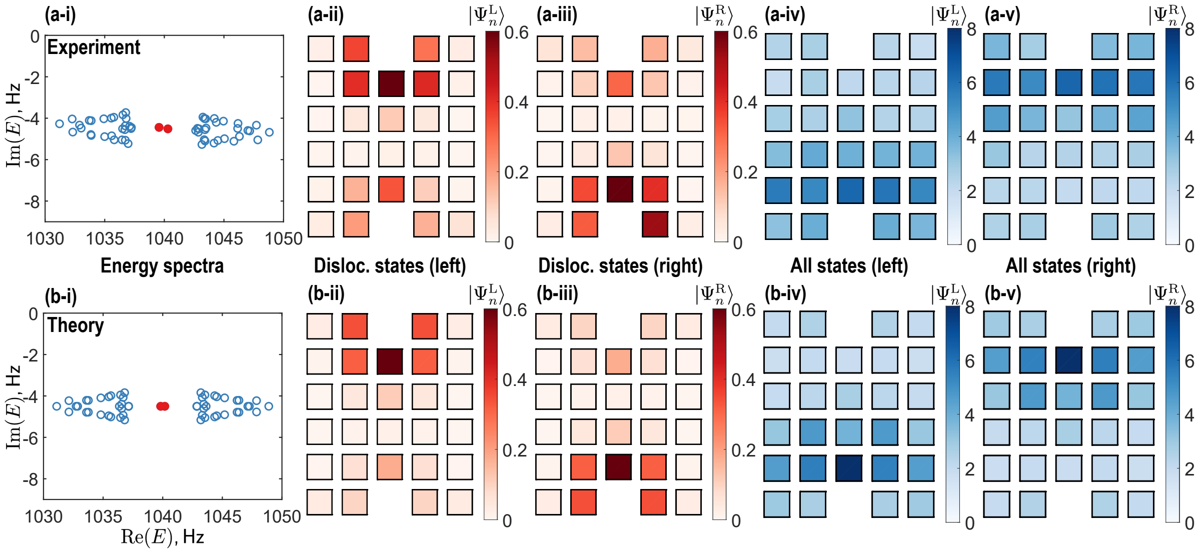

Figure S40: (a) Experimental results and (b) theoretical predictions of NH acoustic Chern insulators with an edge dislocation-antidislocation pair under PBCs in the M phase. (i) Energy spectra. Red dots ●, dislocation states; Blue circles ○, other states. Amplitude distributions of the (ii) left and (iii) right eigenstates for the NHDSs. Summed amplitude distributions of all (iv) left and (v) right eigenstates. Parameters: $\omega_0 = 1040\,\text{Hz} - 4.5\text{i}\,\text{Hz}, t_0 = -m_0 = 3\,\text{Hz}, h_y = 0.9\,\text{Hz}, h_x = h_z = 0$.

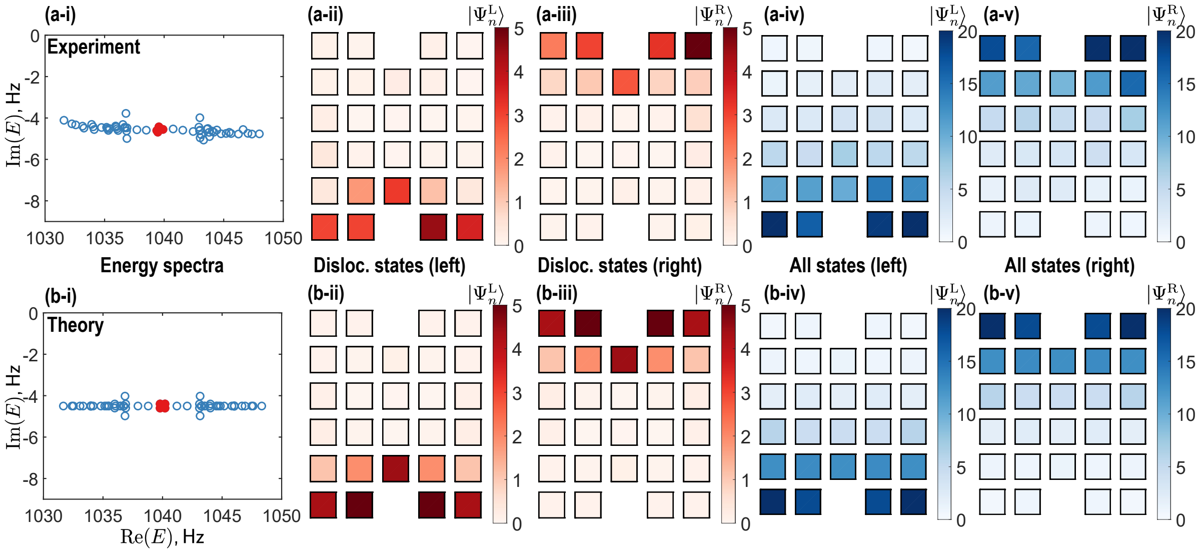

Figure S41: (a) Experimental results and (b) theoretical predictions of NH acoustic Chern insulators with an edge dislocation-antidislocation pair under OBCs in the M phase. (i) Energy spectra. Red dots ●, dislocation states; Blue circles ○, other states. Summed amplitude distributions of the (ii) left and (iii) right eigenstates for the NHDS. Summed amplitude distributions of all (iv) left and (v) right eigenstates. Parameters: $\omega_0 = 1040\,\text{Hz} - 4.5\text{i}\,\text{Hz}, t_0 = -m_0 = 3\,\text{Hz}, h_y = 0.9\,\text{Hz}, h_x = h_z = 0$.

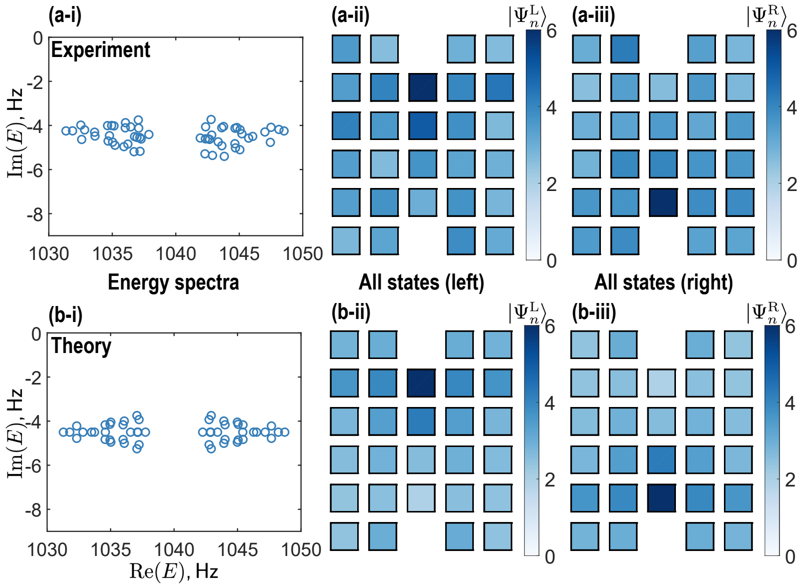

Figure S42: (a) Experimental results and (b) theoretical predictions of NH acoustic Chern insulators with an edge dislocation-antidislocation pair under PBCs in the Γ phase. (i) Energy spectra. Summed amplitude distributions of all (ii) left and (iii) right eigenstates. Parameters: $\omega_0 = 1040\,\text{Hz} - 4.5\text{i}\,\text{Hz}, t_0 = m_0 = 3\,\text{Hz}, h_y = 0.9\,\text{Hz}, h_x = h_z = 0$.

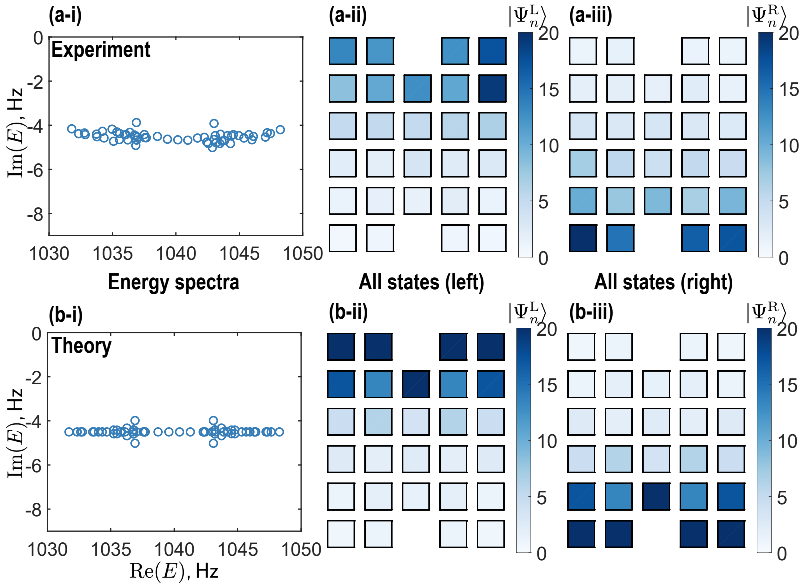

Figure S43: (a) Experimental results and (b) theoretical predictions of NH acoustic Chern insulators with an edge dislocation-antidislocation pair under OBCs in the Γ phase. (i) Energy spectra. Summed amplitude distributions of all (ii) left and (iii) right eigenstates. Parameters: $\omega_0 = 1040\,\text{Hz} - 4.5\text{i}\,\text{Hz}, t_0 = m_0 = 3\,\text{Hz}, h_y = 0.9\,\text{Hz}, h_x = h_z = 0$.

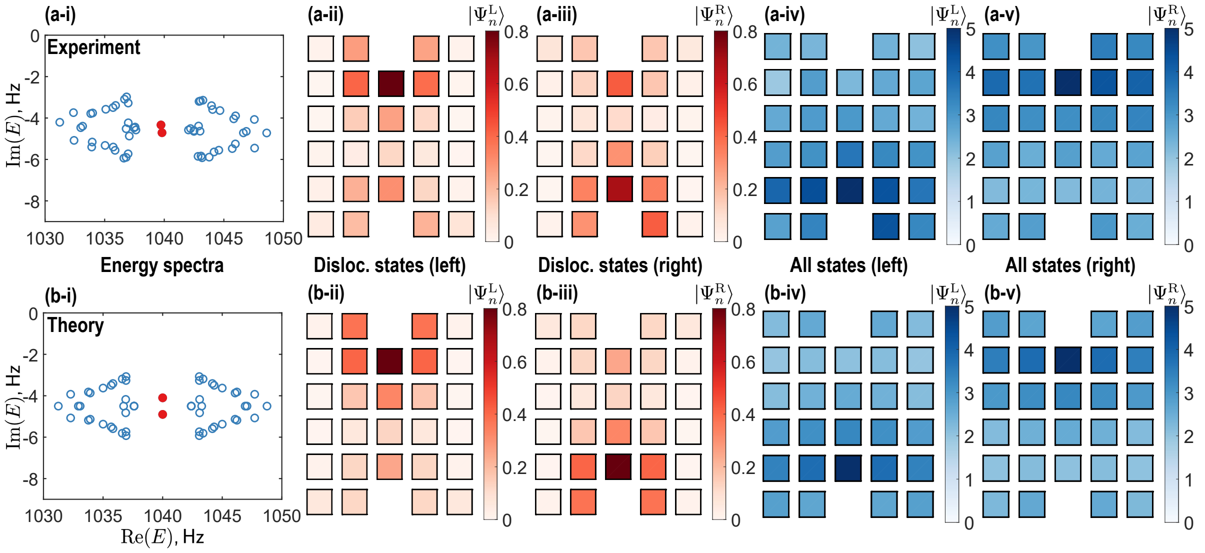

Figure S44: (a) Experimental results and (b) theoretical predictions of NH acoustic Chern insulators with an edge dislocation-antidislocation pair under PBCs in the M phase. (i) Energy spectra. Red dots ●, dislocation states; Blue circles ○, other states. Amplitude distributions of the (ii) left and (iii) right eigenstates for the NHDSs. Summed amplitude distributions of all (iv) left and (v) right eigenstates. Parameters: $\omega_0 = 1040\,\mathrm{Hz} - 4.5\mathrm{i}\,\mathrm{Hz}, t_0 = -m_0 = 3\,\mathrm{Hz}, h_y = 1.8\,\mathrm{Hz}, h_x = h_z = 0$.

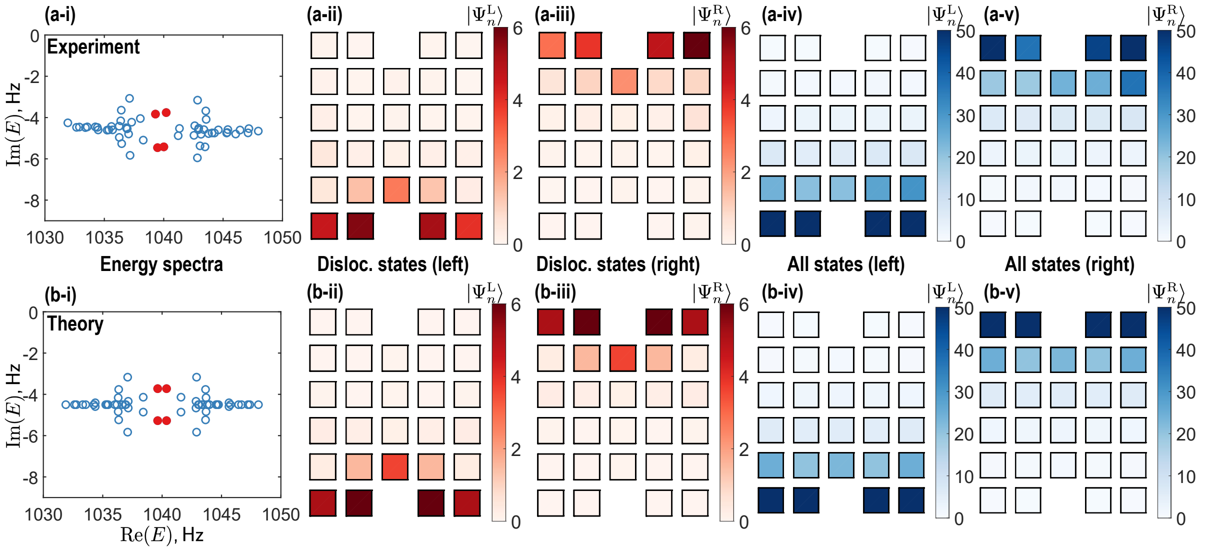

Figure S45: (a) Experimental results and (b) theoretical predictions of NH acoustic Chern insulators with an edge dislocation-antidislocation pair under OBCs in the M phase. (i) Energy spectra. Red dots ●, dislocation states; Blue circles ○, other states. Summed amplitude distributions of the (ii) left and (iii) right eigenstates for the NHDS. Summed amplitude distributions of all (iv) left and (v) right eigenstates. Parameters: $\omega_0 = 1040\,\mathrm{Hz} - 4.5\mathrm{i}\,\mathrm{Hz}, t_0 = -m_0 = 3\,\mathrm{Hz}, h_y = 1.8\,\mathrm{Hz}, h_x = h_z = 0$.

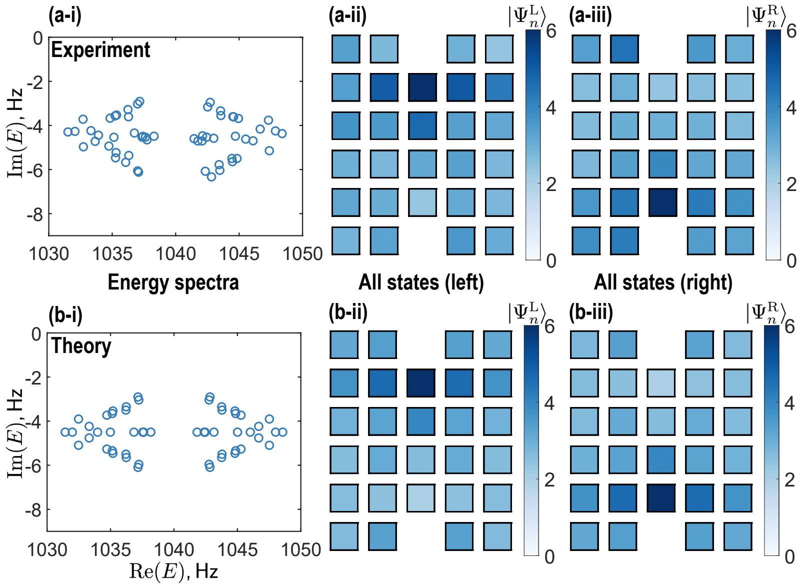

Figure S46: (a) Experimental results and (b) theoretical predictions of NH acoustic Chern insulators with an edge dislocation-antidislocation pair under PBCs in the Γ phase. (i) Energy spectra. Summed amplitude distributions of all (ii) left and (iii) right eigenstates. Parameters: $\omega_0 = 1040\,\text{Hz} - 4.5\text{i}\,\text{Hz}, t_0 = m_0 = 3\,\text{Hz}, h_y = 1.8\,\text{Hz}, h_x = h_z = 0$.

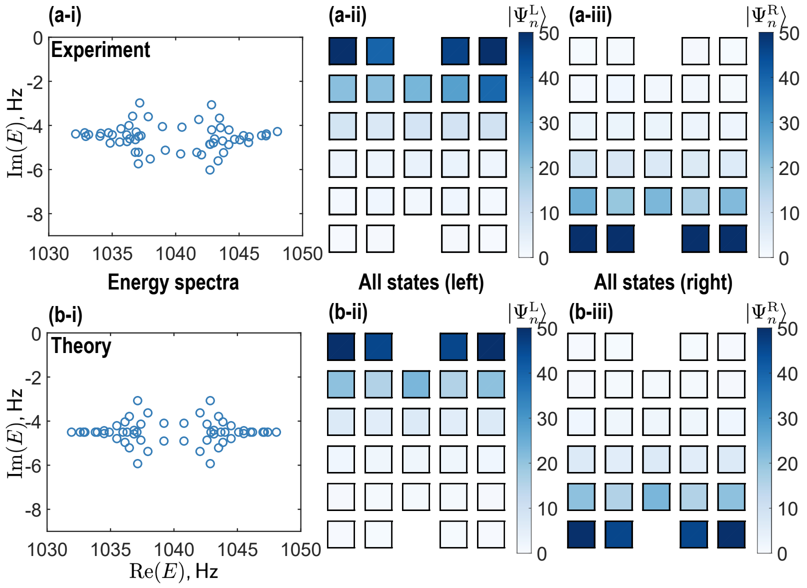

Figure S47: (a) Experimental results and (b) theoretical predictions of NH acoustic Chern insulators with an edge dislocation-antidislocation pair under OBCs in the Γ phase. (i) Energy spectra. Summed amplitude distributions of all (ii) left and (iii) right eigenstates. Parameters: $\omega_0 = 1040\,\text{Hz} - 4.5\text{i}\,\text{Hz}, t_0 = m_0 = 3\,\text{Hz}, h_y = 1.8\,\text{Hz}, h_x = h_z = 0$.

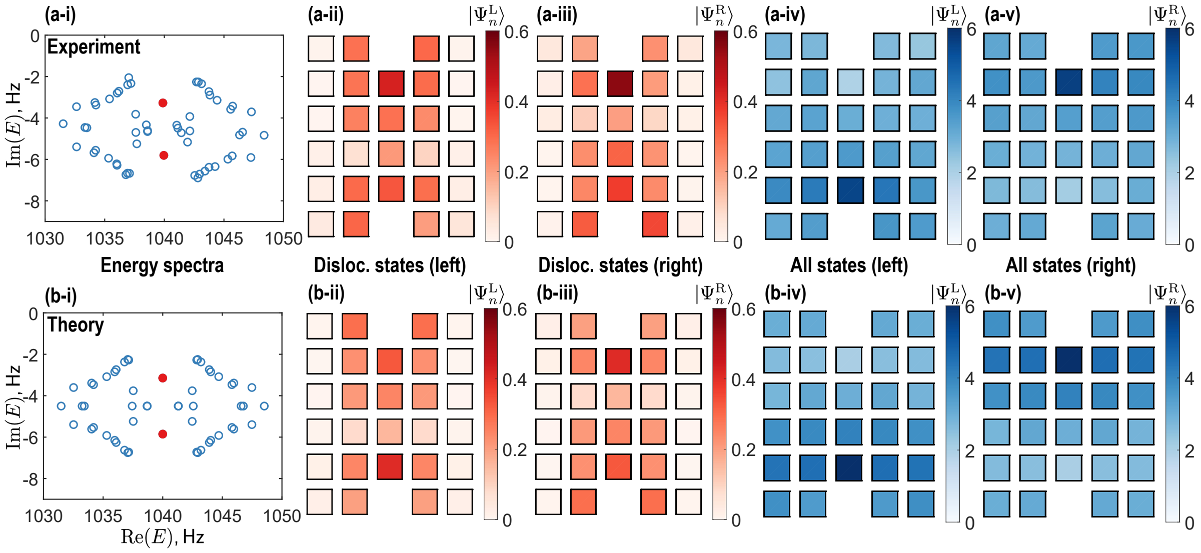

Figure S48: (a) Experimental results and (b) theoretical predictions of NH acoustic Chern insulators with an edge dislocation-antidislocation pair under PBCs in the M phase. (i) Energy spectra. Red dots ●, dislocation states; Blue circles ○, other states. Amplitude distributions of the (ii) left and (iii) right eigenstates for the NHDS. Summed amplitude distributions of all (iv) left and (v) right eigenstates. Parameters: $\omega_0 = 1040\,\text{Hz} - 4.5\text{i}\,\text{Hz}, t_0 = -m_0 = 3\,\text{Hz}, h_y = 2.7\,\text{Hz}, h_x = h_z = 0$.

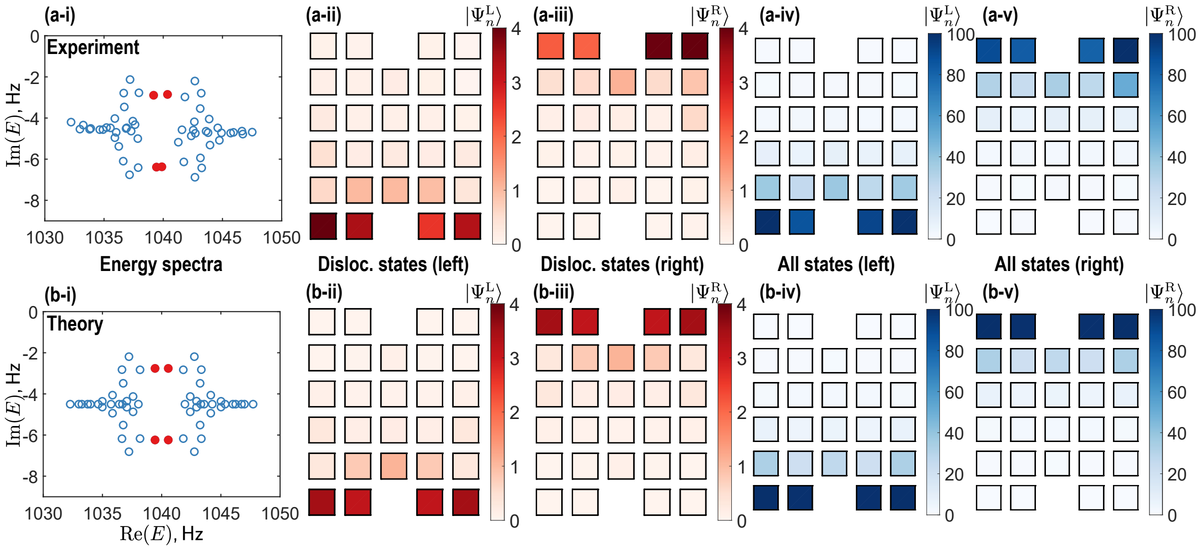

Figure S49: (a) Experimental results and (b) theoretical predictions of NH acoustic Chern insulators with an edge dislocation-antidislocation pair under OBCs in the M phase. (i) Energy spectra. Red dots ●, dislocation states; Blue circles ○, other states. Summed amplitude distributions of the (ii) left and (iii) right eigenstates for NHDS. Summed amplitude distributions of all (iv) left and (v) right eigenstates. Parameters: $\omega_0 = 1040\,\text{Hz} - 4.5\text{i}\,\text{Hz}, t_0 = -m_0 = 3\,\text{Hz}, h_x = 2.7\,\text{Hz}, h_y = h_z = 0$.

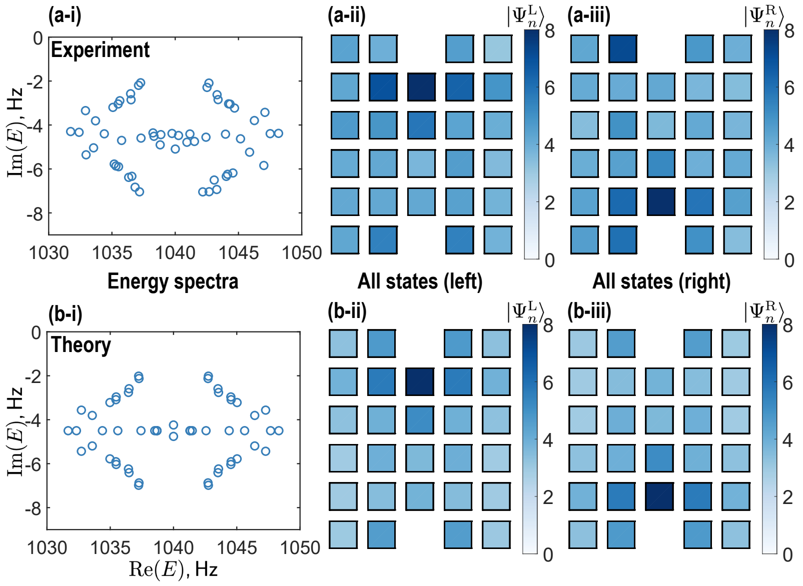

Figure S50: (a) Experimental results and (b) theoretical predictions of NH acoustic Chern insulators with an edge dislocation-antidislocation pair under PBCs in the Γ phase. (i) Energy spectra. Summed amplitude distributions of all (ii) left and (iii) right eigenstates. Parameters: $\omega_0 = 1040\,\text{Hz} - 4.5\text{i}\,\text{Hz}, t_0 = m_0 = 3\,\text{Hz}, h_y = 2.7\,\text{Hz}, h_x = h_z = 0$.

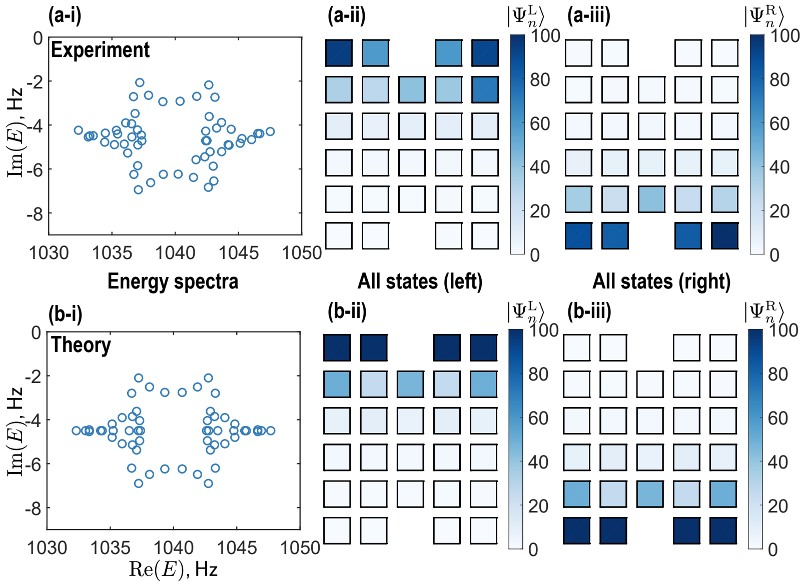

Figure S51: (a) Experimental results and (b) theoretical predictions of NH acoustic Chern insulators with an edge dislocation-antidislocation pair under OBCs in the Γ phase. (i) Energy spectra. Summed amplitude distributions of all (ii) left and (iii) right eigenstates. Parameters: $\omega_0 = 1040\,\text{Hz} - 4.5\text{i}\,\text{Hz}, t_0 = m_0 = 3\,\text{Hz}, h_x = 2.7\,\text{Hz}, h_y = h_z = 0$.

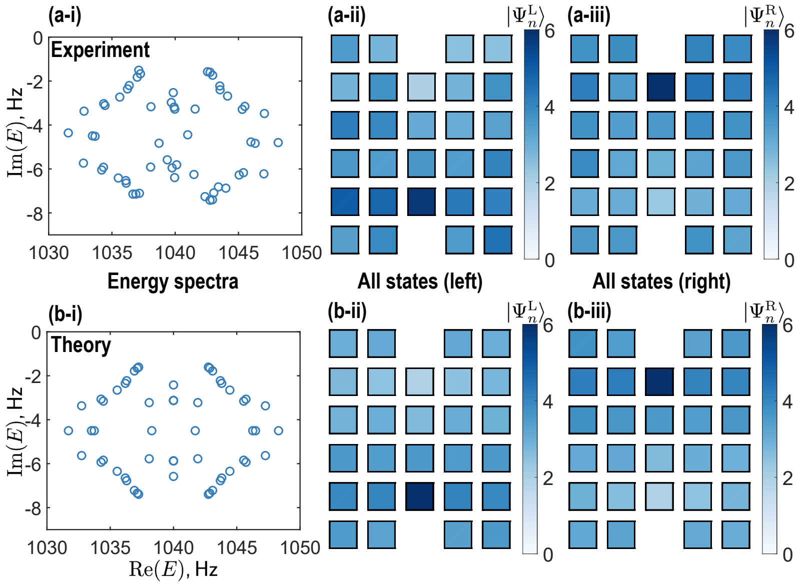

Figure S52: (a) Experimental results and (b) theoretical predictions of NH acoustic Chern insulators with an edge dislocation-antidislocation pair under PBCs in the M phase. (i) Energy spectra. Summed amplitude distributions of all (ii) left and (iii) right eigenstates. Parameters: $\omega_0 = 1040\,\mathrm{Hz} - 4.5\mathrm{i}\,\mathrm{Hz}, t_0 = -m_0 = 3\,\mathrm{Hz}, h_y = 3.3\,\mathrm{Hz}, h_x = h_z = 0$.

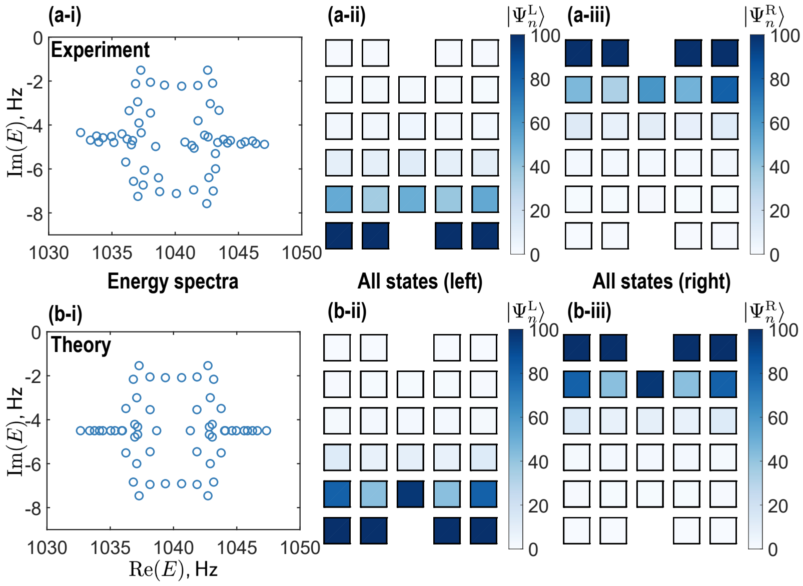

Figure S53: (a) Experimental results and (b) theoretical predictions of NH acoustic Chern insulators with an edge dislocation-antidislocation pair under OBCs in the M phase. (i) Energy spectra. Summed amplitude distributions of all (ii) left and (iii) right eigenstates. Parameters: $\omega_0 = 1040\,\mathrm{Hz} - 4.5\mathrm{i}\,\mathrm{Hz}, t_0 = -m_0 = 3\,\mathrm{Hz}, h_x = 3.3\,\mathrm{Hz}, h_y = h_z = 0$.

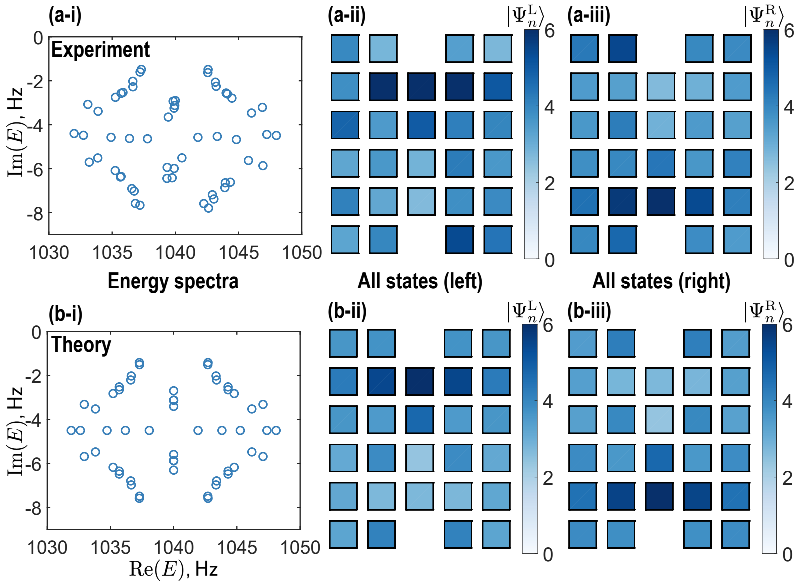

Figure S54: (a) Experimental results and (b) theoretical predictions of NH acoustic Chern insulators with an edge dislocation-antidislocation pair under PBCs in the Γ phase. (i) Energy spectra. Summed amplitude distributions of all (ii) left and (iii) right eigenstates. Parameters: $\omega_0 = 1040\,\text{Hz} - 4.5\text{i}\,\text{Hz}, t_0 = m_0 = 3\,\text{Hz}, h_y = 3.3, \text{Hz}, h_x = h_z = 0$.

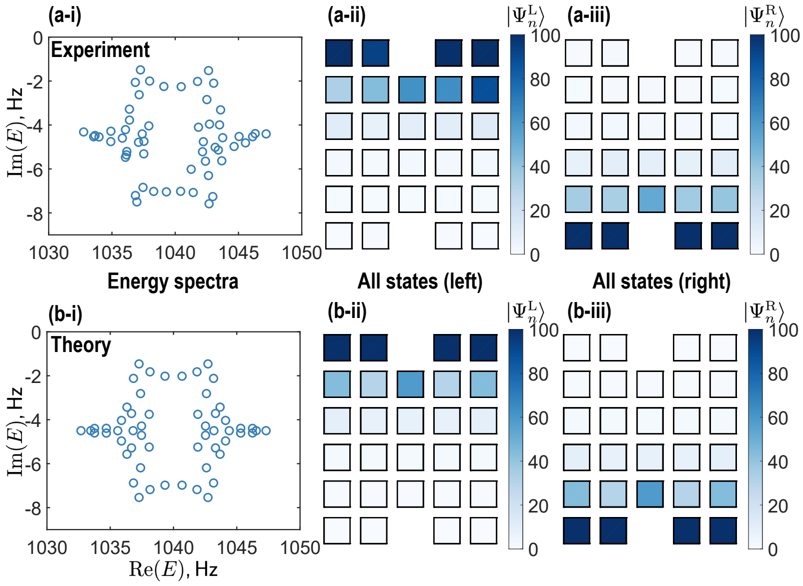

Figure S55: (a) Experimental results and (b) theoretical predictions of NH acoustic Chern insulators with an edge dislocation-antidislocation pair under OBCs in the Γ phase. (i) Energy spectra. (ii–iii) Summed amplitude distributions of all (ii) left and (iii) right eigenstates. Parameters: $\omega_0 = 1040\,\text{Hz} - 4.5\text{i}\,\text{Hz}, t_0 = m_0 = 3\,\text{Hz}, h_x = 3.3\,\text{Hz}, h_y = h_z = 0$.

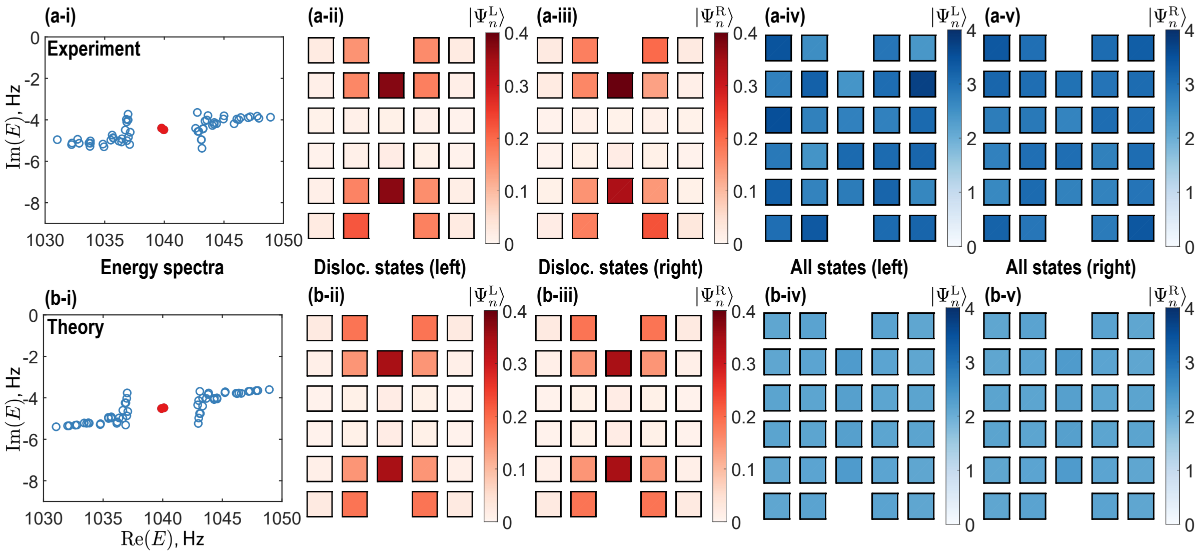

Figure S56: (a) Experimental results and (b) theoretical predictions of NH acoustic Chern insulators with an edge dislocation-antidislocation pair under PBCs in the M phase. (i) Energy spectra. Red dots ●, dislocation states; Blue circles ○, other states. Amplitude distributions of the (ii) left and (iii) right eigenstates for the dislocation states. Summed amplitude distributions of all (iv) left and (v) right eigenstates. Parameters: $\omega_0 = 1040\,\text{Hz} - 4.5\text{i}\,\text{Hz}, t_0 = -m_0 = 3\,\text{Hz}, h_z = 0.3\,\text{Hz}, h_x = h_y = 0$.

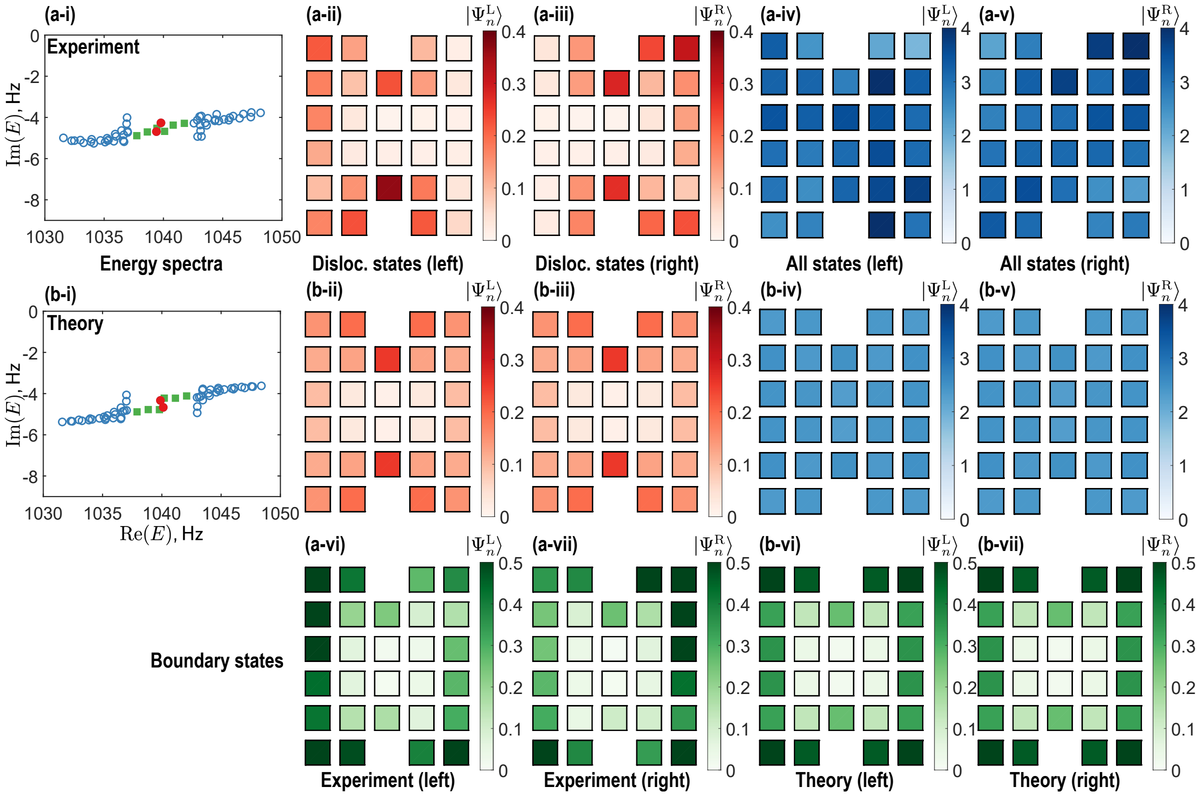

Figure S57: (a) Experimental results and (b) theoretical predictions of NH acoustic Chern insulators with an edge dislocation-antidislocation pair under OBCs in the M phase. (i) Energy spectra. Red dots ●, dislocation states; green squares ■, boundary states; blue circles ○, all other states. Amplitude distributions of the (ii) left and (iii) right eigenstates for the dislocation states. Summed amplitude distributions of all (iv) left and (v) right eigenstates. Amplitude distributions of the (vi) left and (vii) right eigenstates for the boundary states. Parameters: $\omega_0 = 1040\,\text{Hz} - 4.5\text{i}\,\text{Hz}, t_0 = -m_0 = 3\,\text{Hz}, h_z = 0.3\,\text{Hz}, h_x = h_y = 0$.

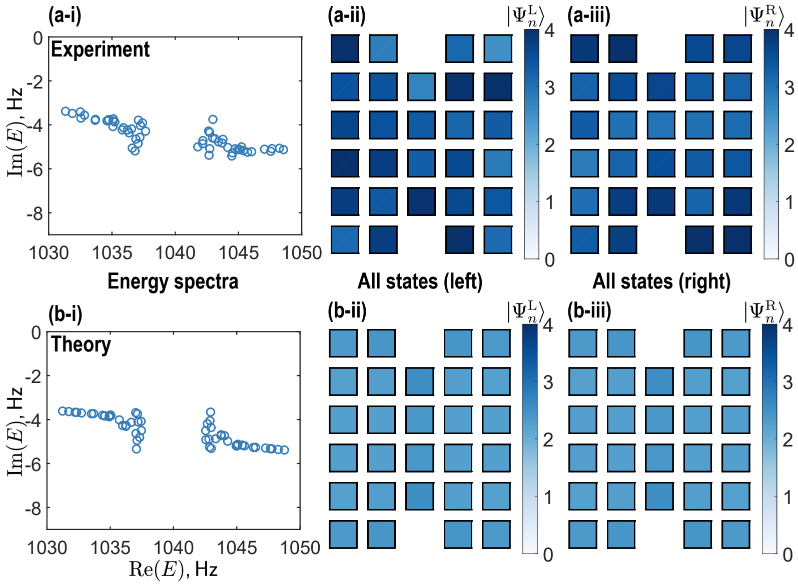

Figure S58: (a) Experimental results and (b) theoretical predictions of NH acoustic Chern insulators with an edge dislocation-antidislocation pair under PBCs in the Γ phase. (i) Energy spectra. Summed amplitude distributions of all (ii) left and (iii) right eigenstates. Parameters: $\omega_0 = 1040\,\mathrm{Hz} - 4.5\mathrm{i}\,\mathrm{Hz}, t_0 = m_0 = 3\,\mathrm{Hz}, h_z = 0.9\,\mathrm{Hz}, h_x = h_y = 0$.

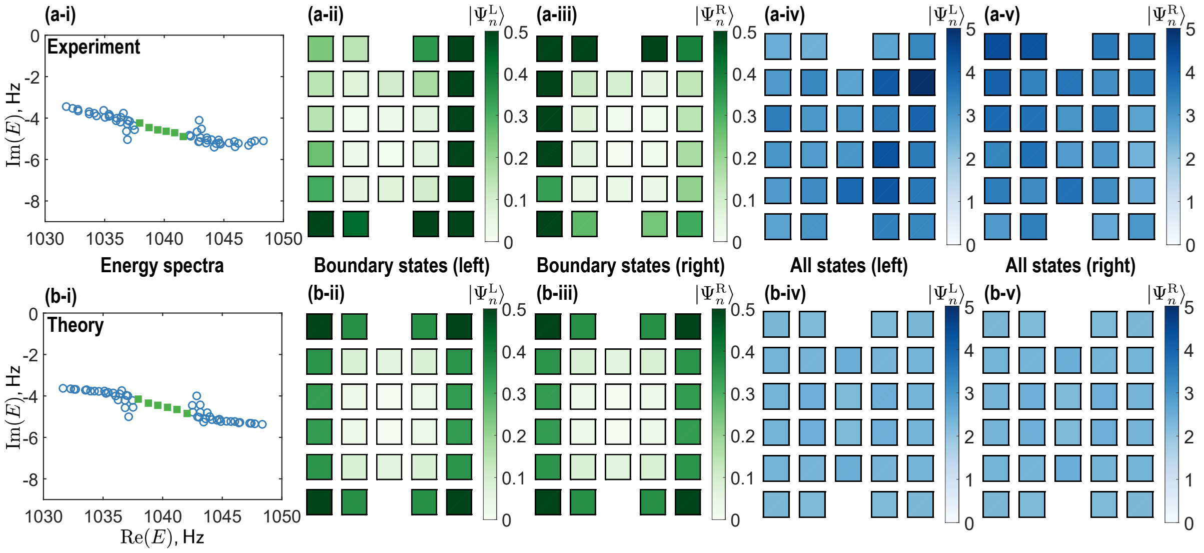

Figure S59: (a) Experimental results and (b) theoretical predictions of NH acoustic Chern insulators with an edge dislocation-antidislocation pair under OBCs in the Γ phase. (i) Energy spectra. Green squares ■, boundary states; Blue circles ○, other states. Amplitude distributions of the (ii) left and (iii) right eigenstates for the boundary states. Summed amplitude distributions of all (iv) left and (v) right eigenstates. Parameters: $\omega_0 = 1040\,\mathrm{Hz} - 4.5\mathrm{i}\,\mathrm{Hz}, t_0 = m_0 = 3\,\mathrm{Hz}, h_z = 0.3\,\mathrm{Hz}, h_y = h_z = 0$.

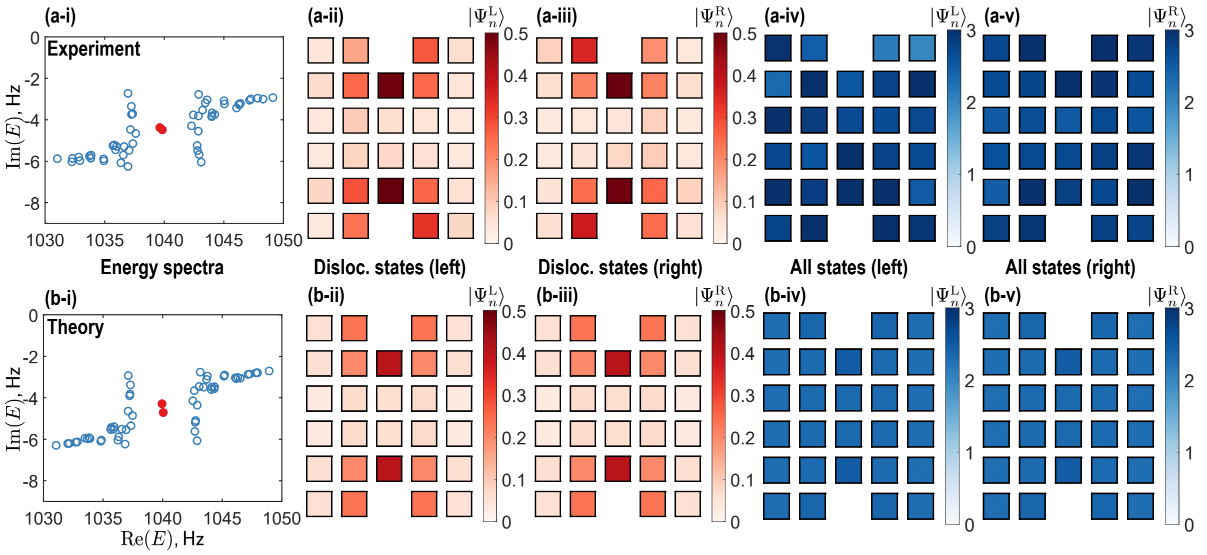

Figure S60: (a) Experimental results and (b) theoretical predictions of NH acoustic Chern insulators with an edge dislocation-antidislocation pair under PBCs in the M phase. (i) Energy spectra. Red dots ●, dislocation states; Blue circles ○, other states. Amplitude distributions of the (ii) left and (iii) right eigenstates for the dislocation states. Summed amplitude distributions of all (iv) left and (v) right eigenstates. Parameters: $\omega_0 = 1040\,\text{Hz} - 4.5\text{i}\,\text{Hz}, t_0 = -m_0 = 3\,\text{Hz}, h_z = 1.8\,\text{Hz}, h_x = h_y = 0$.

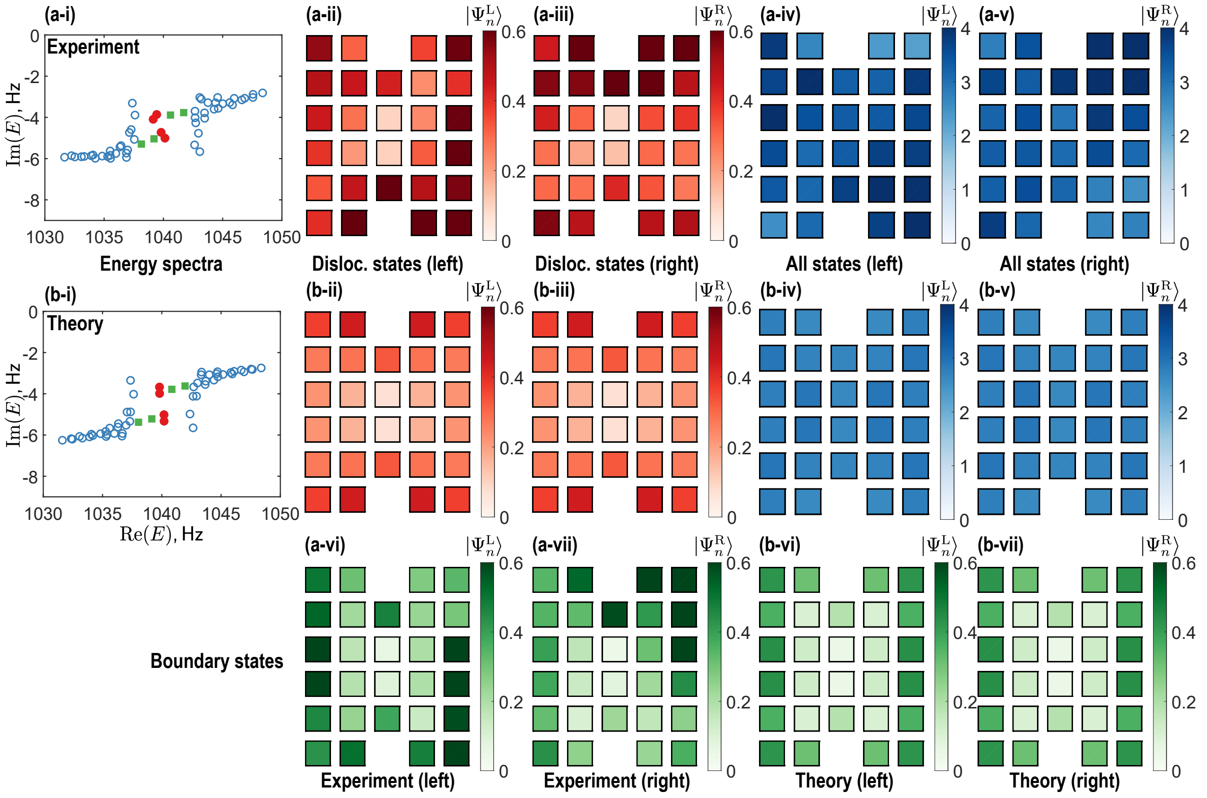

Figure S61: (a) Experimental results and (b) theoretical predictions of NH acoustic Chern insulators with an edge dislocation-antidislocation pair under OBCs in the M phase. (i) Energy spectra. Red dots ●, dislocation states; Blue circles ○, other states; Green squares ■, boundary states. Summed amplitude distributions of the (ii) left and (iii) right eigenstates for the dislocation states. Summed amplitude distributions of all (iv) left and (v) right eigenstates. Amplitude distributions of the (vi) left and (vii) right eigenstates for the boundary state. Parameters: $\omega_0 = 1040\,\text{Hz} - 4.5\text{i}\,\text{Hz}, t_0 = -m_0 = 3\,\text{Hz}, h_z = 1.8\,\text{Hz}, h_x = h_y = 0$.

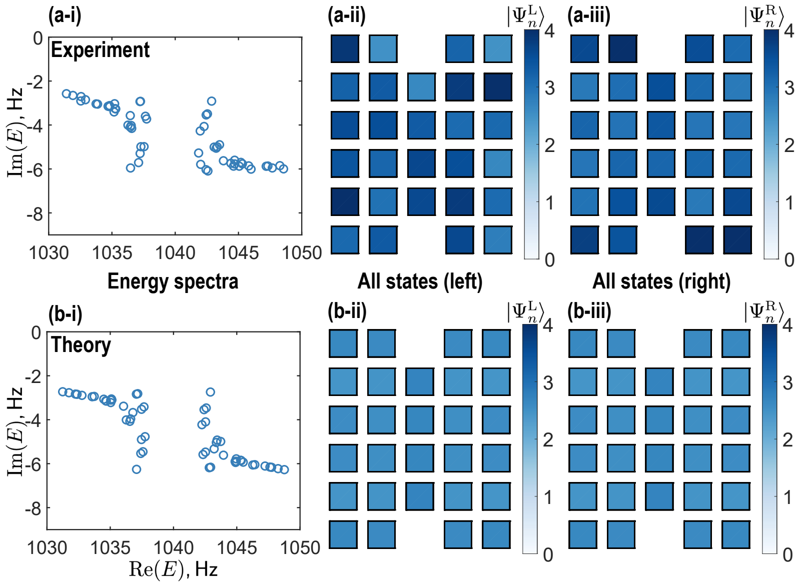

Figure S62: (a) Experimental results and (b) theoretical predictions of NH acoustic Chern insulators with an edge dislocation-antidislocation pair under PBCs in the Γ phase. (i) Energy spectra. Summed amplitude distributions of all (ii) left and (iii) right eigenstates. Parameters: $\omega_0 = 1040\,\mathrm{Hz} - 4.5\mathrm{i}\,\mathrm{Hz}, t_0 = m_0 = 3\,\mathrm{Hz}, h_z = 1.8\,\mathrm{Hz}, h_x = h_y = 0$.

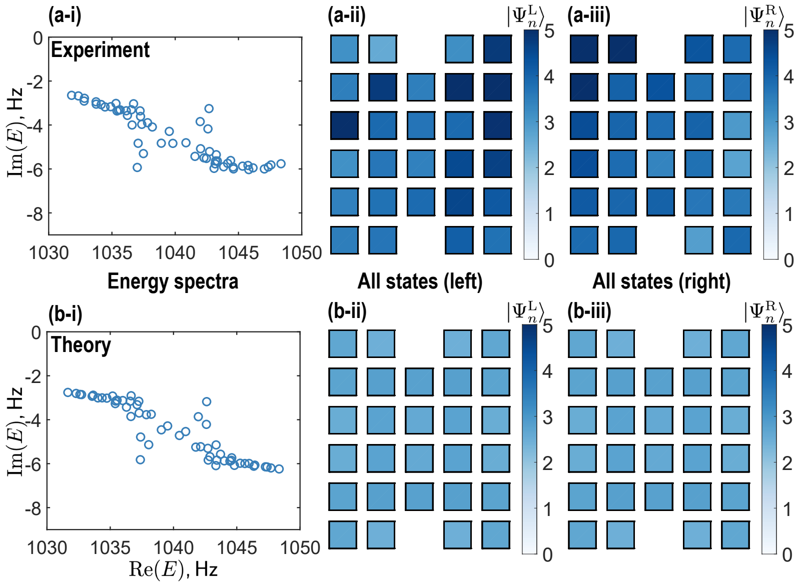

Figure S63: (a) Experimental results and (b) theoretical predictions of NH acoustic Chern insulators with an edge dislocation-antidislocation pair under OBCs in the Γ phase. (i) Energy spectra. Summed amplitude distributions of all (ii) left and (iii) right eigenstates. Parameters: $\omega_0 = 1040\,\mathrm{Hz} - 4.5\mathrm{i}\,\mathrm{Hz}, t_0 = m_0 = 3\,\mathrm{Hz}, h_z = 1.8\,\mathrm{Hz}, h_x = h_y = 0$.

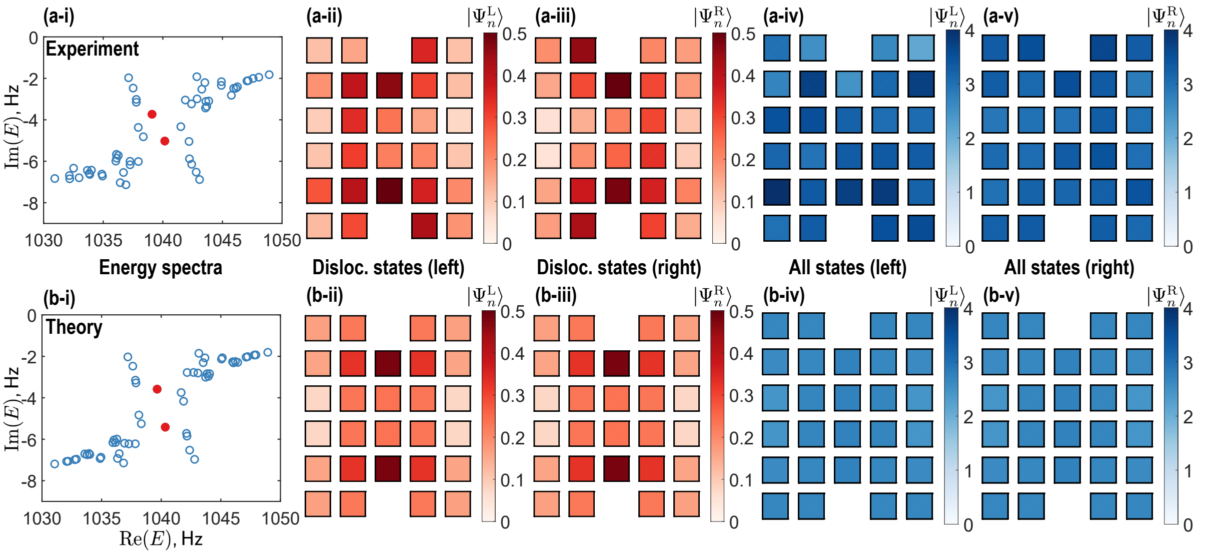

Figure S64: (a) Experimental results and (b) theoretical predictions of NH acoustic Chern insulators with an edge dislocation-antidislocation pair under PBCs in the M phase. (i) Energy spectra. Red dots ●, dislocation states; Blue circles ○, other states. Amplitude distributions of the (ii) left and (iii) right eigenstates for the dislocation states. Summed amplitude distributions of all (iv) left and (v) right eigenstates. Parameters: $\omega_0 = 1040\,\mathrm{Hz} - 4.5\mathrm{i}\,\mathrm{Hz}, t_0 = -m_0 = 3\,\mathrm{Hz}, h_z = 2.7\,\mathrm{Hz}, h_x = h_y = 0$.

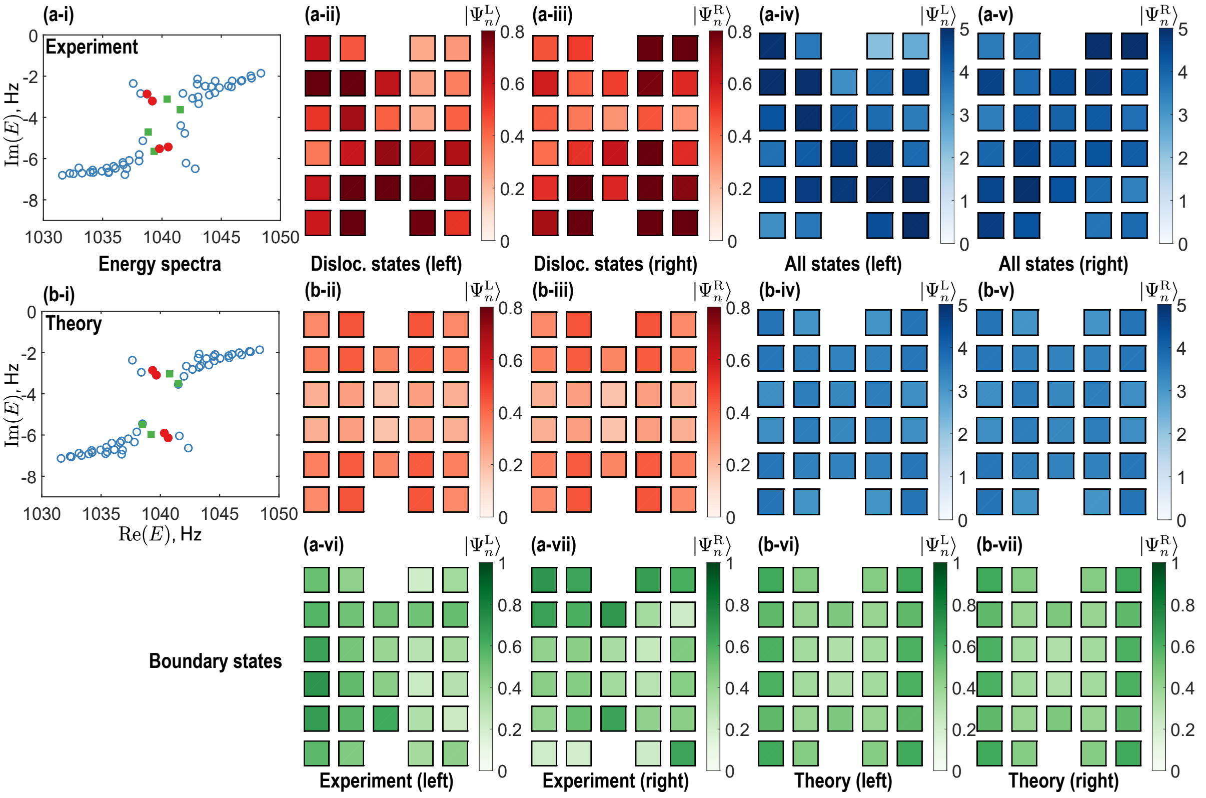

Figure S65: (a) Experimental results and (b) theoretical predictions of NH acoustic Chern insulators with an edge dislocation-antidislocation pair under OBCs in the M phase. (i) Energy spectra. Red dots ●, dislocation states; Blue circles ○, other states; Green squares ■, boundary states. Summed amplitude distributions of the (ii) left and (iii) right eigenstates for the dislocation states. Summed amplitude distributions of all (iv) left and (v) right eigenstates. Amplitude distributions of the (vi) left and (vii) right eigenstates for the boundary state. Parameters: $\omega_0 = 1040\,\mathrm{Hz} - 4.5\mathrm{i}\,\mathrm{Hz}, t_0 = -m_0 = 3\,\mathrm{Hz}, h_z = 2.7\,\mathrm{Hz}, h_x = h_y = 0$.

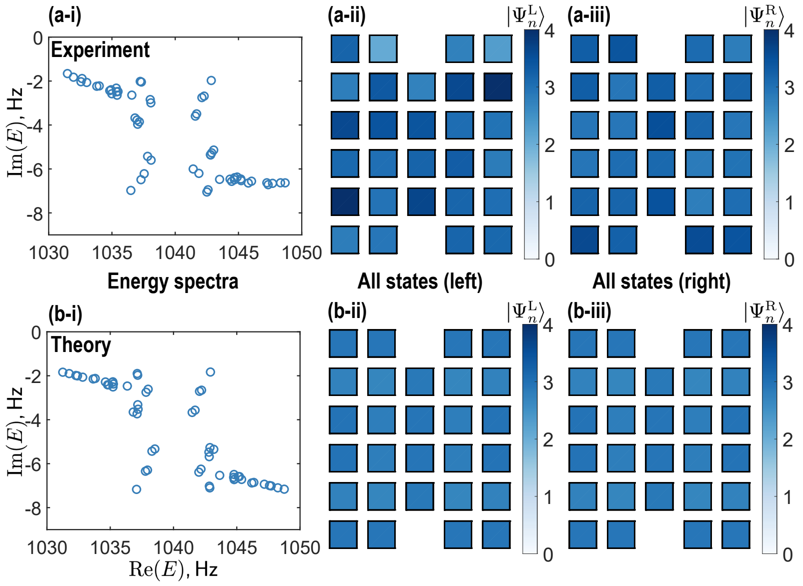

Figure S66: (a) Experimental results and (b) theoretical predictions of NH acoustic Chern insulators with an edge dislocation-antidislocation pair under PBCs in the Γ phase. (i) Energy spectra. Summed amplitude distributions of all (ii) left and (iii) right eigenstates. Parameters: $\omega_0 = 1040\,\mathrm{Hz} - 4.5\mathrm{i}\,\mathrm{Hz}, t_0 = m_0 = 3\,\mathrm{Hz}, h_z = 2.7\,\mathrm{Hz}, h_x = h_y = 0$.

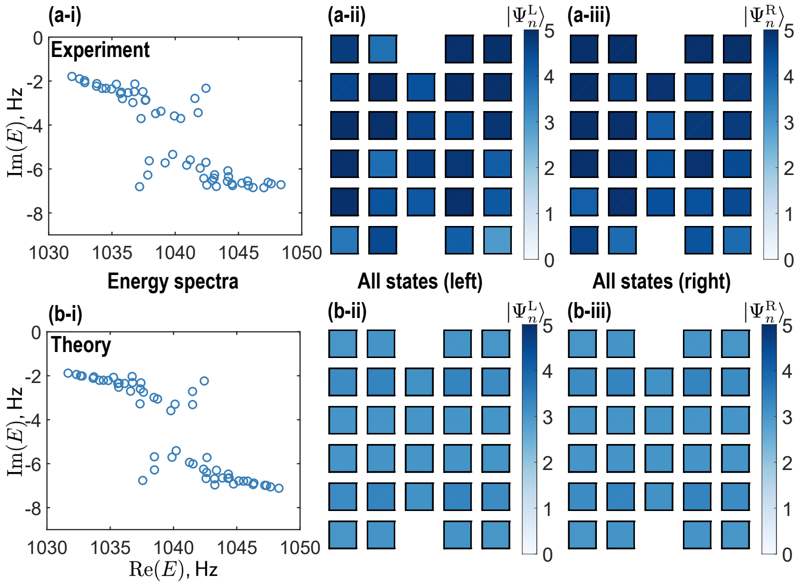

Figure S67: (a) Experimental results and (b) theoretical predictions of NH acoustic Chern insulators with an edge dislocation-antidislocation pair under OBCs in the Γ phase. (i) Energy spectra. Summed amplitude distributions of all (ii) left and (iii) right eigenstates. Parameters: $\omega_0 = 1040\,\mathrm{Hz} - 4.5\mathrm{i}\,\mathrm{Hz}, t_0 = m_0 = 3\,\mathrm{Hz}, h_z = 2.7\,\mathrm{Hz}, h_x = h_y = 0$.

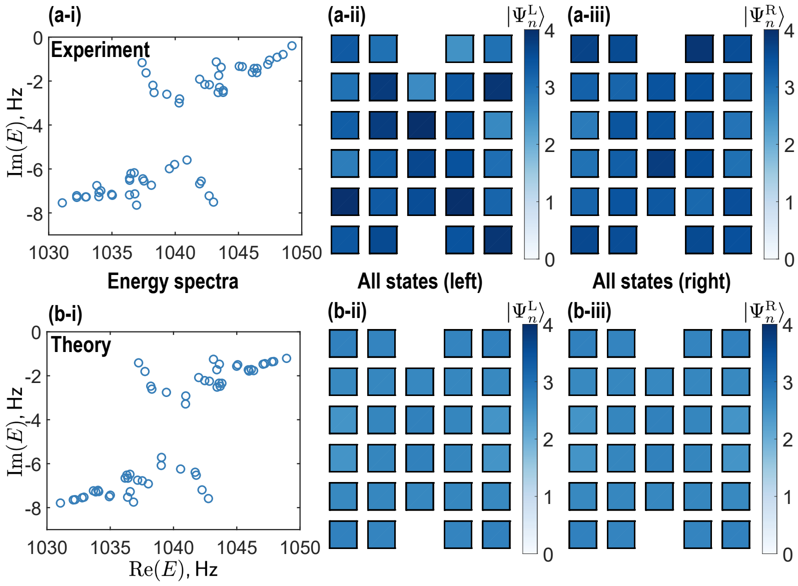

Figure S68: (a) Experimental results and (b) theoretical predictions of NH acoustic Chern insulators with an edge dislocation-antidislocation pair under OBCs in the M phase. (i) Energy spectra. Summed amplitude distributions of all (ii) left and (iii) right eigenstates. Parameters: $\omega_0 = 1040\,\mathrm{Hz} - 4.5\mathrm{i}\,\mathrm{Hz}, t_0 = -m_0 = 3\,\mathrm{Hz}, h_z = 3.3\,\mathrm{Hz}, h_x = h_y = 0$.

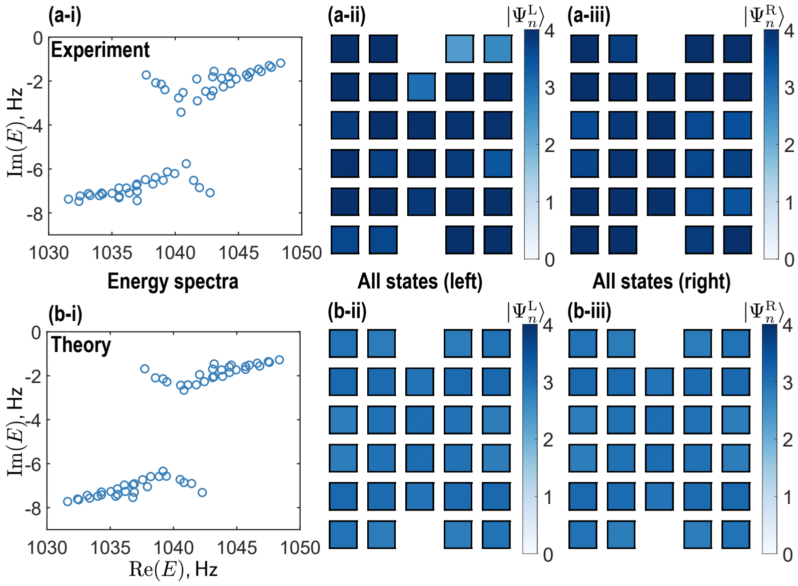

Figure S69: (a) Experimental results and (b) theoretical predictions of NH acoustic Chern insulators with an edge dislocation-antidislocation pair under OBCs in the M phase. (i) Energy spectra. Summed amplitude distributions of all (ii) left and (iii) right eigenstates. Parameters: $\omega_0 = 1040\,\mathrm{Hz} - 4.5\mathrm{i}\,\mathrm{Hz}, t_0 = -m_0 = 3\,\mathrm{Hz}, h_z = 3.3\,\mathrm{Hz}, h_x = h_y = 0$.

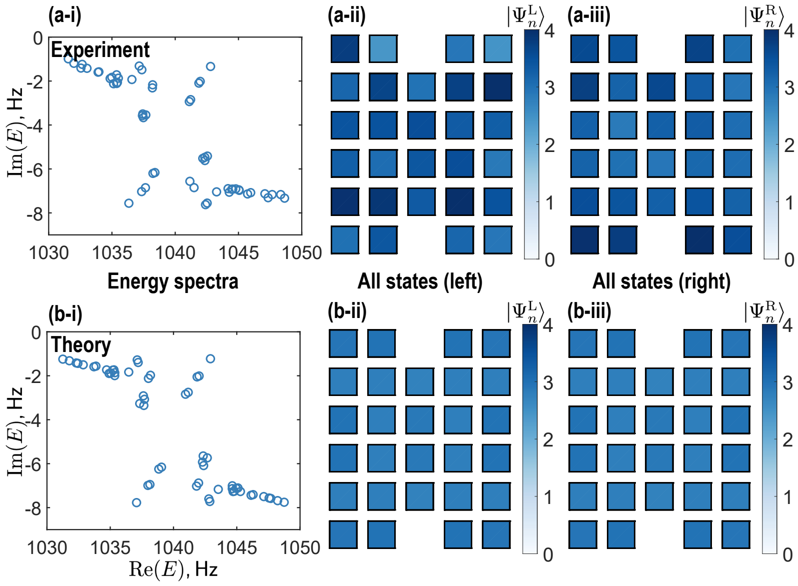

Figure S70: (a) Experimental results and (b) theoretical predictions of NH acoustic Chern insulators with an edge dislocation-antidislocation pair under PBCs in the Γ phase. (i) Energy spectra. Summed amplitude distributions of all (ii) left and (iii) right eigenstates. Parameters: $\omega_0 = 1040\,\mathrm{Hz} - 4.5\mathrm{i}\,\mathrm{Hz}, t_0 = m_0 = 3\,\mathrm{Hz}, h_z = 3.3\,\mathrm{Hz}, h_x = h_y = 0$.

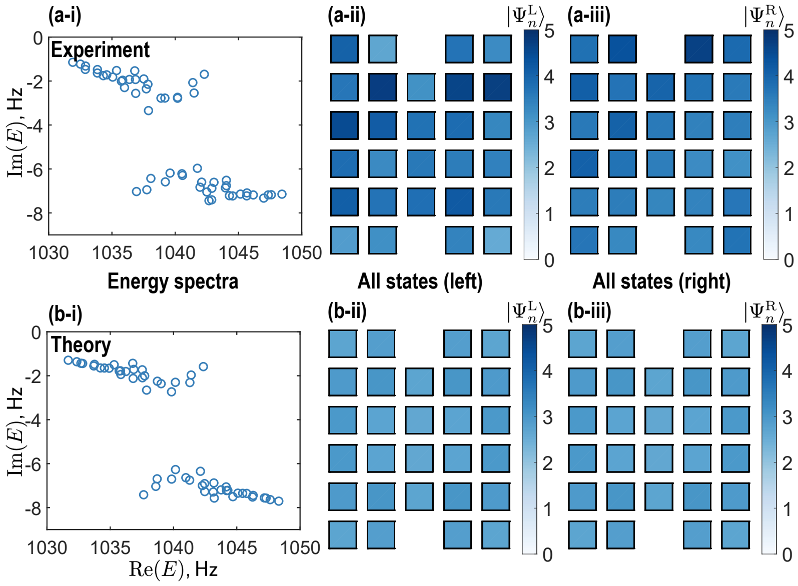

Figure S71: (a) Experimental results and (b) theoretical predictions of NH acoustic Chern insulators with an edge dislocation-antidislocation pair under OBCs in the Γ phase. (i) Energy spectra. Summed amplitude distributions of all (ii) left and (iii) right eigenstates. Parameters: $\omega_0 = 1040\,\mathrm{Hz} - 4.5\mathrm{i}\,\mathrm{Hz}, t_0 = m_0 = 3\,\mathrm{Hz}, h_z = 3.3\,\mathrm{Hz}, h_x = h_y = 0$.

References

[1] J.-X. Zhong et al. "Experimentally Probing Non-Hermitian Spectral Transition and Eigenstate Skewness". In: *Phys. Rev. B* 112.22 (2025), p. L220301.

[2] J.-X. Zhong et al. "Higher-Order Skin Effect and Its Observation in an Acoustic Kagome Lattice". In: *Phys. Rev. B* 111.1 (2025), p. 014314.

[3] J.-X. Zhong et al. *Experimentally Probing Non-Hermitian Spectral Transition and Eigenstate Skewness*. 2025. URL: http://arxiv.org/abs/2501.08160 (visited on 02/19/2025). Pre-published.

[4] W. Wu et al. "Observation of Dislocation non-Hermitian Skin Effect in a Torus-like Acoustic Metamaterial". In: *Adv. Mater.* (2025), e14101.

[5] L. Xiong et al. "Tracking Intrinsic Non-Hermitian Skin Effects in Lossy Lattices". In: *Phys. Rev. B* 110.14 (2024), p. L140305.
